# Menstrual cup use, leakage, acceptability, safety, and availability: a systematic review and meta-analysis

**DOI:** 10.1016/S2468-2667(19)30111-2

**Published:** 2019-07-16

**Authors:** Anna Maria van Eijk, Garazi Zulaika, Madeline Lenchner, Linda Mason, Muthusamy Sivakami, Elizabeth Nyothach, Holger Unger, Kayla Laserson, Penelope A Phillips-Howard

**Affiliations:** a Department of Clinical Sciences, Liverpool School of Tropical Medicine, Liverpool, UK; b Centre for Maternal and Newborn Health, Liverpool School of Tropical Medicine, Liverpool, UK; c Population Health Sciences, Institute of Epidemiology and Health Care, University College London, London, UK; d Centre for Health and Social Sciences, School of Health Systems Studies, Tata Institute of Social Sciences, Mumbai, Maharashtra, India; e Centre for Global Health Research, Kenya Medical Research Institute (KEMRI), Kisumu, Kenya; f Department of Obstetrics and Gynaecology, Edinburgh Royal Infirmary, Edinburgh, UK; g Bill & Melinda Gates Foundation, India Country Office, New Delhi, India

## Abstract

**Background:**

Girls and women need effective, safe, and affordable menstrual products. Single-use products are regularly selected by agencies for resource-poor settings; the menstrual cup is a less known alternative. We reviewed international studies on menstrual cup leakage, acceptability, and safety and explored menstrual cup availability to inform programmes.

**Methods:**

In this systematic review and meta-analysis, we searched PubMed, Cochrane Library, Web of Science, Popline, Cinahl, Global Health database, Emerald, Google Scholar, Science.gov, and WorldWideScience from database inception to May 14, 2019, for quantitative or qualitative studies published in English on experiences and leakage associated with menstrual cups, and adverse event reports. We also screened the Manufacturer and User Facility Device Experience database from the US Food and Drug Administration for events related to menstrual cups. To be eligible for inclusion, the material needed to have information on leakage, acceptability, or safety of menstrual cups. The main outcome of interest was menstrual blood leakage when using a menstrual cup. Safety outcomes of interest included serious adverse events; vaginal abrasions and effects on vaginal microflora; effects on the reproductive, digestive, or urinary tract; and safety in poor sanitary conditions. Findings were tabulated or combined by use of forest plots (random-effects meta-analysis). We also did preliminary estimates on costs and environmental savings potentially associated with cups. This systematic review is registered on PROSPERO, number CRD42016047845.

**Findings:**

Of 436 records identified, 43 studies were eligible for analysis (3319 participants). Most studies reported on vaginal cups (27 [63%] vaginal cups, five [12%] cervical cups, and 11 [25%] mixed types of cups or unknown) and 15 were from low-income and middle-income countries. 22 studies were included in qualitative or quantitative syntheses, of which only three were of moderate-to-high quality. Four studies made a direct comparison between menstrual cups and usual products for the main outcome of leakage and reported leakage was similar or lower for menstrual cups than for disposable pads or tampons (n=293). In all qualitative studies, the adoption of the menstrual cup required a familiarisation phase over several menstrual cycles and peer support improved uptake (two studies in developing countries). In 13 studies, 73% (pooled estimate: n=1144; 95% CI 59–84, *I*^2^=96%) of participants wished to continue use of the menstrual cup at study completion. Use of the menstrual cup showed no adverse effects on the vaginal flora (four studies, 507 women). We identified five women who reported severe pain or vaginal wounds, six reports of allergies or rashes, nine of urinary tract complaints (three with hydronephrosis), and five of toxic shock syndrome after use of the menstrual cup. Dislodgement of an intrauterine device was reported in 13 women who used the menstrual cup (eight in case reports, and five in one study) between 1 week and 13 months of insertion of the intrauterine device. Professional assistance to aid removal of menstrual cup was reported among 47 cervical cup users and two vaginal cup users. We identified 199 brands of menstrual cup, and availability in 99 countries with prices ranging US$0·72–46·72 (median $23·3, 145 brands).

**Interpretation:**

Our review indicates that menstrual cups are a safe option for menstruation management and are being used internationally. Good quality studies in this field are needed. Further studies are needed on cost-effectiveness and environmental effect comparing different menstrual products.

**Funding:**

UK Medical Research Council, Department for International Development, and Wellcome Trust.

## Introduction

Girls and women need effective, safe, and affordable menstrual products. Globally, an estimated 1·9 billion women—around 26% of the population—were of menstruating age in 2017, spending on average 65 days in the year dealing with menstrual blood flow.^1^ Menstruation is a normal body function and a sign of reproductive health. Few solutions are available to manage menstruation; additionally, ignorance, prejudice, costs, and safety fears can impede girls and women from testing the full range of products available. A lack of affordable and effective menstrual products can result in leakage and chaffing in menstruating girls and women and can affect their health.^2^, ^3^ Use of poor-quality materials has been shown to predispose women to an increased risk of urogenital infections including bacterial vaginosis.^4^, ^5^, ^6^ In some situations, mostly researched in low-income and middle-income countries, menstruation can affect girls' schooling,^7^ make women and girls a target of sexual violence or coercion,^8^, ^9^ and affect employment and work experiences of women.^10^, ^11^ In low-income and middle-income countries, a lack of water, sanitation, and hygiene, inadequate education, and poor disposal facilities, raise public health concerns, particularly among schoolgirls.^7^, ^12^ In several countries, the number of policy-led initiatives and donations to provide menstrual products have increased—eg, to keep girls in school. To allow such organisations to make informed decisions, information is needed on the full range of menstrual products.

**Research in context**

**Evidence before this study**

A lack of affordable and effective menstrual products can result in leakage and chaffing in menstruating girls and women and can affect their health and education. The number of programmes that provide menstrual products to assist women and girls has increased. The menstrual cup, a receptacle used to collect menstrual blood flow, has received little attention, which in part might reflect concerns about insertable products as either culturally unacceptable or because of previous public health alerts associated with highly absorbent tampons (eg, toxic-shock syndrome). Information about leakage, acceptability, and safety of menstrual cups is needed to support organisations to make informed decisions and provide more comprehensive menstrual health education for girls and women. We searched PubMed, Cochrane Library, Web of Science, Popline, Cinahl, Global Health database, Emerald, Google Scholar, Science.gov, and WorldWideScience from database inception on Nov 24, 2018, for publications in English using the keywords (“Menstrual Cup”) AND “Review” to determine if a review of menstrual cups was available with information on leakage, acceptability, and safety. No review was identified, but a literature review on menstrual management in emergency contexts noted a lack of empirical evidence examining the introduction and testing of menstrual cups in humanitarian settings.

**Added value of this study**

To our knowledge, this is the first systematic review and meta-analysis examining girls' and women's experiences of menstrual cups, aggregating outcomes from 43 studies and 3319 participants who were asked about their use or willingness to use menstrual cups. We provide information on leakage compared with other products, a listing of known adverse events, and quantitative and qualitative information on acceptability in both high-income countries and low-income and middle-income countries. We also assessed availability and prices of menstrual cups. Serious adverse events were not common, with five reported cases of toxic-shock syndrome. However, the number of menstrual cup users is unknown, so comparisons of risk of toxic-shock syndrome between menstrual cups, tampons, or the intravaginal diaphragm cannot be made. Although menstrual cups are manufactured and available globally, they are not commonly mentioned on websites offering educational materials on puberty for girls.

**Implications of all the available evidence**

Menstrual cups seem to be an effective and safe alternative to other menstrual products. Information on menstrual cups should be provided in puberty education materials. Policy makers and programmes can consider this product as an option in menstrual health programmes. Further research globally can provide more information on acceptability and is needed to monitor adverse events and assess best practice to shorten the familiarisation phase required for safe and effective use, and on cost-effectiveness and environmental effects.

The menstrual cup is not commonly known, despite its long history (appendix p 2).^13^ Like tampons, menstrual cups are inserted into the vagina, but the blood is collected in the receptacle, which can hold 10–38 mL of blood. The menstrual cup should be emptied every 4–12 h, depending on menstrual flow and type of cup. Two types of cup are available, a vaginal cup, which is generally bell-shaped and placed in the vagina, and a cervical cup, which, like a diaphragm for contraception, is placed around the cervix high in the vagina. Menstrual cups are made of medical-grade silicone, rubber, latex, or elastomer and can last up to 10 years; disposable single-use menstrual cups also exist.

We aimed to summarise current knowledge about leakage, safety, and acceptability of menstrual cups and compared, when available, with other menstrual products. We compiled information on global availability and costs of menstrual cups, did preliminary estimates on costs and waste savings, and examined online guidance materials on menarche in selected regions of the world for reference to menstrual cup as a product option.

## Methods

### Search strategy and selection criteria

In this systematic review and meta-analysis, we searched PubMed, Cochrane Library, Web of Science, Popline, Cinahl, Global Health database, Emerald, Google Scholar, Science.gov, and WorldWideScience for material in English from the inception of the database until May 14, 2019, using the keywords (“menstrual” AND “cup”) OR (“menses” AND “cup”) OR (“menstruation” AND “cup”) OR (“vaginal” AND “cup”). We also screened the Manufacturer and User Facility Device Experience (MAUDE) database from the US Food and Drug Administration (FDA) for events related to menstrual cups (10-year limit, last search done on May 28, 2019).^14^ For information on costs and availability, we screened websites of menstrual cup manufacturers using different web listings and web searches and consulted experts (full lists are in the appendix [pp 35–39]).

To ensure we covered a broad range of the available literature, we searched the reference lists of relevant studies, websites of pertinent professional bodies (eg, FDA), non-governmental organisations, and grey literature (eg, reports or conference abstracts), and we contacted experts in the field to recommend relevant reports. For information on costs and availability, our search included individually going through every list of menstrual cup brands we could find and searching where they were being sold (via web lists, Google searches, Pinterest boards, Facebook pages, and experts working in countries where cups appeared to be unavailable to confirm).

Study eligibility, data extraction, and risk-of-bias assessment were done independently by two reviewers (AMvE and ML for quantitative and LM and GZ for qualitative studies), and conflicts were resolved via discussion until an agreement was reached. To be eligible for inclusion, the material needed to have information on leakage, acceptability, or safety of menstrual cups. Quantitative, qualitative, or mixed design studies were included. Animal studies, and studies using menstrual cups to collect vaginal fluids without participants' reported experiences during menstruation were ineligible.

The main outcome of interest was menstrual blood leakage when using the menstrual cup. Additional outcomes of interest were acceptability of use of menstrual cups, difficulty with insertion or removal, comfort of wearing, and intention to use in future. Safety outcomes of interest included serious adverse events, such as toxic shock syndrome; vaginal abrasions and effects on vaginal microflora (eg, vaginal discharge, infections); effects on the reproductive, digestive, or urinary tract; and safety in poor sanitary conditions. Other safety issues we identified only during our review were documented, and all material was re-reviewed to ensure completeness of the safety assessment.

### Data analysis

Data were manually extracted from studies using spreadsheets. If the same results from the same study were presented in several reports, we used data from the report with the largest sample size. For quality and bias assessments, we used the Cochrane tool for trials, an adaptation of the Newcastle-Ottawa tool (appendix p 5) for observational studies,^15^, ^16^ and the Critical Appraisal Skills Programme tool^17^ for qualitative studies.

We tabulated our findings as a narrative synthesis. If trials or studies presented sufficiently homogeneous data in terms of design, we pooled results as proportions using meta-analyses and a random-effects model with heterogeneity quantified using the *I*^2^ statistic (appendix p 3). We examined the following sources of heterogeneity if sufficient data were available using subgroup analysis: setting of the study (high-income *vs* low-income and middle-income countries), study population (adult women *vs* adolescents), year of study (study conducted before or after 2000), type of menstrual cup used (cervical *vs* vaginal cup), and duration of menstrual cup use. We assessed publication and small-study bias by visual inspection of funnel plots and Egger's test. We integrated the quantitative and qualitative analyses for the acceptability of use of menstrual cups.

For estimations on costs of disposable pads and tampons, we explored prices for commonly used products in six countries (the USA, the UK, India, Spain, China, and Canada) and calculated average costs per product. Extrapolating information on content and weight of menstrual products,^18^ we estimated waste and costs for a range of 9–25 units per product per month and compared these with consistent use of one menstrual cup for 10 years. Additional information on methods used to assess menstrual cup information, availability and prices, qualitative studies, and costs and waste, and additional information on data extraction are in the appendix (pp 3–5).

We did a sensitivity analysis of low versus moderate-to-good quality studies, as determined by the quality assessment and assessed small-study effect using funnel plots and the Egger's test. We used two-tailed p values of less than 0·05 to indicate statistical significance. We did statistical analyses using Metaprop, Stata version 14.2.2. This systematic review is registered on PROSPERO, number CRD42016047845.

### Role of the funding source

The funders had no role in study design, data collection, data analysis, data interpretation, or writing of the report. The corresponding author had full access to all data in the study and had final responsibility to submit for publication.

## Results

**Figure 1:**
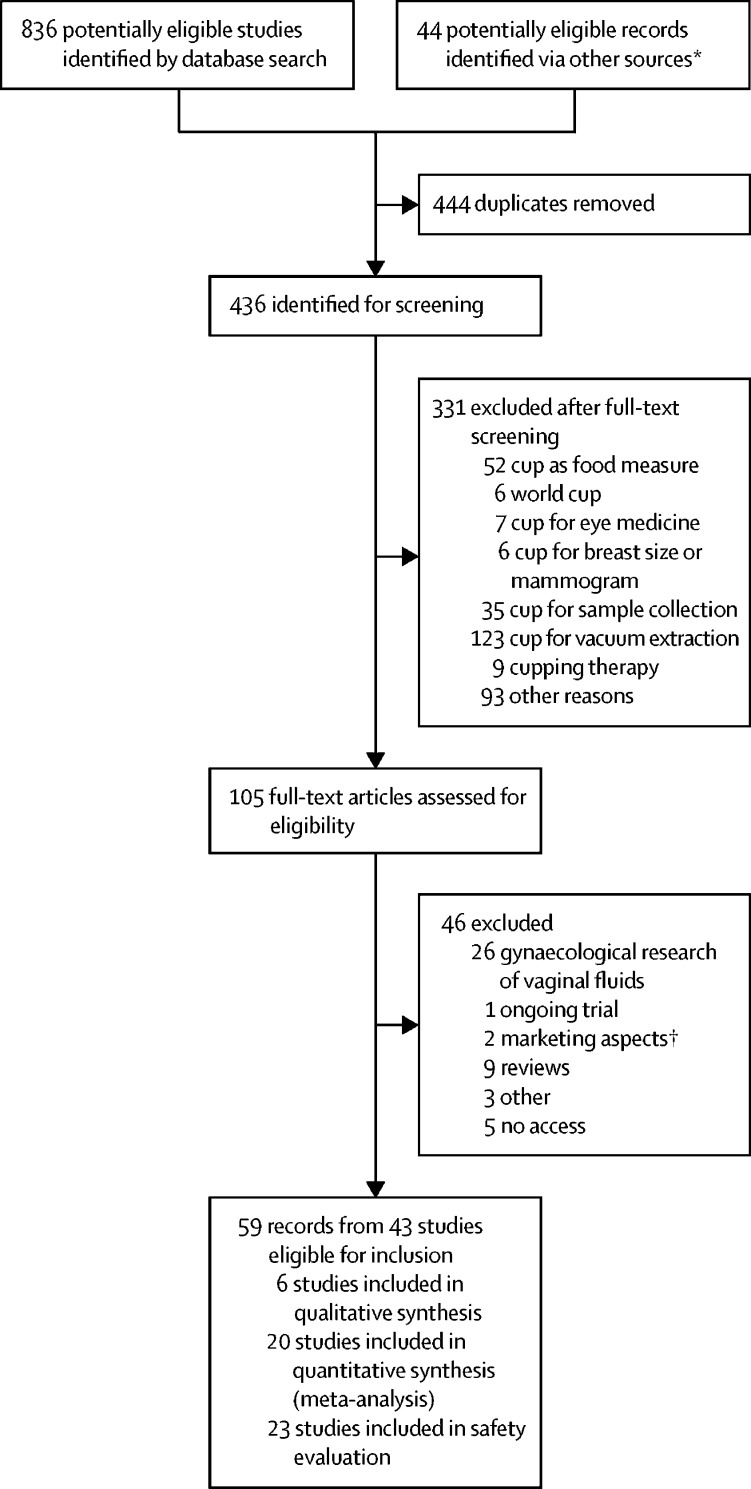
Study selection *Reference lists of relevant studies, websites of pertinent professional bodies (eg, US Food and Drug Administration), non-governmental organisations, grey literature (eg, reports or conference abstracts), and records recommended by experts. †For example, advertising approaches.

Of 436 unique records identified (appendix p 6), 59 were identified as relevant (figure 1), and 43 studies were included in our analysis (table 1). In these 43 studies, 3319 participants used or were asked about the menstrual cup.^5^, ^13^, ^14^, ^19^, ^20^, ^21^, ^22^, ^23^, ^24^, ^25^, ^26^, ^27^, ^28^, ^29^, ^30^, ^31^, ^32^, ^33^, ^34^, ^35^, ^36^, ^37^, ^38^, ^39^, ^40^, ^41^, ^42^, ^43^, ^44^, ^45^, ^46^, ^47^, ^48^, ^49^, ^50^, ^51^, ^52^, ^53^, ^54^, ^55^, ^56^, ^57^, ^58^, ^59^, ^60^, ^61^, ^62^, ^63^, ^64^, ^65^, ^66^, ^67^, ^68^, ^69^, ^70^, ^71^, ^72^, ^73^ Seven studies were completed among schoolgirls (ie, aged 12–19 years) in low-income and middle-income countries (647 [19·5%] participants).^5^, ^27^, ^33^, ^43^, ^58^, ^59^ Three studies were done in the early 1960s, six in the late 1980s, and 26 in 2009–18. 15 studies were from low-income and middle-income countries. Most studies reported on vaginal cups (27 [63%] vaginal cups, five [12%] cervical cups, and 11 [25%] mixed types of cups or unknown) and 35 (81%) were journal articles. Although some studies did not report the type or brand of menstrual cup used, at least seven described menstrual cups that are no longer available (Tassette, Tassaway, and Gynaeseal). The quality of quantitative studies was low, with only two that were of moderate-to-high quality (table 1; appendix pp 7–8). Many studies did not clearly identify where their participants were from, or participants were not representative of the community. Only six studies, all from low-income and middle-income countries, provided qualitative information (appendix p 10).

**Table 1:** Characteristics of studies contributing to menstrual cup review

	**Source and study design**	**Location and date**	**Sample size and population**	**Age and heavy menstrual flow as defined by source**	Menstrual cup brand*(type)	**Comparison**	**Follow-up**	**Outcomes**	**Loss to follow-up (%)**	**Quality score**
**Trials**										
Beksinska et al (2015)^19^, ^20^, ^21^	Journal article; individually randomised crossover	Durban, South Africa; 2013	110 women	29 years (SD 6; range 18–45); heavy flow 46·2%	Mpower Mcup; (vaginal)	Usual product (ie, disposable pads, tampons, cloths)	6 cycles (3 cycles each product)	Acceptability and performance	4·5%	5
Hoffmann et al (2014)^22^	Journal article; cluster randomised	Jehanabad district, Bihar, India; 2012	960 women; 174 randomly assigned to cup group and 46 chose to use cup	29·9 years (SD 6·7); NR	NR	Usual product (ie, cloth and disposable pads)	8 months	Acceptability, demand for high-barrier menstrual cup and low-barrier sanitary pads	15·8% (6 months)	4
Howard et al (2011)^23^	Journal article; individually randomised	Vancouver, Canada; 2006–07	110 women; 56 in cup group	Range 19–40 years; heavy flow 11·1%	Divacup (vaginal)	Tampons	4 cycles	Use, use in future, costs, and waste	11·8%	3
Oster et al (2011),^24^ Oster et al (2012),^25^ Oster et al (2009)^26^, ^27^	Journal article; individually randomised	Bharatpur, Chitwan district, Nepal; 2006–08	199 schoolgirls; 98 in cup group	14·2 years (SD 1·2); NR	Mooncup (vaginal)	Usual product (ie, cloths and disposable pads)	15 months	School attendance, peer effect	0·5%	3
Phillips-Howard et al (2016),^5^ Nyothach et al (2015),^28^ Juma et al (2017),^29^ Mason et al (2015),^30^ Oduor et al (2015),^31^ van Eijk et al (2018)^32^	Journal article; cluster randomised	Gem district, Siaya Province, Kenya; 2012–13	766 schoolgirls; 229 in cup group	14·6 years (SD 0·7); heavy flow 20·8%	Mooncup (vaginal)	Disposable pads and usual practice (ie, cloths and pads)	Median 10·9 months	School drop-out, STIs, reproductive tract infections	15·7%	6
**Observational studies**										
APHRC (2010)^33^, ^34^, ^35^	Report; cohort study	Nairobi, Kenya; 2008	36 women and 60 schoolgirls	NR; NR	Mooncup (vaginal)	Disposable pads, cloths, cotton wool, tampons	3 cycles	Feasibility	6·3%	2
Averbach et al (2009)^36^	Journal article; survey and focus group discussions	Epworth, Zimbabwe; 2007–08	43 women	Range 18–45 years	Duet (cervical, re-usable)	Cotton wool, cloths, disposable pads, tissue	NA	Consideration of menstrual cup use	NA	ND
Borowski et al (2011)^37^	Master's thesis; survey	USA; 2011	155 women	Age ≥18 years; NR	No particular brand	NR	NA	Consideration of eco-friendly menstrual products	NA	ND
Care International in Uganda (2018)^38^	Report; cohort	Refugee settlement, Uganda; 2018	80 girls and women and 20 female trainers	n=25 15–18 years, n=41 19–25 years, n=34 26–30 years; NR	Ruby cup	Disposable and re-usable pads, cloths	3 months	Menstrual cup use	53·8%	2
Cattanach et al (1991),^39^Cattanach et al (1990)^40^†	Journal article; cohort	Hawthorn, Australia; NR	80 women	Range 17–42 years; NR	Gynaeseal (cervical)	NR	18 months	Acceptability	69·1%	2
Cheng et al (1995)^41^	Journal article; cohort	NR, Canada; 1991–92	51 women	46 (90%) of 51 <40 years; moderate to heavy flow: 42 (82%) of 51	Menses cup‡ (vaginal)	Tampons and disposable pads	2–13 cycles	Acceptability of menstrual cup for measuring flow	NR	2
Chintan et al (2017)^42^	Journal article; cohort	India (several sites); NR	100 women	Range 14–55 years; NR	Flow care (vaginal)	Disposable pads and tampons	8 weeks	Menstrual cup use	NR	2
Femme International (2017)^43^§	Report; cohort	Kilamanjaro region, Tanzania; 2016–17	184 adolescents and 38 women	Range 12–54 years; NR	Ruby cup (vaginal)	NR	6–12 months	Menstrual cup use	37–88%	2
Ganyaglo et al (2018),^44^ Ryan et al (2018)^45^	Journal article and abstract; repeated measures design	Ghana; 2016	11 women	43·6 years (SD 12·3); NR	Diva cup	Pads	4 h	Menstrual cup use for vesicovaginal fistula	0	5
Gleeson et al (1993)^46^	Journal article; cohort	Dublin, Ireland; NR	22 women	NR; 12 (55%) women had menorrhagia	Gynaeseal (cervical)	Tampons	1 cycle	Leakage, ease, use for measuring flow	0	3
Grose et al (2014)^47^	Journal article; survey	California, USA; NR	151 undergraduates	Range 18–23 years; NR	Brand not reported	NR	NA	Consideration of menstrual cup	NA	ND
Kakani et al (2017)^48^	Journal article; cohort	Dharpur, Gujarat, India; NR	158 women	31 years (SD 6·1; range 21–50); heavy flow: 20 (13%) of 150	NR: 44 mm diameter, thin walled silicon¶	Cloths, disposable pads, tampons	3 cycles	Acceptability and efficacy	5·1%	3
Madziyire et al (2018)^49^, ^50^§	Journal article; cohort	Epworth, Zimbabwe; 2016–17	54 women	Range 18–45 years; no information on heavy flow	Butterfly (vaginal)	NR	3 cycles; 1 year	Acceptability, leakage	3·7%	3
North et al (2011)^13^	Journal article; cohort	USA (7 centres); NR	406 women	Range 18–55 years; no information on heavy flow	Soft cup‖ (disposable cervical)	Disposable pads or tampons, or both	3 cycles	Safety, effectiveness, and acceptability	24·1%	3
Parker et al (1964)^51^	Journal article; cohort	Ann Arbor, USA; NR	65 women	NR; 46 women with menorrhagia, 19 with normal flow	Tassette (vaginal)	Tampons and disposable pads	2–6 months	Acceptability	15·2%	3
Pena et al (1962)^52^	Journal article; cohort	Florida, USA; NR	125 women (100 with normal flow and 25 with vaginal infections)	Range 20–45 years; all participants had normal flow	Tassette (vaginal)	Tampons and disposable pads	3 cycles	Not clear	NR	2
Shihata et al (2014)^53^	Journal article; cohort	Sweden, USA, Mexico, Brazil, Colombia; 2013	146 women	Range 18–40 years; NR	FemmyCycle (one size, vaginal)**	Disposable pads, tampons	3 cycles	Leakage, acceptability	28·1%	2
Stewart et al (2010)^54^	Journal article; cohort	Nottingham, UK; 2008–09	54 women	Mean 22·5 (SD NR); NR	Mooncup (vaginal)	Tampons and disposable pads	6 cycles (3 with cup)	Leakage, acceptability	61·1%	2
Stewart et al (2009)^55^	Journal article; survey	Nottingham, UK; NR	69 clinic patients	n=18 <30 years, n=21 30–40 years, n=30 >40 years; NR	Mooncup (vaginal)	Tampons and disposable pads	NA	Consideration of menstrual cup	NA	ND
Tellier et al (2012)^56^	Report; cohort study	Kitgum, Uganda; NR	31 women	24 years (SD NR); NR	Ruby cup (vaginal)	Cloths, gauze, disposable pads	3–5 cycles	Acceptability, safety	51·6%	3
Wiebe et al (2012)^57^	Journal article; retrospective chart survey	Vancouver, Canada; 2009	930 women; 96 used menstrual cups	75 (59%) of 96 <30 years; NR	No particular brand or type	NA	6 weeks	IUD expulsion within 6 weeks after placement, by menstrual product used	NA	ND
**Studies with only qualitative information**										
Hyttel et al (2017)^58^††	Journal article; two focus group discussions and six semi-structured interviews	Bungatira, Gulu, Uganda; 2013	36 schoolgirls (purposely selected)	14·6 years (SD 0·7; range 13–17); NA	Ruby cup (vaginal)	NA	4 months after study start	Willingness and ability to use	NA	Medium
Sundqvist et al (2015)^59^	Thesis; in-depth interviews	Msiriwa, Tanzania; 2014	15 schoolgirls	Range 14–15 years; NA	Lady cup (vaginal)	NA	NR	Effect of menstrual cup use on education and social interactions	NA	Strong
**Case reports**										
Adedokun et al (2017)^60^	Abstract; case report	Brno, Czech Republic; NR	1 woman	30 years; NA	NR	NA	NR	Hydronephrosis	NA	ND
Nunes-Carneiro et al (2018)^61^	Journal article; case report	Porto, Portugal; NR	1 woman	26 years; NA	NR	NA	5 days	Uretero-hydronephrosis	NA	ND
Stolz et al (2019)^62^	Journal article; case report	NR; NR	1 woman	47 years; NA	NR	NA	A “couple of weeks”	Hydronephrosis	NA	ND
Day et al (2012)^63^	Journal article; case report	London, UK; NR	1 woman	20 years; NA	Mooncup (vaginal)	NA	NR	Menstrual cup retention	NA	ND
FDA MAUDE database^14^	Results database search; case reports	USA; 1950–June, 2018	12 women	NR; NA	Mooncup, Diva cup, Femmy cycle, Softcup (vaginal and cervical)	NA	Variable	Adverse events (in table 2)	NA	ND
Seale et al (2019)^64^	Journal article; case series	Denver, CO, USA; NR	7 women	n=1 16 years: n=6 22–25 years; NA	NR	NA	2–12 months	IUD expulsion	NA	ND
Goldberg et al (2016)^65^	Journal article; case report	New Brunswick, Canada; 2013	1 woman	39 years; NA	NR (vaginal)	NA	NR	Use as diagnostic aid of vesicouterine fistula	NA	ND
Mitchell et al (2015)^66^	Journal article; case report	Ontario, Canada; NR	1 woman	37 years; NA	DivaCup (vaginal)	NA	2 weeks post-admission	Possible TSS	NA	ND
Russell et al (2016)^67^	Journal article; case reports	Utah, USA; NR	3 women	54 years, 60 years, and 68 years; NA	NR (vaginal)	NA	NR	Use as enterovaginal or vesicovaginal fistula control	NA	ND
Spechler et al (2003)^68^	Journal article; case report	Bethesda, MD, USA; NR	1 woman	41 years; NA	Keeper (vaginal)	NA	2 years post-surgery	Adenomyosis and endometriosis	NA	ND
**Other types of study with relevant information**										
Cattanach et al (1989)^69^†	Journal article; vaginal samples	Hawthorn, Australia; 1986–88	5 women	Range 19–32 years; NA	Gynaeseal* (cervical)	NA	3–22 months	Menstrual cup safety, effect on vaginal flora	NA	ND
Karnaky et al (1962)^70^	Journal article; vaginal observations and samples	Houston, TX, USA; NR	Two cohorts of 50 and 97 women; and a survey of 20 women	NR; NA	Tassette (vaginal)	NA	Unclear for cohort studies	Menstrual cup safety, effects on vagina	NA	ND
Tierno et al (1989)^71^	Journal article; in-vitro study	New York, NY, USA; NR	NA	NA; NA	16 menstrual cups, brands not reported	NA	NA	Ability to induce TSST-1 production by TSS-associated strains of Staphylococcus	NA	ND
Tierno et al (1994)^72^	Journal article; in-vitro study	New York, NY, USA; NR	NA	NA; NA	Six Tassaway cups (vaginal)	NA	NA	Ability to induce TSST-1 by a TSS strain of *Staphylococcus aureus* MN8	NA	ND
Nonfoux et al (2018)^73^	Journal article; in-vitro study	France; NR	NA	NA; NA	2 be'Cup and 2 MeLuna (vaginal)	NA	NA	Effect on *S aureus* growth and TSST-1 production using the modified sac method	NA	ND

Where data are missing, it was not provided in the source material. Cycles refer to menstrual cycles. A quality score of 5–6 indicates a moderate-to-high quality study, and a score of less than 5 indicates a medium-to-low quality study. For qualitative studies, levels of study quality were strong, medium, and weak. The quality score components of individual studies are in the appendix (pp 7, 8, 10). Cloths=pieces of material (clothing, blankets, socks) that are used for menstruation and can be reused after washing or disposed of after use. NR=not reported. STI=sexually transmitted infection. APHRC=African Population and Health Research Center. NA=not applicable. ND=not done (these studies were not assessed for quality). IUD=intrauterine device. FDA=US Food and Drug Administration. MAUDE=Manufacturer and User Facility Device Experience. TSS=toxic shock syndrome. TSST-1=toxic shock syndrome toxin 1.

* Manufacturing company, city, country, and website where available, are listed in the appendix (p 8).

† The study author was the developer of Gynaeseal (a disposable cup covering the cervix. that can also be worn during intercourse); we assumed the articles from 1990 and 1991 described the same study and used the publication with the larger sample size (1991).

‡ This type of cup has a drainage tube that can be opened to let menstrual fluids pass.

§ Additional information obtained from internal report or author.

¶ Description in article is like a cervix-covering cup (“The device- the menstrual cup we utilized for the study is an internally worn device with a pliable rim 44mm in diameter and a thin-walled reservoir to collect and hold the menstrual fluid. It was designed to minimize bulk in order to facilitate insertion and removal. Once inserted; it opens to an oval shape, positioned between the posterior fornix and the notch behind the pubic bone, covering the cervix. Removal is accomplished by hooking a finger over the rim behind the pubic bone. It is made up of health grade non-toxic non- allergic silicon”), but image is of a low vaginal cup.

‖ Instead Softcup (a rebrand of Softcup): disposable cup covering the cervix. This type of cup can also be worn during intercourse.

** Author has patent on this menstrual cup.

†† Part of a larger study (Gulu Schoolgirl Menstrual Cup Study, n=194), for which no other publication could be retrieved.

**Figure 2:**
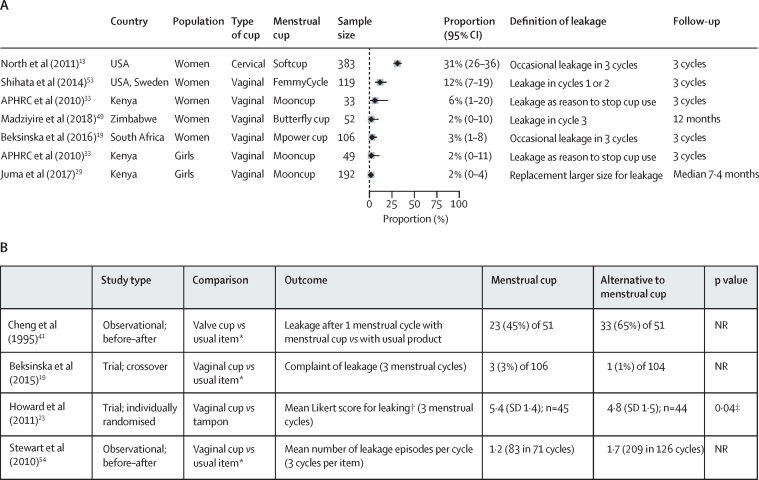
Menstrual cup and leakage (A) Proportion of participants who had menstrual leakage in seven studies using different types of menstrual cups and definitions. (B) Reports of leakage among menstrual cup users versus users of other menstrual products. APHRC=African Population and Health Research Center. NR=not reported. *Disposable pad or tampon. †Likert scale: 7-point score, in which 1=terrible and 7=great. ‡p value reported in article for Mann-Whitney test.

With regard to leakage, only four studies (n=293) made direct comparisons between menstrual cups and usual products. The outcomes in each of these studies were different, but leakage between products was similar in three studies and significantly less among menstrual cups for one study (figure 2).^23^ In studies that assessed menstrual cups that are still available, the proportion of leakage among the participants who reported use of the menstrual cup was 2–31% for a wide range of definitions, as shown in figure 2. Some factors mentioned in association with leakage by study authors included menorrhagia,^48^ unusual anatomy of the uterus,^53^ need for a larger size of menstrual cup,^5^ and incorrect placement of the menstrual cup, or that it had filled to capacity.^39^, ^53^

When looking at safety, use of the menstrual cup was not associated with abnormalities in the vagina or cervix in three studies with vaginal examinations (n=370; table 2).^13^, ^52^, ^70^ Three users reported vaginal wounds in case reports, which could not be confirmed with medical records. In one case report, severe pain on removal was self-reported and in another case report severe pain was self-reported when wearing the menstrual cup,^14^ and two participants in two different cohort studies reported vaginal or cervical irritation without clinical consequences.^39^, ^41^ Three adverse events that were reported in one cohort study and three case reports were possibly related to an allergy; one case of silicone allergy necessitated reconstructive vaginal surgery.^13^, ^14^, ^48^ Difficulty with removal that required professional assistance—an adverse event we did not anticipate—was reported 47 times for cervical cups (one participant from a cohort study, and 46 case reports) and twice for vaginal cups (both case reports).^13^, ^14^, ^46^, ^63^

**Table 2:** Safety and side-effects of the menstrual cup

		**n (%) or description**	**Notes**	**Data source**
**Handling and positioning of menstrual cup**				
Vaginal wound				
	Cup not clear (Divacup or softcup)	Event April, 2012; vaginal wound due to use of menstrual cup, needing treatment from physician for vaginal bleeding	Complete medical records were not available for evaluation	FDA database^14^
	Softcup (cervical)	Reported April, 2012; long-term customer of softcup product claimed vaginal scarring due to use	Medical director did not find anything in medical records provided by customer related to vaginal health	FDA database^14^
	Softcup (cervical)	FDA database case report: “…cup wore through the vaginal wall, damaging an artery that required surgical repair”	Event could not be confirmed; no medical records were available	North et al (2011)^13^
Vaginal pain on removal				
	Divacup (vaginal)	Event March, 2017; extreme pain on removal (first use), individual stopped using the cup	Self-report; no medical report available	FDA database^14^
Pelvic pain				
	Softcup (cervical)	Event February, 2017; pain in lower pelvis and rectum and nausea about 1 h after insertion, no longer present approximately 30 min after removal	Self-report; no medical evaluation available; individual stopped use after trying twice (possibly vascular compression)	FDA database^14^
Vaginal irritation				
	Gynaeseal (cervical)	One (1%) of 73	Self-report by participant	Cattanach et al (1991)^39^
	Cervix irritation			
	Menses cup (vaginal)	One (2%) of 51	Cervical smear was normal	Cheng et al (1995)^41^
Allergy and rash				
	NR, vaginal cup	Allergy: one (1%) of 150; and rash: two (1%) of 150	· ·	Kakani et al (2017)^48^
	Softcup (cervical)	FDA database: two case reports	NR	North et al (2011)^13^
	Mooncup (vaginal)	Event 2010: silicone allergy in one individual	Surgery was needed for vaginal repair; manufacturer noted that silicone allergy is very rare	FDA database^14^
Difficulty with removal requiring professional assistance				
	Gynaeseal (cervical)	One (5%) of 22	· ·	Gleeson et al (1993)^46^
	Softcup (cervical)	FDA database: three case reports reported by North 2011; one event in 2018	· ·	North et al (2011),^13^ FDA database^14^
	Softcup (cervical)	Reported complaints to company 2003–08: 42 individuals underwent physician-assisted removal	Other complaints reported to company included poor fit (n=102), leakage (n=168), messy (n=98)	North et al (2011)^13^
	Mooncup (vaginal)	Case report: menstrual cup lodged on cervix, difficult to remove, requiring assistance	Moderate cervical inflammation after retrieval	Day et al (2012)^63^
	Divacup (vaginal)	Event April, 2015: one case report required an emergency room visit for removal	· ·	FDA database^14^
**Reproductive tract observations with use of menstrual cup**				
Vulva abnormalities				
	Softcup (cervical)	Baseline: four (1%) of 393; cycle 1: eight (2%) of 365; cycle 2: six (2%) of 326; cycle 3: five (2%) of 305	Vulva-vaginal inspection at baseline and monthly for 3 months; no p values reported	North et al (2011)^13^
Abnormalities of vaginal wall				
	Softcup (cervical)	Zero of 44	Vulva-vaginal inspection at baseline and monthly for 3 months	North et al (2011)^13^
	Tassette (vaginal)	Zero of 12	Vaginal inspection after 3 months	Pena et al (1962)^52^
	Tassette (vaginal)	Zero of 50	Vaginal inspection done; timing of inspections not clear	Karnaky et al (1962)^70^
Abnormalities of cervix				
	Softcup (cervical)	Baseline: 23 (6%) of 390; cycle 1: ten (3%) of 345; cycle 2: six (2%) of 326; cycle 3: four (1%) of 300	Inspection of cervix; no p values reported for differences	North et al (2011)^13^
	Softcup (cervical)	Abnormal cervical smear test: baseline: one (<1%) of 406; cycle 1: one (<1%) of 368; cycle 2: two (1%) of 329; cycle 3: zero of 308	Abnormal cervical smear test results were exclusion criteria at admission, and a reason for discontinuation of the study; no p values reported for differences	North et al (2011)^13^
Condition of vaginal and cervical epithelium				
	Softcup (cervical)	44 women examined at baseline, 37 at 2–3 months, and 25 at 5–6 months	“The Softcup caused no alteration or disruption in vaginal or cervical epithelium, as assessed by colposcopy and cervical cytology”	North et al (2011)^13^
**Vaginal flora and infections with use of menstrual cup**				
pH changes of vagina				
	Tassette (vaginal)	Zero of 50	No abnormalities, vaginal areas where menstrual cup was placed were more acid	Karnaky et al (1962)^70^
	Softcup (cervical)	Mean pH at baseline: 4·6 (n=400); cycle 1: 4·6 (n=368); cycle 2: 4·6 (n=329); cycle 3: 4·5 (n=308)	No p values reported	North et al (2011)^13^
Clue cells (vaginal smear) Lactobaccilus				
	Softcup (cervical)	Number with clue cells: baseline n=6; cycle 1 n=6; cycle 2 n=2; cycle 3 n=4	Sample sizes and p values were not reported	North et al (2011)^13^
Lactobaccilus				
	Softcup (cervical)	“…before, during, and after use of the cup, vaginal Lactobacillus (normal vaginal flora) was maintained at normal levels.”	Data in figure 3 in publication cannot be extracted; no significant changes according to authors	North et al (2011)^13^
Gardnerella vaginalis				
	Softcup (cervical)	No significant changes from baseline-cycle 3 according to authors	Data in figure 3 in publication cannot be extracted	North et al (2011)^13^
Bacterial vaginosis				
	Softcup (cervical)	No significant changes from baseline to cycle 3 according to authors	Data in figure 3 in publication cannot be extracted	North et al (2011)^13^
	Mooncup (vaginal)	Study end survey: cup 21 (15%) of 144; pads 40 (20%) of 202, and usual practice (control) 32 (21%) of 156; cup *vs* control p=0·11 and cup *vs* pads p=0·13; among girls enrolled for ≥9 months: cup 13 (13%) of 101, pads 29 (20%) of 143, usual practice 20 (19%) of 104; cup *vs* control p=0·07, and cup *vs* pads p=0·018	Cluster randomised trial of schools; median follow-up 11 months (range 3–15)	Phillips-Howard et al (2016)^5^
Candidiasis				
	Softcup (cervical)	Number with candidiasis: baseline n=6; cycle 1 n=6; cycle 2 n=3; cycle 3 n=6	Sample sizes not reported; according to authors, yeast decreased significantly from month 1 to 2	North et al (2011)^13^
	Ruby cup (vaginal)	Zero of 18 participants had vaginal candidiasis at follow-up (3–5 months)	NA	Tellier et al (2012)^56^
	Tassette (vaginal)	*Candida albicans* decreased with the use of the cup	NR	Karnaky et al (1962)^70^
	Mooncup (vaginal)	Study end survey: cup 11 (8%) of 143, pads 19 (10%) of 200, usual practice (control) 13 (9%) of 156; cup *vs* control p=0·87, and cup *vs* pads p=0·68	Cluster randomised trial of schools; median follow-up 11 months (range 3–15)	Phillips-Howard et al (2016)^5^
Group B Streptococcus				
	Softcup (cervical)	No differences between baseline and cycle 1 to cycle 3	Data in figure 3 in publication cannot be extracted; no significant changes according to authors	North et al (2011)^13^
Enterococcus				
	Softcup (cervical)	Increase in *Enterococcus* from cycle 2 to cycle 3 (p=0·03)	“… this increased frequency persisted for 3 months after discontinuing use of the cup, suggesting that factors or behavior other than cup use may have influenced colonization”; data in figure 3 in publication cannot be extracted	North et al (2011)^13^
E coli				
	Softcup (cervical)	No significant changes from baseline to cycle 3 according to authors	Data in figure 3 in publication cannot be extracted	North et al (2011)^13^
Escherichia coli on menstrual cup				
	Mooncup (vaginal)	Nine (53%) of 17 if used cup for <6 months; four (22%) of 18 if used for ≥6 months (p=0·12); association between *E coli* with heavy periods: 61·5% of girls reporting heavy periods had *E coli* on cups, compared with 22·7% of those stating they did not have heavy periods (p=0·022, no numbers presented)	Cluster randomised trial of schools; median follow-up 11 months (range 3–15)	Juma et al (2017)^29^
Chlamydia trachomatis				
	Mooncup (vaginal)	Study end survey: cup three (2%) of 144, pads three (2%) of 201, usual practice (control) seven (5%) of 154; cup *vs* control p=0·20, and cup *vs* pads p=0·63	Cluster randomised trial of schools; median follow-up 11 months (range 3–15)	Phillips-Howard et al (2016)^5^
Trichomonas vaginalis				
	Softcup (cervical)	Zero cases at baseline, and cycles 1 to 3	Sample sizes not reported	North et al (2011)^13^
	Mooncup (vaginal)	Study end survey: cup two (1%) of 143, pads five (3%) of 200, usual practice (control) seven (5%) of 154; cup *vs* control p=0·12, and cup *vs* pads p=0·36	Cluster randomised trial of schools; median follow-up 11 months (range 3–15)	Phillips-Howard et al (2016)^5^
	Ruby cup (vaginal)	Zero of 18 at baseline, and at 3–5 months of follow-up	NA	Tellier et al (2012)^56^
Neisseria gonorrhoea				
	Mooncup (vaginal)	Study end survey: cup one (1%) of 144, pads one (1%) of 201, usual practice (control) one (1%) of 154; cup *vs* control p=0·96, and cup *vs* pads p=0·81	Cluster randomised trial of schools; median follow-up 11 months (range 3–15)	Phillips-Howard et al (2016)^5^
	Ruby cup (vaginal)	Zero of 18 at baseline, and at 3–5 months of follow-up	NA	Tellier et al (2012)^56^
Staphylococcus aureus				
	Softcup (cervical)	No significant changes in cycles 1–3 compared with baseline	Data in figure 3 of publication cannot be extracted	North et al (2011)^13^
	Mooncup (vaginal)	Among menstrual cup users: four (11%) of 38 in first month of intervention, 13 (9%) of 139 after first month; p=0·83 (median follow-up 4 months, range 2–11 for this substudy); prevalence was 21 (11%) of 197 in sanitary pads group, and 16 (11%) of 153 in usual practice group	Cluster randomised trial in schools; median follow-up 11 months (range 3–15); samples from vaginal swab (self-swabbing)	Juma et al (2017)^29^
	be'Cup (vaginal)	Silicone cup: potentially more *S aureus* after incubation for 8 h with shaking in a plastic bag with *S aureus* in one of two cups used, but not when no shaking	In-vitro study	Nonfoux et al (2018)^73^
	Me Luna (vaginal)	Thermoplastic isomer cup: no more *S aureus* after incubation for 8 h with shaking in plastic sac, and not when no shaking	In-vitro study	Nonfoux et al (2018)^73^
TSST-1				
	Mooncup (vaginal)	49 schoolgirls with vaginal *S aureus* had second swab: ten yielded *S aureus*, two had TSST-1, both in sanitary pad group; the cases were asymptomatic	Cluster randomised trial in schools; median follow-up 11 months (range 3–15); sample from vaginal swab (self-swabbing)	Juma et al (2017)^29^
	NR	No TSST-1 in supernatant of *S aureus* cultivated for 24 h (incubated aerobically in a still growth environment) in the presence of elastic polymer menstrual cup (n=16 menstrual cups)	In-vitro study	Tierno et al (1989)^71^
	Tassaway (vaginal)	*S aureus* MN8 produced no TSST-1 when grown in the presence of Tassaway (elastomeric polymer, n=6), washed or unwashed, no shaking, incubation overnight	In-vitro study	Tierno at al (1994)^72^
	be'Cup (vaginal)	Silicone cup: potentially more TSST-1 production after incubation for 8 h with shaking in plastic bag with *S aureus* compared with control, but not when not shaken or with pieces of cup	In-vitro study	Nonfoux et al (2018)^73^
	Me Luna (vaginal)	Thermoplastic isomer cup: potentially more TSST-1 production after incubation for 8 h with shaking in plastic bag with *S aureus* compared with control, but not when not shaken or with pieces of cup	In-vitro study	Nonfoux et al (2018)^73^
TSS				
	Mooncup (vaginal)	Zero of 192 in trial in Kenya	“Safety monitoring components comprised routine nurse-based screening, population-based monitoring (school and community) and clinical evaluation of infection with laboratory confirmation”	Juma et al (2017)^29^
	Softcup (cervical)	Two case reports in the FDA database	Both unconfirmed cases of TSS	North et al (2011)^13^
	Divacup (vaginal)	One case report: blood cultures and urine culture negative, no culture of the menstrual cup was done	Woman had history of Hashimoto's thyroiditis and chronic menorrhagia	Mitchell et al (2015)^66^
	Mooncup (vaginal)	Event February, 2012: TSS 2 days after using of first and new Mooncup resulting in 9 days of inpatient hospital stay; vaginal swab positive for *S aureus*	Had an IUD, Mooncup was not sent for bacteriological testing	FDA database^14^
	Divacup (vaginal)	Event February, 2015: TSS from Streptococcus resulting in 5 days of i-patient hospital stay; culture of cup isolated group A and B *streptococcus*	Woman had used Divacup for menstrual period, which started 3 days before illness; menstrual cup was in for 18 h on admission to hospital	FDA database^14^
UTI				
	Ruby cup (vaginal)	Baseline: four (13%) of 31; at follow-up (after 3–5 months) three (17%) of 18; p=0·65, McNemar test	One participant with a UTI at enrolment and follow-up had her cup stolen and used toilet paper in vagina as a tampon	Tellier et al (2012)^56^
	Gynaeseal (cervical)	One (1%) of 73 had transient dysuria	“The woman who developed dysuria did not seek treatment and the problem subsided within 24–48 hours”	Cattanach et al (1991)^39^
	Softcup (cervical)	Urine analysis done; detailed results not reported	“Monthly monitoring of gynecological health via urinalysis, pelvic examination with visual evaluation of tissues, vaginal pH, and microscopic wet mount showed no adverse effects of cup use”	North et al (2011)^13^
	Softcup (cervical)	Event August, 2014: UTI confirmed by urine cultures twice after use of softcup	Medical records were not available for evaluation	FDA database^14^
Infections overall				
	Tassette (vaginal)	“The amount of bacterial contamination was greatest with the pad, next with the tampon and least with the rubber cup”	No data provided; study reported to make cultures from vaginal wall samples and to examine fresh and stained smears for *C albicans, Trichomonas vaginalis, Haemophilus vaginalis*, and for predominance of Gram-positive or Gram-negative cocci, small rods or long-rod bacilli (*Doederlein bacilli*)	Karnaky et al (1962)^70^
	Softcup (cervical)	FDA database: one case report	Vaginal infection not further specified; could not be confirmed at follow-up	North et al (2011)^13^
	Butterfly cup (vaginal)	“…none of the women sought treatment for a pelvic infection. There was no onset or worsening of dysmenorrhoea in 83%, dyspaurenia in 94%, pelvic pain in 92% and vaginal discharge in 92% of the participants during the 12 months of cup use”; n=52	NA	Madziyire et al (2018)^49^, ^50^
	Gynaeseal	“There was no increased pathogenicity detected in vaginal flora. There was a trend towards smaller numbers of potentially pathogenic bacteria for 4 of the women, and the remaining woman showed no change. None of the women developed any significant medical problems”	Vaginal swabs before and after use, five women, median follow-up 14 months (range 3–22)	Cattanach et al (1989)^69^
STIs				
	Mooncup (vaginal)	Study end survey: menstrual cup six (4%) of 144, pads nine (5%) of 202, and usual practices (control) 12 (8%) of 156; cup *vs* control p=0·11, and cup *vs* pads p=0·87; when follow-up was ≥9 months: cup four (40%) of 101, pads seven (5%) of 143, and usual practice 11 (11%) of 104; cup *vs* control p=0·004, and cup *vs* pads p=0·60	Presence of either *C trachomatis, T vaginalis* or *N gonorrhoea*; cluster randomised trial of schools in Kenya; median follow-up 11 months (range 3–15)*	Phillips-Howard et al (2016)^5^
Reproductive tract infections				
	Mooncup (vaginal)	Study end survey: cup 31 (22%) of 144, pads 58 (29%) of 202, and usual practice (control) 42 (27%) of 156; cup *vs* control p=0·36, and cup *vs* pads p=0·19	Presence of either *B vaginosis* or *C albicans;* cluster randomised trial of schools in Kenya; median follow-up 11 months (range 3–15)	Phillips-Howard et al (2016)^5^
**Other adverse events**				
Urinary incontinence				
	Femcap (first model of femmycycle, vaginal)	FDA database: one case report; event July, 2014; pelvic pain and urinary incontinence when wearing and removing menstrual cup; urine sample negative for infection	Self-report; stopped using menstrual cup	FDA database^14^
Displacement of IUD when using menstrual cup				
	NR	IUD expulsion 6–8 weeks after insertion: menstrual cup five (4%) of 135, tampon 11 (2%) of 469, pads: seven (4%) of 169; cup *vs* tampon p=0·57, and cup *vs* pads: p=0·92	Retrospective cohort; expulsion of an IUD occurs in approximately one in 20 women and is most common in the first 3 months after insertion; expulsion commonly occurs during menstruation; some recommend not to use internal sanitary protection for 3–6 weeks after insertion because of an increased infection risk	Wiebe et al (2012)^57^
	Mooncup (vaginal)	FDA database: one case report; event July, 2012; potential IUD dislodgment after Mooncup removal; patient had an ectopic pregnancy and needed surgery	Patient felt pain after removal of Mooncup and had the position of the IUD checked at a health centre where it was declared in position; 2 months later she was found to be pregnant	FDA database^14^
	NR	Case series of seven women with IUD expulsion when removing menstrual cup; expulsion occurred 1 week to 13 months after insertion of IUD and was recurrent in two women; of seven women, two choose to use different contraception; the five others had their IUD re-inserted	Two women opted for cutting the wires of the IUD close to the cervix to avoid the problem; authors also stress importance of releasing vacuum of menstrual cup before removal	Seale et al (2019)^64^
Endometriosis because of menstrual backflow via use of menstrual cup				
	Tassette (vaginal)	Position of cup confirmed with X-ray imaging	“Hence the free space available in the upper vagina plus the capacity of the cup itself are ample to accommodate several times the amount of blood passed in a complete menstrual cycle”	Pena et al (1962)^52^
	Tassette (vaginal)	No evidence for backflow	“Thin watery solutions could not be introduced under high pressures during the menstrual flow in 6 multiparous women”	Karnaky et al (1962)^70^
	Keeper (vaginal)	Case report: dysmenorrhoea 2 years after start of menstrual cup use (10 years ago tubal ligation); laparoscopy showed adenomyosis and endometriosis, treated with laser; patient stopped use of menstrual cup; pain decreased after surgery; 2 years of follow-up	“The observations in our patient suggest that it may be useful to inquire about use of these devices in women with pelvic pain or endometriosis”; petition for revoking of market approval to US FDA rejected because of lack of evidence^74^	Spechler et al (2003)^68^
Hydronephrosis (ie, renal colic)				
	NR	Case report: severe colicky flank pain; CT scan showed menstrual cup was slightly dislocated, pressing into left ureter	“The extraction of the menstrual cup resulted in resolution of hydronephrosis and associated symptoms”	Adedokun et al (2017)^60^
	NR	Case report: 3 h of back pain on the right side; low-dose unenhanced CT scan showed entrapment of left vaginal wall and part of interolateral bladder wall; improperly positioned menstrual cup	Symptoms and swelling disappeared after removal of menstrual cup, confirmed by another CT scan; patient had used a menstrual cup for a long time with no previous problems, and continued use of cup; no problems at follow-up after several weeks	Stolz et al (2019)^62^
	NR	Case report: 3 h of pain in the right flank, and nausea during menstruation; X-ray imaging showed menstrual cup orientated to the right	“The symptoms and the ureterohydronephrosis relieved completely after the removal of the device”; patient had used a menstrual cup for 2 years	Nunes-Carneiro et al (2018)^61^

Entries in FDA database^14^ for softcup not entered if before 2011, to avoid double reporting with North et al (2011).^13^ NR=not reported. NA=not applicable. TSST-1=toxic shock syndrome toxin-1. TSS=toxic shock syndrome. FDA=US Food and Drug Administration. IUD=intrauterine device. UTI=urinary tract infection. STI=sexually transmitted infection.

* The decrease in STIs in the trial in Kenya in the groups in which either menstrual cups or sanitary pads were provided is thought to be an indirect effect because of the decrease in risky sexual behaviour to obtain money to buy pads.

We found no increased infection risk (reproductive tract or systemic infection) associated with use of a menstrual cup among European,^54^, ^55^ North American, and African women and girls,^5^, ^19^ compared with other menstrual products (table 2). A decrease in candidiasis was reported with use of the menstrual cup in two of four studies that investigated this infection; one study found no candidiasis infections at follow-up in 18 participants, and the other, a randomised feasibility pilot among schoolgirls (aged 14–16 years) in Kenya comparing menstrual cups, sanitary pads, and usual practice (cloths, pads, tissue, or other makeshift materials), showed no difference in the prevalence of candidiasis by study group (menstrual cup 11 [8%] of 143, pads 19 [10%] of 200, and usual practice 13 [9%] of 156; menstrual cup *vs* pads p=0·68 and menstrual cup *vs* usual practice p=0·87; table 2).^5^, ^13^, ^56^, ^70^ One study^70^ reported lower prevalence of bacterial infections among users of the menstrual cup than among users of tampons or pads (not further specified), and a randomised pilot study^5^ in Kenya reported lower prevalence of bacterial vaginosis among users of the menstrual cup than users of pads and usual practice enrolled for 9 months or longer (menstrual cup 13 [13%] of 101, pads 29 [20%] of 143, usual practice 20 [19%] of 104; menstrual cup *vs* pads p=0·018 and menstrual cup *vs* usual practice p=0·074; table 2).^5^, ^70^ Toxic shock syndrome was identified in five case reports;^13^, ^14^, ^66^ microbiological confirmation was available with cultures from menstrual cup and blood showing streptococcus for one case.^14^ In two participants from two case reports, concomitant conditions were present (intrauterine device [IUD] in situ; an immunodeficiency disease).^14^, ^66^ A potential additional case of toxic shock syndrome was identified in a web blog (appendix pp 43–44): we could not determine whether this case has separately been reported in the MAUDE system or in a case report and thus it has been left out of our analysis. The prevalence of vaginal *Staphylococcus aureus* was examined among Kenyan schoolgirls participating in a randomised pilot study;^5^, ^29^ no difference was seen between menstrual cup, pads, and usual practice groups.^29^ No expression of toxic shock syndrome toxin 1 (TSST-1) was found in *S aureus* positive samples from menstrual cup users in this study.^29^ In-vitro studies of production of TSST-1 in the presence of menstrual cup material showed conflicting results (table 2).^72^, ^73^

An initial case report of a menstrual cup user about dislodgement of her IUD during use of a menstrual cup was followed by a case series of seven women who reported dislodgement of an IUD during removal of the menstrual cup between 1 week and 13 months of IUD insertion.^14^, ^64^ A retrospective chart survey did not find an increased risk for IUD expulsion within 6–8 weeks after insertion among menstrual cup users (five [4%] of 135), compared with tampons users (11 [2%] of 469) or pad users (seven [4%] of 169).^57^

One case-report^68^ suggested use of a menstrual cup might have been associated with the development of endometriosis;^68^ however, this hypothesis was not considered plausible by the regulatory authority and we did not identify any further reports on this possible association. We found three case reports^60^, ^61^, ^62^ of hydronephrosis and one^14^ of incontinence when using the menstrual cup; however, symptoms disappeared after menstrual cup removal (table 2).^14^, ^60^, ^61^ Other uses of menstrual cups—eg, as a contraceptive or temporary fistula control—are in the appendix (p 9)

**Figure 3:**
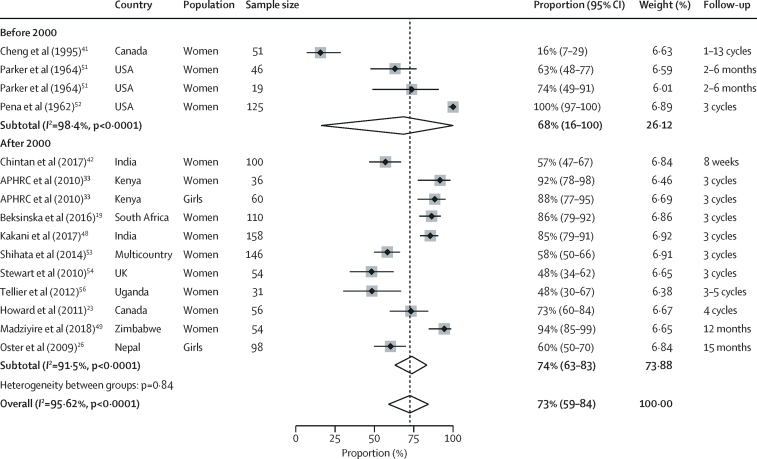
Proportion of women who wanted to continue menstrual cup use after the study All studies herein used vaginal cups. In Cheng et al (1995),^41^ a cup with a valve in the stem was used. In Parker at al (1964),^51^ one study population had menorrhagia (n=46), and the other population had normal flow (n=19). APHRC=African Population and Health Research Center.

When assessing uptake and acceptability, all six relevant qualitative studies were from low-income and middle-income countries (appendix pp 10–13),^30^, ^33^, ^34^, ^38^, ^56^, ^58^, ^59^ whereas 20 studies with quantitative information on uptake and acceptability were from low-income and middle-income countries and high-income countries (appendix pp 14–22).^13^, ^19^, ^22^, ^23^, ^24^, ^32^, ^33^, ^38^, ^39^, ^41^, ^42^, ^43^, ^46^, ^48^, ^49^, ^51^, ^52^, ^53^, ^54^, ^56^ In low-income and middle-income countries, usual products for menstruation included cloths, disposable pads, cotton wool, tissue paper, or other items, and leakage and chaffing is a common concern.^30^, ^33^, ^34^, ^58^, ^75^ All studies that assessed use of menstrual cups used some form of education and training on the menstrual cup. Girls and women expressed initial concerns in qualitative studies, noting the size of the menstrual cup.^30^ Many were concerned it could cause pain (and noted it often did so at first) or worried about reproductive harms (eg, infertility). In quantitative studies, 3% (pooled estimate: n=1251, 95% CI 1–6%, 11 studies; *I*^2^=79·3%) of participants reported they could not insert the menstrual cup and 11% (n=1190, 95% CI 3–23%, ten studies; *I*^2^=96·4%) reported discontinuation related to the menstrual cup (table 3). Pain on insertion was reported for ten (9%) of 106 menstrual cup users versus none of 104 using their usual method at 3 months of follow-up in a South African crossover trial (p value not reported).^19^ Initial discomfort on insertion was reported by 20% of participants (pooled estimate: n=1061, 95% CI 12–30%, 17 studies; *I*^2^=92·3%). All qualitative studies described user familiarisation with the menstrual cup over time, with practice, peer support, and training being key to success.^30^, ^33^, ^34^, ^38^, ^58^, ^59^ Longitudinal quantitative studies in low-income and middle-income countries showed a learning curve of 2–5 months (appendix p 22); colour change of the menstrual cup as an objective measure suggested use increased throughout the first year among Kenyan schoolgirls.^32^ A Nepalese study^25^ noted that self-reported increased use 2 months after distribution was associated with the presence of friends who successfully used the menstrual cup. In India^22^ and Tanzania,^43^ the uptake of menstrual cups was significantly slower than uptake of pads (appendix p 24).^22^, ^43^ In 15 studies with relevant information, 73% (pooled estimate: n=1144, 95% CI 59–84; *I*^2^=96%) of participants reported willingness to continue use of the menstrual cup after the study (figure 3). All qualitative and some quantitative studies reported a positive effect of use of the menstrual cup on participants' lives, decreased stress concerning staining and leakage, and improvements in mobility.^13^, ^51^, ^53^ Challenges described included difficulties with cleaning and storage of the menstrual cup in low-income and middle-income countries.^34^, ^58^ Other challenges were associated with emptying the menstrual cup in school or public toilets,^28^, ^34^, ^58^ which was also reported by participants in high-income countries.^37^ Menstrual cups were associated with a decrease in the average number of changes per cycle in a UK study compared with tampons or sanitary pads.^54^ Three qualitative studies implied that school attendance, concentration, and performance improved after participants were given a menstrual cup.^30^, ^34^, ^58^ No measured difference in school absence or test results between products were reported (appendix p 24).^5^, ^27^, ^34^ A study in Nepal noted a significant decrease in time spent doing laundry for menstrual cup users compared with those using usual practice.^27^

**Table 3:** Pooled estimates of meta-analyses for different outcomes of acceptability

		**Pooled prevalence**	**Number of studies (or subgroups)**	**Total participants**	I^2^	**p**_heterogeneity_	p value for Z test*
Could not insert cup		2·8% (0·8–5·6)	11	1251	79·3%	<0·0001	0·0002
Used menstrual cup at least once (verbal report)		79·3% (68·5–88·4)	25	2367	97·1%	<0·0001	<0·0001
Menstrual cup-related discontinuation		10·7% (2·7–22·6)	10	1190	96·4%	<0·0001	0·0004
Discontinuations for other reasons		9·0% (3·8–15·9)	15	1783	94·9%	<0·0001	<0·0001
Difficult to insert (among users)		20·3% (11·7–30·4)	17	1061	92·3%	<0·0001	<0·0001
	First cycle	35·3% (15·4–58·1)	5	272	92·7%	<0·0001	<0·0001
	Later cycles†	13·0% (8·1–18·7)	12	789	74·3%	<0·0001	<0·0001
Uncomfortable to wear		12·6% (5·9–21·3)	12	958	91·9%	<0·0001	<0·0001
	First cycle	32·9% (2·2–76·2)	3	221	97·5%	<0·0001	0·0148
	Later cycles‡	7·9% (4·0–12·9)	9	737	77·1%	<0·0001	<0·0001
Difficulty removing		9·3% (2·9–18·3)	7	461	84·7%	<0·0001	0·0001
Continue using the cup		72·5% (59·0–84·3)	15	1144	95·6%	<0·0001	<0·0001
	Study before 2000	68·5% (16·1–100)	4	241	98·4%	<0·0001	0·0014
	Study after 2000§	73·8% (63·0–83·3)	11	903	91·5%	<0·0001	0·0001

Data in parentheses are 95% CIs. No significant difference was found for any of the subgroup analyses (high-income *vs* low-income and middle-income countries, adult women *vs* adolescents, type of cup, duration of follow-up, or high *vs* low or moderate study quality).

* A significant Z test indicates that the pooled prevalence is different from zero.

† Difficult to insert: first *v*s later menstrual cycles: p=0·15.

‡ Uncomfortable to wear: first *vs* later menstrual cycles: p=0·13.

§ Continue to use cup: before *vs* after 2000: p=0·29.

An economic advantage of a menstrual cup emerged in qualitative studies, with participants (and families) citing monthly cost savings from not needing to purchase pads or soap for laundry. Two qualitative studies included quotes from participants showing that menstrual cups might decrease the need for transactional sex to purchase pads.^34^, ^38^ This finding might be corroborated by results from a randomised controlled study among schoolgirls (aged 14–16 years) in rural western Kenya that noted a significantly lower prevalence of sexually transmitted infections among participants who were provided by the study with either menstrual cups or disposable pads versus controls (ie, using usual practice), citing lower exposure to transactional sex as a probable reason (table 2; appendix p 24).^5^

A study in schoolgirls in Kenya (aged 14–16 years) in an area with poor water and sanitation^76^ reported dropping of menstrual products during changing of cloths or disposable pads, or emptying of the cup.^31^ The frequency was similar for menstrual cups and sanitary pads. Factors involved included young age, and lack of time and privacy. Dropping of the menstrual cup decreased with increasing experience (approximately 23% in the first 3 months and 10% at or after 12 months). This dropping was associated with *Escherichia coli* isolated in cultures from swabs of menstrual cups, which was higher in new users than in experienced users (table 2).^29^ The proportion of girls who washed their hands before changing of the menstrual cup, by verbal self-report, was 95% (204 of 215) in a Kenyan report,^28^ 70% (16 of 23) in a Ugandan report,^56^ and 94% (15 of 16) in a study in a refugee camp.^38^ When toilets have a lack of water, some participants reported carrying a bottle of water for when they emptied their menstrual cup.^28^ Others said they had to empty the menstrual cup only twice a day, so they could avoid emptying in public places.^38^ In two studies, women reported that use of menstrual cups saved water because of less leaking and washing of cloths.^33^, ^38^ Privacy was mainly mentioned as a problem when boiling (ie, cleaning) or storing menstrual cups.^38^, ^56^

We identified considerable heterogeneity in outcomes of acceptability of menstrual cups in the pooled meta-analyses (table 3). Subgroup analyses by study quality (low *vs* moderate-to-good) for outcomes examined did not show significant differences for the outcomes examined, but the sample size for moderate-to-good quality studies was small (appendix p 23). Smaller studies sometimes show different, often larger, treatment effects than large studies (ie, small-study effect); except for the outcome of “could not insert cup” (p=0·041), we did not find evidence for the presence of small-study effects (appendix p 23).

In the assessment of visibility in three studies in high-income countries, only 11–33% of the women interviewed (n=375) were aware of menstrual cups (appendix p 25).^37^, ^47^, ^55^ On 69 websites with educational materials on puberty and menarche from 27 countries, disposable pads were mentioned as an option by 53 (77%), tampons by 45 (65%), menstrual cups by 21 (30%), and reusable pads by 15 (22%; appendix pp 26–28). In the assessment of costs and availability, we identified 199 brands of menstrual cups, and availability in 99 countries with prices ranging US$0·72–46·72 (median 23·3 [IQR 16·54–29·20], from 145 brands with prices available; appendix pp 29–39).

When considering financial and environment costs, using accumulated estimates over 10 years, purchase costs and waste from consistent use of a menstrual cup (vaginal cup) would be a small fraction of the purchase costs and waste of pads or tampons—eg, if compared with using 12 pads per period, use of a menstrual cup would comprise 5% of the purchase costs and 0·4% of the plastic waste, and compared with 12 tampons per period, use of a menstrual cup would comprise 7% of the purchase costs and 6% of the plastic waste (appendix pp 40–42).

## Discussion

Women, girls, and transgender people require hygienic menstrual products monthly to live healthy and productive lives. In this systematic review and meta-analysis, we assessed the menstrual cup, combining information from medical and grey literature to inform choice and strengthen the evidence base for programmes supporting menstrual health, such as for schoolgirls in low-income and middle-income countries or among refugees. Leakage was similar or less when using the menstrual cup than when using disposable pads and tampons. The adoption of a menstrual cup required a familiarisation phase and peer support seemed to be important for uptake in low-income and middle-income countries. Challenges in resource constrained settings (eg, lack of sanitation, hygiene, and privacy) did not stop women from using the cup. Around 70% of participants in 13 studies declared wanting to continue use. We identified several incidental case reports of vaginal damage, toxic shock syndrome, or urinary tract complaints after menstrual cup use, and difficulty retrieving the menstrual cup was also reported. Use of menstrual cups has been described as a factor for IUD dislodgement. Menstrual cups were infrequently mentioned in online educational materials on puberty and menstruation for adolescent girls; the lack of information appears to be global. Brands of menstrual cups were identified in 99 countries with a wide range in prices with a median of US$23·30.

In studies that examined the vagina and cervix during follow-up, no mechanical harm was evident from use of a menstrual cup.^13^, ^52^, ^70^ Infection risk did not appear to increase with use of a menstrual cup, and compared with pads and tampons, some studies indicated a decreased infection risk.^5^, ^13^, ^70^ A study in Kenya that detected lower bacterial vaginosis in users of a menstrual cup than in those who used sanitary pads postulated that the inert material of the menstrual cup might assist in maintaining a healthy vaginal pH and microbiome.^5^ Reported pain might relate to variations in the pelvic anatomy or wrong positioning of the menstrual cup leading to internal pressure. These factors could account for case reports of hydronephrosis or urinary incontinence. Allergies to the materials used in menstrual cups are not common, but women should be aware of the possibility and keep this in mind when starting use. However, for women who start using a menstrual cup, discrimination between discomfort as part of the normal learning curve and pathology might be difficult. Laboratory studies have shown contradicting results on the possibility of development of TSST-1 in the presence of menstrual cups,^71^, ^72^, ^73^ but clinical data in humans using cups have so far not shown reason for concern.^29^ The reported risk of toxic shock syndrome with use of a menstrual cup seems low, with five cases identified via our literature search. Although aggregated data on the number of menstrual cups sold or used is unavailable, we anticipate the number of girls and women using the 199 different brands globally is likely to be in the thousands. In the USA, the incidence of all types of toxic shock syndrome was around 0·8–3·4 per 100 000 population, whereas menstrual toxic shock syndrome was reported in 6–12 per 100 000 users of high-absorbency tampons in 1980.^77^ Similarly, among women using female barrier contraceptives, which also use medical-grade silicone or latex products, toxic shock syndrome is low (approximately 2·25 cases per 100 000 users per year).^78^

The combination of an IUD and use of a menstrual cup might need further study. Women with IUDs might need to consider an alternative option for either family planning or menstrual flow. Given the few reports on menstrual cups thus far, we cannot yet exclude other issues with the use of menstrual cups. Few studies directly compared menstrual cups and other menstrual products or materials; however, data do not suggest the menstrual cup is less effective than other sanitary products. Menstrual cups can collect more blood than tampons or sanitary pads and have been adopted by women with menorrhagia.^51^ The studies we reviewed report that under challenging conditions (eg, with little water or privacy), menstrual cups can be used. Alternatives to menstrual cups and disposable sanitary pads include reusable pads, so far assessed in few studies.^75^, ^79^, ^80^, ^81^ In Uganda, privacy to dry these pads was a challenge, suggesting additional packs would be needed to ensure effective laundering.^82^

Our study had several limitations. We used leakage as a primary outcome; however, the outcomes identified in the reports and studies reviewed varied by different timepoints and designs, prohibiting combination of results when directly comparing menstrual cups with another item. The quality of studies was a limitation, with only three assessed to be of good quality, which will potentially have contributed to bias in the meta-analysis. Some data were from older studies when reporting requirements were less stringent or with menstrual cups that are no longer available, from reports not published in peer-reviewed journals, and from studies using the menstrual cup to assess other topics (eg, understanding how uncertainty barriers can be overcome in economics,^22^ use of reusable menstrual products because of environmental concerns,^37^ the association between self-objectification and attitudes toward an alternative menstrual product^47^). Recruitment in observational studies was not representative or clear. Studies mostly depended on self-reporting, which might have overestimated use of the menstrual cup. One study comparing self-reporting against a conservative but objective measure of the colour change of the menstrual cup found uptake of use of the menstrual cup was slower than self-reported—eg, by 4 months, 75% of recipients stated they had started using the cup, whereas only by 10 months did 75% of menstrual cups show appropriate colour change.^32^ The number of countries where menstrual cups were available might be underestimated because producers of menstrual cups in low-income and middle-income countries might not always have websites. Our search was in English, and thus lacked information from many countries, such as Russia or China. The heterogeneity for the pooled prevalence was high in the meta-analyses (*I*^2^≥74%), indicating inconsistency in outcomes across studies. Given the high variability in study design, study period, study population, and products examined, this heterogeneity might not be unexpected. What proportion of adverse events are under-reported is unknown; we did not identify many adverse events (one case of toxic shock syndrome) when exploring the internet (appendix pp 43–44). The MAUDE database only allows searches for the past 10 years. Our cost and waste estimates are illustrative and do not account for the combined use of menstrual products during a period or inflation and production costs.

This systematic review suggests that menstrual cups can be an acceptable and safe option for menstrual hygiene in high-income, low-income, and middle-income countries but are not well known. Our findings can inform policy makers and programmes that menstrual cups are an alternative to disposable sanitary products, even where water and sanitation facilities are poor. However, provision of information, training, and follow-up on correct use might be needed. Further studies are needed on cost-effectiveness and environmental impact comparing different menstrual products, and to examine facilitators for use of menstrual cups, with monitoring systems in place to document any adverse outcomes.
