# Supplementary material for: Menstrual cup use, leakage, acceptability, safety, and availability: a systematic review and meta-analysis
Source: Lancet Public Health. 2019 Jul 16;4(8):e376–93. doi: 10.1016/S2468-2667(19)30111-2 (PMC6669309; doi:10.1016/S2468-2667(19)30111-2)
Supplement: Supplementary appendix [file mmc1.pdf]

THE LANCET
Public Health

Supplementary appendix

This appendix formed part of the original submission and has been peer reviewed. We post it as supplied by the authors.

Supplement to: van Eijk AM, Zulaika G, Lenchner M, et al. Menstrual cup use, leakage, acceptability, safety, and availability: a systematic review and meta-analysis. *Lancet Public Health* 2019; published online July 16. http://dx.doi.org/10.1016/S2468-2667(19)30111-2.

Appendix:

Exploring menstrual products: a systematic review and meta-analysis of menstrual cups for public health internationally

Contents

Table S1. Some websites with information about menstrual cups 2

1. Supplemental methods 3
Additional information 3
Additional meta-analysis methods for quantitative studies 3
Methods qualitative analysis 4
Methods visibility and accessibility 4
Methods for comparison of costs and waste 4
Changes with protocol 4
Table S2. Adaptation of quality assessment form for observational studies 5

2. Additional Results 6
Table S3. Search results databases from inception to 14 May 2019 6
Table S4. Quality assessment of the studies involved (studies with quantitative information) 7
Table S5. Manufacturers, city, country, and websites for menstrual cups used in the studies in Table 1 8
Table S6. Other functions of the menstrual cup 9

Section 2.1. Qualitative studies 10
Table S7: CASP – Appraisal of qualitative studies 10
Table S8: Characteristics of Qualitative Papers 10
Table S9. Themes in qualitative studies 12

Section 2.2 Acceptability 14
Figure S1. Proportion of participants with menstrual cup leakage in twelve studies, by economic status of the country 14
Table S10. Use of menstrual cup, product related discontinuation and other loss to follow up 15
Table S11. Acceptability of the menstrual cup among women who tried the MC 16
Table S12. Information on mobility and odour when using a menstrual cup 17
Figure S2. Proportion of women who reported they could not insert the menstrual cup, studies between 1960 and 2017 17
Figure S3. Proportion of women who reported they used the menstrual cup at least once, studies between 1960 and 2017 18
Figure S4. Menstrual cup related discontinuation, studies between 1960 and 2017 19
Figure S5. Other reasons for discontinuing the menstrual cup, studies between 1960 and 2017 19
Figure S6. Reported difficulty with insertion of the menstrual cup by time point of evaluation, 1960-2017 20
Figure S7. Discomfort when wearing the menstrual cup, studies between 1960 and 2017 by time point of evaluation 21
Figure S8. Difficulty removing the menstrual cup, studies between 1960 and 2017 22
Figure S9. Time trends for menstrual cup use in four studies with information from developing countries 22
Figure S10. Sensitivity analyses, funnel plots and small-study effect 23
Table S13. Other outcomes examined in association with menstrual cup use 24

Section 2.3 Visibility, availability, costs and waste 25
Table S14. Evaluation of potential use of menstrual cup 25
Table S15. Menstrual items reported in education material for girls 26
Figure S11. Menstrual items mentioned in education material on menarche, from 69 websites, 27 countries 28
Costs and availability 29
Figure S12. Countries with brands of menstrual cups (source) and price available 29
Figure S13. Menstrual cup availability in 2018 30
Figure S14. Retail price of menstrual cup, by brand 31
Figure S15. Retail price of menstrual cup, by Gross National Income per capita of the country 33
Table S16. Listing of websites used to assess costs and availability of menstrual cups 35
Table S17. Price estimates for commonly used menstrual pads and tampons to obtain an average price per unit 40
Table S18. Calculations of costs and waste for sanitary pads and tampons over 10 years 41
Figure S16. Estimation of costs and waste of different products used for menstruation over 10 years 42

3. Web resources 43
Table S19. Informal information on menstrual cups and serious adverse events on the internet 44

References 45

Table S1. Some websites with information about menstrual cups

Website	Description
https://putacupinit.com	Website dedicated to the menstrual cup<br>This site has a Menstrual Cup Comparison Chart and a quiz to assist in finding the right menstrual cup
http://menstrualcup.co	Web site dedicated to the menstrual cup.
https://menstrualcups.wordpress.com	Web site dedicated to the menstrual cup. This site provides a listing of cups available and reviews
https://en.wikipedia.org/wiki/Menstrual_cup	This site has some history and a global perspective
https://www.wikihow.com/Use-a-Menstrual-Cup	This site explains with images on how to insert a cup
https://www.mycup.co.nz/menstrual-cups-environment/	This side provides information on the impact of menstruation on the environment
https://health.clevelandclinic.org/tired-of-tampons-here-are-pros-and-cons-of-menstrual-cups/	Summary of pros and cons if you don't have much time
https://www.1millionwomen.com.au/blog/cup-or-not-cup-common-questions-about-menstrual-cups-answered/	Summary of pros and cons if you don't have much time
http://www.mum.org/MenCups.htm	Information on the history of the menstrual cup
https://trends.google.com/trends/explore?date=all&q=Menstrual%20cup	Information on trends for "menstrual cup" on Google

Disclaimer: This list is an example of what is available. We do not endorse any site and do not pretend that this list is complete.

1. Supplemental methods

Additional information

Search strategy: We introduced the keywords in the databases as pairs (e.g. menstrual and cup, menses and cup) and combined searches at a second stage. In PubMed, menstrual, menses, menstruation and vaginal were used as Mesh terms and "all fields" terms. Cup is not a Mesh term. To check for completeness, we screened several other databases (See Table S2) for eligible items which were not yet included in the list resulting from the searched databases. We also checked reference lists, web material, and consulted colleagues. Records from the different searches were merged, and duplicates removed; a first screening was conducted to assess potential eligibility whereby irrelevant records were removed, based on the material available (abstract or whole text). Full text articles were obtained as much as possible for the remaining articles, and these were further screened for eligibility. With regards to identifying studies, menstrual hygiene did not need to be the main topic; e.g. studies not completely about menstrual hygiene but presenting useful information could still be included. Language: We did not exclude articles in other languages than English a-priori but decided that in case of retrieval of articles in other languages, an attempt might be done to verify content for inclusion if a person could be identified who mastered that language; however, the search was conducted in English. Searches in Google were repeated for adverse events identified in the literature (e.g. menstrual cup and retention, infection, urination, endometriosis, and toxic-shock syndrome). Reference lists of relevant studies, websites of pertinent professional bodies (e.g. FDA), non-governmental organizations and 'grey literature' (e.g., reports or conference abstracts) were searched, as well as records recommended by contacting experts, to encompass a broad range of available literature. Using pre-tested forms, we extracted data on baseline characteristics and outcomes. Study characteristics included study design, number of participants, age, location, country, time-period of study, type of MC involved, and type of menstrual item used before study, in addition to inclusion and exclusion criteria, and follow up time where applicable. If the year of study was not reported in an eligible study, we assumed it to be two years before the publication date. For studies with outcomes after e.g. cycle 1, cycle 2 and cycle 3, the result for the last cycle was taken. For studies where the follow up was longer, more time points after cycle 2 were included. For some studies, additional information from the authors could be obtained.[1-3] The Picots-framework is presented below.

PICOTS Framework

Components	
Population	Menstruating women<br>-Subgroup analysis:<br>o High income versus low and middle-income countries<br>o Age: adult vs. adolescent women<br>o Type of cup: diaphragm-type of cup (e.g. Instead, which covers the cervix) vs. bell-shape cup (with space between cervix and cup, e.g. Mooncup)
Intervention	Menstrual cup
Control	Other methods for menstrual hygiene management
Outcomes	Effectiveness:<br>1. Direct effects: Improved menstrual hygiene<br>a. Prevention of leaking<br>2. Indirect effects: effect of cup use compared to other methods on outcomes such as wellbeing and equity<br>Safety:<br>1. Serious adverse events<br>2. Effect on vagina and vaginal flora<br>3. Effect on reproductive, digestive or urinary tract<br>4. Safety when sanitary conditions are not optimal<br>5. Other<br>6. Factors affecting the use of the cup<br>Acceptability and user-friendliness:<br>1. Ease to insert, comfort of wearing, odour, mobility, ease of removal, ease of maintenance<br>a. Willingness to use among potential users or willingness to continue use among new users<br>2. Costs and availability (cup master list)<br>3. Knowledge about option of cup among the arsenal of options for menstrual hygiene management (separate search)
Timing	No restriction
Setting	Any trial, quasi-experimental study, survey or other type of study reporting on experiences with cup use for menstrual hygiene management. Search in English

Additional meta-analysis methods for quantitative studies

When using meta-analysis, the extent of heterogeneity was measured using the I^2 statistic,[4] which is a measure of the proportion of total variability explained by heterogeneity rather than chance expressed as a percentage, with 0–40% representing no or little heterogeneity, 30–60% moderate heterogeneity, 50–90% substantial heterogeneity, and 75–100% considerable heterogeneity.[5] We examined the following sources of heterogeneity if sufficient data were available using subgroup analysis: setting of the study (high-income [HIC] versus LMIC), study population (adult women versus girls aged 12-17 years), year of study (study conducted before or after 2000), type of menstrual cup used (cervical versus vaginal cup) and duration of menstrual cup use. We assessed publication and small-study bias by visual inspection of funnel plots for the outcome of reported MC use in the future.[6,7]

Methods qualitative analysis
We followed the Critical Appraisal Skills Programme tool to examine the quality of the studies.[8] While the tool does not provide a definitive score to judge studies on their quality and / or include or exclude them, it enables the reader to understand the rigour of each study and the transparency of reporting, thus facilitating judgement of quality and interpretation of results. Two reviewers (LM and GZ) assessed each study independently, and examined and summarised the findings from these studies in combination to ensure a more robust and comprehensive explanation than would occur if each individual study was considered in isolation.[9] The data were analysed using thematic synthesis as described by Thomas and Harden (2008).[10] Separately, the 2 qualitative reviewers extracted the themes and subthemes, along with illustrative quotes where applicable, from a first paper, and repeated this process for additional papers until a complete table of themes and subthemes with quotes was devised. A comparison of both tables was made, and a single definitive table produced. From this, the reviewers identified six key themes and eight subthemes, which were written up in narrative form with illustrative quotations. These qualitative results were integrated with quantitative results in the results section, but the full results are also available in Supplement section 3.1.

Methods visibility and accessibility
To assess visibility of the menstrual cup, we used websites of governments and non-governmental organizations to obtain education materials to prepare girls for menarche from countries where the review-authors were familiar with the language (English, German, French, Spanish, and Portuguese; conducted by GZ, ML & AMvE). Search terms for this part included a country name, "menstruation", and "information", separate and in combination with "adolescent girls", and terms were translated where applicable. Websites from commercial providers of menstrual items were excluded, where identified. We compiled if sanitary pads, tampons and menstrual cups were reported in the material.

To assess the availability and price of menstrual cups, we used the "Menstrual Cup Master List", a web source of vaginal menstrual cups available globally,[11] and other sources (e.g. websites from menstrual cups and experts) to find countries where brands were based. Each individual brand was searched to identify its availability for purchase, through the web or otherwise. We determined if a cup was available in a country. If it was available for sale in a retail store or if you could purchase it directly through their website or an intermediary ecommerce website locally. Where possible we identified the countries in which each brand was purchasable, and its respective price. When brands had no country of origin listed, company FAQs and consumer reviews were scanned to identify brand location. Prices reported in local currencies were converted to United States Dollars with the applicable exchange rate on 31 August 2018. If there were a range of menstrual cup products available for purchase, we selected the "original" model. If items were available for bulk purchase at a subsidized cost, we selected the purchase price when buying an individual unit. If individual units could not be purchased, we divided the bulk price by the number of units to calculate the individual unit cost. When pricing differed across adolescent and adult users, we selected the "adult" size for comparison purposes. We also checked with experts to find out if there were cups which were missing on the list. Between July 2017 and August 2018, 145 brands available for purchase (141 with an identified country of origin) were compiled. When separating cups by country wealth quartile, we classified countries according to the 2017 Atlas rankings developed by the World Bank. This method sorts countries by Gross National Income per capita.

Methods for comparison of costs and waste
For estimates on use of pads and tampons, we used estimates from included studies.[12-14] However, because use of sanitary products may depend on type of flow, we decided to examine a range, varying from 9 to 25 items per month (Table S16). For estimations on costs of pads and tampons, we explored prices for Tampax (cardboard applicator) and OB tampons, and Always and Kotex pads in the USA, the UK, India, Spain, China and Canada on Amazon Prime (data obtained on 5 October 2018, GZ & AMvE); prices were converted in US dollars using the conversion rate on August 31, 2018 and calculated price per pad or tampon (Table S12). The average cost for one tampon and one pad was 0.21 dollar (range 0.12-0.38) and 0.31 US dollar (range 0.15-0.55), respectively. For estimates on weight and information on biodegradable content we used the report by Mazgaj et al. (2006) for a Libresse pad and OB tampon.[15] We used 6.8 grams as the weight of a menstrual pad and a plastic content of 35% of the weight; corresponding numbers for tampons were 3.6 grams and a plastic content of 4% of the weight. As an estimate of monthly blood loss we used 35 ml (normal range is 5-80 ml).[16] For the menstrual cup estimates we assumed a vaginal cup with a life time of 10 years, weighing about 15 grams, and the median price of a menstrual cup in US dollars as previously obtained. We did not include the amount of blood lost in the calculation of waste for the menstrual cup, because this blood is not absorbed as in a tampon or pad.

Changes with protocol
We were unable to access the database Psychinfo. We screened the first 50 pages (500 entries) of Google Scholar. In addition, we screened the databases Science.gov and WorldWideScience to check if our information was complete. We excluded studies where the MC was purely used to obtain gynaecological samples, even if data on the experience of participants was presented, because generally their experience only covered a select period of one cycle. For quality assessments of included cohort studies we used an adaptation of the Newcastle-Ottawa tool. Limitations of this tool were the absence of a clear non-exposure group and verification of exposure and outcome. In studies where experiences among menstrual cup-users were compared with their previous experiences with other menstrual products, we assumed a before-after design, with a non-exposed group having the same characteristics as the exposed group. A study was considered moderate-to-good, if at least 4 criteria had been satisfied in the adapted Newcastle-Ottawa score (Table S1) or 5 in the Cochrane tool for trials. Because of the wide variability in types of reported outcomes, e.g. for leaking, we could not combine results from different studies using a risk ratio. We compared reported outcomes; however, given that most studies were "before-after design", using the same women reporting for experiences with MC and routine products, the use of a chi-square test would have been inappropriate but insufficient information was available for a

McNemar test. We intended to use the quality assessment as part of meta-regression and sensitivity analysis but decided not to conduct meta-regression because of the limited number of high-quality studies for meaningful comparisons, and the limited added value when conducting meta-regression.

Table S2. Adaptation of quality assessment form for observational studies

	**Focus area**	**Category options†**
1	Representativeness of the exposed group<br><br>a) Is a description how women were recruited present?<br>b) If there is, are women representative of women in the community?	a) truly representative of women in the community (e.g. random selection in community) *<br>b) somewhat representative of the average woman in the community *<br>c) selected group of women (e.g. women who were enrolled from a clinic population)<br>d) no description of the derivation of the group<br><br>*Adaptation: 1 point if description present and women representative of women in the community (if not clear how recruited, or recruited from a clinic: 0 points).*
2	Selection of the non-exposed group	a) drawn from the same community/pool as the exposed group *<br>b) drawn from a different source<br>c) no description of the derivation of the non-exposed group<br><br>*Adaptation: If information is used from women before they were enrolled (e.g. a before-after study), or if there is control group that is coming from the same source as the exposed group: 1 point. Otherwise 0.*
3	Ascertainment of exposure	a) Inspection cup or colour change cup, or cup in situ<br>b) combination of cup inspection and interview<br>c) unsecure record<br>d) written self-report<br>e) no description<br><br>*Adaptation: If cup colour change used 1 point, otherwise no objective measure: 0 points.*
4	Comparability of exposed and unexposed group	a) differences examined and no differences reported in characteristics which are presented *<br>b) differences in characteristics present but no effect on outcome, or multivariate analysis for outcome available or randomized study *<br>c) differences in characteristic present, not shown if effect on outcome<br>d) no description/not examined<br><br>*Adaptation: If it is comparable to a before-after study or a comparison among characteristics of women who used and did not use the cup and no differences, 1 point. If it is not present, 0 points.*
5	Outcome assessment (leakage, frequency of change, vaginal inspection, bacterial cultures): not possible	a) independent blind assessment (observations or laboratory tests) *<br>b) record linkage (for observations or laboratory tests) *<br>c) not clear<br>d) no blind assessment (verbal or written reports)<br>e) no description<br><br>*Adaptation: if laboratory tests or inspection of vagina 1 point, if verbal report 0 points.*
6	Attrition	a) complete - all subjects accounted for *<br>b) outcome not available for all subjects but unlikely to introduce bias - small number lost - <20%, or description provided of those lost *<br>c) outcome for less than 80% of people with exposure data and no description of those lost<br>d) no statement

* A study could be awarded a maximum of one point for each item, so in total 6 points.

2. Additional Results

Table S3. Search results databases from inception to 14 May 2019

	**Source and website**	**Result (number)**
1	PubMed<br>https://www.ncbi.nlm.nih.gov/pubmed	179
2	Web of Science (or Knowledge)<br>https://webofknowledge.com	217
3	Cochrane library<br>http://www.cochranelibrary.com	79
4	Popline<br>https://www.popline.org	41
5	Medline, Global Health and Cinahl through Ebscohost<br>https://www.ebsco.com/products/research-databases/global-health<br>https://www.ebscohost.com/nursing/products/cinahl-databases/cinahl-complete	290
6	Emerald<br>http://www.emeraldinsight.com	30
7*	Science.gov<br>https://www.science.gov	200 screened<br>(3 new)
8	WorldWideScience<br>https://worldwidescience.org	200 screened<br>(1 new)
9	Google Scholar<br>https://scholar.google.com/	500 screened<br>(30 new)
10	Co-authors/references	9 new
11	FDA Database (Maude)	15 case-reports†

Keywords: (Menstrual AND Cup) OR (Menses AND Cup) OR (Menstruation AND Cup) OR (Vaginal AND Cup)
* Screened databases (7 to 9) were checked for eligible items which were not yet included in the list resulting from databases 1-6
† The FDA Database was last searched on 28 May 2019. Four case-reports on softcups excluded before 2011 because of potential overlap with reporting by North and Oldham (2011).[14]

Table S4. Quality assessment of the studies involved (studies with quantitative information)

Table S4a. Trials

Study	Random-sequence generation	Allocation concealment	Blinding participants /staff	Blinding outcome assessment	Incomplete outcome data	Selective reporting	Other bias	Total
Beksinska 2015[13,17,18]	Computer-generated	Yes "Allocations were concealed in sequentially numbered sealed envelopes"	No	No (cross-over)	5/110 (4.5%), balanced over arms	Not detected	None detected	High quality 5
Hoffman 2014[19]	Roll of dice	Yes, sealed envelop	No	Not reported	15.8%, balanced over arms (no data)	Not detected	Cross-over of arms diluted MC effect	Low-to-moderate 4
Howard 2011[12]	Computer-generated	Not specified	No	Not reported	13/110 (11.8%), balanced over arms	Not detected	Sample size not achieved	Low-to-moderate 3
Oster 2011[20-23]	Lottery (drawing numbers from bag)	Yes	No	Not reported	1/199 (0.5%), balanced over arms	Follow up time per arm not reported. Ambiguous outcome.	Girls who were not yet menstruating were not excluded, unequal over arms	Low-to-moderate 3
Phillips-Howard 2016[1]	Drawing balls from bag and concealed envelopes	Yes, concealed envelopes	No	Yes (allocation concealed from laboratory staff and trial statistician)	122/776 (15.7%), balanced over arms	Not detected	None detected	High quality 6

Table S4b. Observational cohort studies

Study	Women representative of community	Non-exposed from same group	Ascertainment of exposure	Comparability of groups	Outcome assessment (observed/laboratory test vs. verbal/written)	Loss to follow up	Total
APHRC 2010[24-26]	Unclear	Yes (assessed as before/after study)	Verbal report	Same group	Verbal/written report	6/96 (6.3%)	Low-to-moderate 3
Care International Uganda 2018[27]	Unclear	Yes (assessed as before/after study)	Verbal report	Same group	Verbal/written report	43/80 (53.8%)	Low-to-moderate 2
Cattanach 1990, 1991[28,29]†	No (private clinic, volunteers)	Yes (assessed as before/after study)	Verbal report	Same group	Verbal/written report	259 women contacted and 80 respondents (69.1% attrition)	Low-to-moderate 2
Cheng 1995[30]	Unclear	Yes (assessed as before/after study)	Verbal report	Same group	Verbal/written report	Not reported	Low-to-moderate 2
Chintan 2017[31]	Not representative (women in waiting area clinic)	Yes (assessed as before/after study)	Verbal report	Same group	Verbal/written report	Not reported	Low-to-moderate 2
Femme International[2] ‡‡	Not representative (School girls and adult women)	Yes (assessed as before/after study	Verbal report	Same group	Verbal/written report	Not reported or high (37-88%) (different study populations)	Low-to-moderate 2
Ganyaglo 2019[32] Ryan 2018[33]	Women with vesicovaginal fistula	Yes (assessed as before/after study)	Unclear	Same group	Measurement of urine leakage	0%	High quality 5
Gleeson 1993[34]	Not representative (clinic).	Yes (assessed as before/after study)	Verbal report	Same group	Verbal/written report	0%	Low-to-moderate 3
Kakani 2017[35]	Not representative (clinic)	Yes (assessed as before/after study)	Verbal report	Same group	Verbal/written report	8/158 (5.1%)	Low-to-moderate 3
Madziyire 2018[3,36]‡‡	Not representative (family planning clinic)	Yes (assessed as before/after study)	Verbal report	Same group	Verbal/written report	2/54 (3.7%)	Low-to-moderate 3
North 2011[14]	Not representative (clinics)	Yes (assessed as before/after study)	Unclear	Same group	Verbal/written report, observations and laboratory tests	98/406 (24.1%)	Low-to-moderate 3
Parker 1966[37]	Not representative (clinics).	Yes (assessed as before/after study)	Verbal report	Same group	Verbal/written report	7/46 (15.2%) Loss not clear for 19 women with normal menses.	Low-to-moderate 3

Pena 1962[38]	Not clear how recruited	Yes (assessed as before/after study)	Verbal report	Same group	Verbal/written report, vaginal inspection	Not reported	Low-to-moderate 2
Shihata 2014[39]	Not representative (clinics)	Yes (assessed as before/after study)	Verbal report	Same group	Verbal/written report	41/146 (28.1%)	Low-to-moderate 2
Stewart 2010[40]	Not representative (students)	Yes (assessed as before/after study)	Verbal report	Same group	Verbal/written report	33/54 (61.1%)	Low-to-moderate 2
Tellier 2012[41]	Not representative (including staff)	Yes (assessed as before/after study)	Verbal report	Same group	Verbal/written report, observations and laboratory tests	16/31 (51.6%)	Low-to-moderate 3
Wiebe 2012[42]	Representative for IUD users	Yes (assessed as before/after study)	Verbal report	Same group	Retrospective chart survey; linking	Can't be assessed.	NA

Table S4c. Observational surveys (no score assigned)

Study	Study population	Random sample	Sample size, refusal rate
Averbach 2009[43]	Convenience sample from public clinics, markets and shopping centres	No	43, not reported
Borowski 2011[44]	Colleagues, friends, and family of co-workers, survey link on Facebook	No	160, not reported
Grose 2014[45]	Female undergraduates	No	168, 5 excluded (using MC) and 12 excluded for incomplete forms
Stewart 2010[46]	Patients attending a menstrual disorder clinic	No	69, not reported

Table S5. Manufacturers, city, country, and websites for menstrual cups used in the studies in Table 1

Cup	Manufacturer	City/country	Website
**Be'Cup**	Laboratoires Juva Santé	Paris, France	https://www.becup.fr/
**Butterfly**	Butterfly Cup Company	Harare, Zimbabwe	http://www.thebutterflycup.co.zw/
**Diva cup**	Diva International, Inc.	Ontario, Canada	https://divacup.com/contact-us/
**Duet**	ReProtect, Inc.	Baltimore, USA	http://www.reprotect.com/products.shtml
**FemmyCycle**	FemCap Inc	Del Mar, USA	https://femmycycle.com/
**Flow Care**	Not clear	India	No website identified. Not clear if still in production
**Gynaeseal**	Chattan Australia Ptv. Ltd	Australia	Out of production
**Keeper**	The Keeper, Inc	Cincinnati, USA	https://keeper.com/
**LadyCup**	Jaguara, s.r.o.	Offices in Prague, Czech Republic, Stuttgart, Germany, and Valley Cottage, USA	https://www.ladycup.eu/
**MeLuna**	Me Luna GmbH	Haundorf, Germany	https://www.me-luna.eu/en_GB https://meluna-usa.com
**Menses cup**	Shanghai Newboasia Medical Rubber Factory	Shanghai	Not clear if still in production
**Mooncup**	Mooncup Ltd	Brighton, UK	https://www.mooncup.co.uk
**Mpower / Mcup**	Mpower	Cape Town, South Africa	http://mpowercup.co.za/
**Ruby cup**	Ruby Cup, Ruby Life	Barcelona, Spain and London, UK	https://rubycup.com
**Softcup**	Instead, Inc., San Diego, CA Now softdisc, Flex company	Venice, CA, USA	http://softdisc.com
**Tasette**	Tassette, Inc.	Stanford, CT, USA	Out of production
**Tassaway**	Tassette, Inc.	Stanford, CT, USA	Out of production

MCs in alphabetical order

Table S6. Other functions of the menstrual cup

Function	Cup type	Numbers (%) or description	Notes	Source
Reduction or control of leakage from enterovaginal or vesicovaginal or vesicouterine fistula	NR (vaginal)	Case report: Symptoms of urine leakage improved during menses when she used a menstrual cup	"When evaluating women with urinary incontinence and a history of caesarean deliveries, use of menstrual cup may aid in the diagnosis of vesicouterine fistula."	Goldberg 2016[47]
	NR (vaginal)	2 case reports	"In two cases of vesicovaginal fistula, the menstrual cup was used with great success for control of urinary leakage"	Parker 1966[37]
	NR (vaginal)	Three case reports of menstrual cup use to assist in palliative therapy	"This simple and inexpensive device should be considered in those cases in which the drainage can be diverted as a viable option, especially in those who are symptomatic and awaiting surgical repair or in those for whom surgery cannot be performed."	Russel 2016[48]
	NR (vaginal)	Study to assess the feasibility and acceptability of the vaginal menstrual cup for short-term management of vesicovaginal fistula (VVF) among 11 women seeking treatment at a health facility in Ghana.	"The repeated measures design utilizes a 2-hr pad test to compare urinary leakage with and without the insertable cup…" "With the cup, women experienced an average 61.0% (±37.4) (95% CI: 35.9–86.2) leakage reduction, a difference 10/11 users (91.0%) perceived in reduced leakage." "Acceptability and appropriateness among women was high as most could easily insert (72.73%), remove (72.73%), and comfortably wear (100%) the cup"	Ganyaglo 2019[32] Ryan 2018[33]
Contraceptive	Gynaeseal (reusable cervical cup)	33/73 (45.3%) women used the cup as contraceptive: 1 unplanned pregnancy (follow up 18 months after start of study)	In this study it was not used in combination with spermatocides	Cattanach 1991[29]
Facilitator of intercourse during menstruation	Soft cup (disposable cervical cup)	The soft cup was worn during intercourse by 67 subjects in cycle 1, 61 in cycle 2, and 58 in cycle 3. Nine women reported discomfort, 13 partners reported discomfort.		North and Oldham 2011[14]

Other use of MCs

The cervical cup has been referenced as a possible product to allow intercourse while menstruating with potential as a contraceptive.[29] Vaginal cups have been used for the diagnosis, reduction or control of leakage from enterovaginal, vesicovaginal, or vesicouterine fistulae.[32,37,47,48]

Section 2.1. Qualitative studies

Study characteristics qualitative studies

Of the six studies identified with qualitative information, three were methodologically weak, one was satisfactory and two were judged as high quality (Table S6).[24,25,41,49-51] No studies were excluded due to the limited number; however, in recognition of the lack of robust evidence we selected findings that were evidenced in at least 3 of the 6 studies. The six studies (Table S7), all in developing countries, included a total of at least 225 participants (the study by Care International 2018,[43] did not report total number of participants taking part in the FGDs), although not all had received a cup;[50,51] 75 participants took part in an interview (described as in-depth interview or semi-structured or personal interview, 47.2% school girls) and a minimum of 193 were in 21 focus group discussions (FGDs, 72.5% school girls). Data were collected at 3-6 months after participants received a cup (not available for one study). Four studies described the participant selection strategy.[41,49-51] Two studies reported data triangulation and triangulation of analysis.[49,51]

Table S7: CASP – Appraisal of qualitative studies

	Clear statement of aims	Qualitative Method appropriate	Will design address aims	Recruitment appropriate	Data collected to address issue	Relationship considered	Ethical issues considered	Data analysis rigorous	Clear statement of findings	How valuable is research	Overall quality
Hyttel 2017[49]	Yes	Yes	Yes	Yes	Satisfactory	Cannot tell	Yes	Satisfactory	Yes	Strong	Medium
Mason 2015[51]	Yes	Yes	Yes	Yes	Satisfactory	Satisfactory	Yes	Satisfactory	Yes	Strong	Strong
Sundqvist 2015[50]	Yes	Yes	Yes	Yes	Yes	Yes	Yes	Yes	Yes	Strong	Strong
Tellier 2012[41]	Yes	Yes	Satisfactory	Cannot tell	Cannot tell	Cannot tell	Cannot tell	Cannot tell	No	Weak	Weak
APHRC 2010[24,25]	Yes	Yes	Yes	Cannot tell	Cannot tell	Cannot tell	Cannot tell	Cannot tell	No	Weak	Weak
Care 2018[27]	Yes	Yes	Minimal	Cannot tell	Cannot tell	Cannot tell	Cannot tell	Cannot tell	No	Weak	Weak

Table S8: Characteristics of Qualitative Papers

Author, year of publication	Country Time	Sample	Age Range (years)	Study Design and comparison	Duration of cup use	Data Collection Method	Analysis Method	Research topic / scope
Hyttel 2017[49]	Uganda 2013	36 schoolgirls	13-17	Qualitative study within RCT. Cloths and single use pads	4 months	12 Interviews<br>4 FGDs	Thematic analysis	Drivers of cup use<br>Challenges of MC use
Mason 2015[51]	Kenya 2012-2013	101 schoolgirls<br>64 parents	14-16<br>Parent age NR	Qualitative study within RCT.<br>Cloths, single use pads, other	6 months	10 girls FGDs*<br>6 parents FGDs*	Thematic analysis	Experience of MC and pad use
Sundqvist 2015[50]	Tanzania 2014	15 schoolgirls<br>9 parents*<br>4 teachers*	14-15<br>Adult age NR	Qualitative study cup donation project.<br>Cloths, single use pads, other	NR	24 Semi structured interviews<br>1 FGD (4 teachers)	NR	MC usefulness and impact on female empowerment
Tellier 2012[41]	Uganda NR	31 women	18-32	Pilot study – mixed methods. Cloths, single use pads	3-5 months	Semi structured interviews: 7 persons at baseline, 15 at follow up	NR	Acceptability and safety of MC use
APHRC 2010[24,25]	Kenya 2008	24 schoolgirls<br>17 women	NR	Mixed methods. Cloth, single use pads, cotton wool, tissue paper	4 months	In-depth interviews: 24 girls baseline, 22 at 4 months, 17 women both	NR	Attitudes and acceptability
Care International Uganda 2018[27]	Uganda 2018	NR	15-30<br>25 ≤18	Mixed methods. Disposable and reusable pads, cloths	3 months	7 FGDs, 3 “personal interviews”, both at endline	“Thematic coding”	Experience of using the MC and menstruation

FGD=Focus Group Discussion. MC: Menstrual cup. NR=not reported. *Mason et al. (2015): 4 FGDs among cup users, and 2 FGDs among parents or teachers of girls who used cups.

Description of qualitative results

Although a range of themes were evident within the studies we identified the following 6 themes as important, with evidenced in all or most of these studies with supporting quotations and / or author narrative: Initial apprehensions; Becoming accustomed; Resolving issues, restoring dignity; Challenges; Effect on schooling; Economic benefits. The first three themes provided the most material and numerous similar quotations and appeared to be stronger themes overall.

<u>Initial apprehensions</u>: Within this theme, subthemes arose around fear of the unknown, fear of the size, and fear of adverse events. All studies noted that participants frequently opined how shocked they were on viewing the cup for the first time, little realising how big it would be: ‘*but first days when we saw it we thought – it is too big! It cannot fit!*’ Many voiced concerns that it might

cause pain (and indeed noted that it did often did so at first) or even had potential to cause reproductive harm to the wearer. However, there was little or no evidence presented in these studies that this had much lasting effect in preventing participants from using a cup. Three studies additionally described members of the community also giving similar comments to the participants such as '*I still fear because they said that …..[the cup] had been brought to us so that girls don't produce'*).

Becoming Accustomed: All studies described ways in which girls became accustomed to wearing the cup with time, practice, peer support and training being key to successful use. '*The first time, I found it difficult and … I came back to use pads. Then another month began. I tried [the cup] and felt like my waist was paralyzed. I then used it and the next day I used it and I then found using it became easy and I now use it.*

Noted in 3 of the studies, peer support appeared important in overcoming this barrier, with quotes suggesting that having peers describe cup usage in positive terms, or sharing ways of inserting / wearing, would encourage struggling participants to try and master usage for themselves. '*In the beginning I was fearing but my friend also told me just pick it, maybe you will like it later…My friend said I am even using it and I am so free, so I decided to use it. If not, I would have been waiting'*. It appeared that information provided in cup training particularly by those teachers / researchers who used the cup themselves, may also have had a positive effect in supporting and encouraging participants to resolve any issues, although there was minimal supporting quotations provided within the papers.

Resolving Issues, Restoring Dignity: Within this theme 3 key subthemes were apparent, leakage, comfort (also seen as health benefits), and feeling 'free' – these going some way to reduce the shame and embarrassment and isolation that menstruating girls and women suffered from. There were frequent positive quotations suggesting that the menstrual cup was a solution to problems they frequently faced during menstruation which included leakage, staining of clothes and on occasion dropping of the absorbent which resulted in embarrassment or shame). All studies evidenced participants describing leakage as a problem which was resolved when using a cup. They described relief from constant worry that their clothes would be stained, or the need to keep checking this did not happen, which appeared to be the norm with traditional items and even with sanitary pads. '*You feel free, unlike pads, because pads you have to check all the time….. you even sleep freely, no problems with anything'* . Comfort was also repeatedly described as a benefit that participants observed when using a cup in comparison to their experience with alternatives. Recurring comments stemmed around the inability to feel it once inserted correctly, whilst there was also occasional comparison with pain, discomfort or bruising or itching that can occur with a pad or cloth, hence also being also termed 'health benefits'. Feeling '*free*' from the physical restrictions of wearing a pad or a cloth also appeared to be another positive outcome reported around cup use as it gave participants liberty to undertake physical activities that were restricted or impossible when using other forms of menstrual protection such as bike riding, playing, running etc. '*It was as if I am not on my period…[The cup] doesn't leak, it is comfortable and I can even bike. It doesn't hurt like disposable pads, where sometimes I wouldn't feel like even asking. Now I am free to do whatever I want and walk and bike as long as I want*'. However, freedom also came from the lack of worry, such as not having to constantly check for leakage.

Challenges: A range of challenges were evidenced in relation to using a cup. Three of the studies described some difficulties with cleaning and storing of the cup, these included finding a suitable container in which to boil the cup, having to ensure that cleaning is done in private as well as keeping the cup safe from rats or being stolen in between menses. Other challenges related to having water to wash the cup, changing the cup, notably in school or public toilets in the event of a queue or with minimal time, and also at night where security was an issue. '*it would be tricky going to empty the cup in the public toilets especially when you are travelling and maybe there is a queue, someone is banging on the door for you to finish quickly, and in the process of hurrying, it can easily drop in the toilet*'.

Effect on Schooling: The influence that cup use had on school attendance was described in 3 studies with the implication that attendance was improved since participants received a cup. '*That thing is also good because let's say you are using pads and then the pads gets finished before you have finished your periods and there is no money to buy more so for you, you will stay at home but school continues normally; You stay at home because you fear the leaking, that is where I find the difficulty'* The same studies also reported on the improvement in concentration and freedom from worry which may in turn improve performance and these were linked to the subthemes described above of reduced leakage, comfort and feeling free.

Economic Advantage: Lastly, the economic advantage of having a cup emerged as a finding in 4 of the studies, with participants (and families) happy not to have to spend or 'waste' money each month on a pad or soap for washing cloths. It gave them the opportunity to purchase other essentials. In some cases, this appeared to be a key incentive in adopting up usage despite initial fears. Just one study included a participant quote illustrating that it also reduced the need for transactional sex to purchase pads.

Table S9. Themes in qualitative studies

Theme	Source Text	Studies
Initial apprehension	"In the beginning I was fearing but my friend also told me just pick it maybe you will like it later..….. I also talked to other girls myself, who feared trying it. All I know are now using it.' [41]<br><br>'When they distributed the cup I also feared much. My younger sister inserted the thing first, and then I asked her whether it is painful. She replied yes it is painful. I then said if it is painful I will not insert it, but I then took courage and inserted it.' [49]	24,25,41,49-51
The unknown	'I thought it was a strange thing, I had never seen it before. I had never heard about it [...] I felt it was very strange.' [50]<br>'but first days when we saw it we thought – it is too big! It cannot fit!' [51]	
Size	'We like [the cup] but when we had just received it and went home with it our mothers were saying that thing will destroy our uterus; it is big, it will widen our female organ and enlarge our uterus.' [51]	
Fear of adverse events	'A certain woman told me not to use that thing because it can prevent me from giving birth in future.' [51]<br><br>'I still fear because they said that [the cup] had been brought to us so that girls don't produce.' [49]	
Becoming Accustomed	'I felt pain, then I said to myself 'this thing is difficult to insert' then I said again, 'no, I heard people talking that it is good', so I went to try again. So from that day up to now I have been using it.' [51]	24,25,41,49-51
Peer experience	'In the beginning I was fearing, but my friend also told me just pick it, maybe you will like it later….My friend said I am even using it and I am so free, so I decided to use it. If not I would have been waiting. I also talked to other girls myself, who feared trying it. All I know are now using it.' [41]	
Practice	'The first time, I found it difficult and…I can back to use pads. Then another month began. I tried [the cup] and felt like my waist was paralysed. I then use it and the next day I used it and I then found using it became easy and I now use it.' [49]<br><br>'Why I felt that that that thing is good was because of how they explained; how to fold, insert, all made me feel that using it is easy and I joined and received it.' [49]	
Training & support	'If she says [the cup] is difficult to use, you tell her that she should try it this way. You tell her how you tried and that is why you got used to it.' [49]<br><br>'I thought I had to try to because during the education they said that it can feel weird in the beginning but if you repeat and repeat it is better. So I was thinking, I should repeat and repeat and then it started feeling fine.' [50]	
Resolving Issues, Restoring Dignity	'Because when you put the cup, the blood cannot come out. But with the pad, it can move aside so you get blood on the outside, you get dirty and feel ashamed.' [50]	24,25,27,41,49-51
Leakage	'It has helped me because before if I use [brand] sometimes I could find blood stain on my clothes and you know that is embarrassment, but since the Mooncup was brought, if I insert it I just feel free and do not even have it in my mind… ' [51]<br><br>'…in class you are not worried that you will spot and people will know that you are menstruating. It doesn't leak and I no longer have to keep running to the toilet to change.' [25]<br><br>'it collects all the blood inside and it doesn't leak like when you are using the pad, which you have to keep changing, otherwise it leaks.' [49]	
Comfort / health	'You will also avoid itching because of the dryness.' [25]<br>'Because it doesn't give bruises or blisters. You feel more comfortable, you feel free.' [50]<br>'[using the cup] is so much more easy. When pads have been there for long, it gets too warm, and uncomfortable. It was as if I am not in my period..[The cup] doesn't leak, it is comfortable and I can even bike. It doesn't hurt like disposable pads, where sometimes I wouldn't feel like walking. Now I am free to do whatever I want, and walk and bike as long as I want.' [41]<br><br>'It is good because you cannot get skin rashes, unlike pads, after using you will have irritations and maybe bruises, but Mooncup does not.' [51]<br><br>'You are very comfortable and you can do so many things with the cup on. You will walk freely with your head high.' [25]	
Freedom	'I feel free, unlike pads, because you have to check all the time, you also cannot drive your bike. Now I can drive my bike and I don't have to look back, I don't fear, now I can stay for a long time at gatherings. You even sleep freely, no problems with anything.' [41]<br><br>'I'm feeling good because when I put that Mooncup inside I can run, I can do anything.' [51]<br><br>I found using it was interesting and easy because once you have inserted it you can play so freely you will not feel that there is something in your body.' [49]	
Challenges	'At school there are times when there is no water and during those times I don't remove my cup at all until I reach home. Because I need water to pour in the toilet and also to rinse the cup with because I cannot leave the toilet like that.' [25]	24,25,27,41,49
Water / cleaning / security / changing	'In Korogocho you cannot go out at night so I have a plastic container that I use. I pour the menstrual blood in it, rinse the cup with water and use it again. In the morning I can go and pour that waste in the toilet and then wash the container.' [25]<br><br>'there was a problem of getting a tin for boiling.' [49]<br><br>'I received re-usable pads but because they required so much water, my grandmother, who I live with, discouraged me from using them because they were wasting the little water we had'[27]	

	'Some people…they understand that it is used like this, so that they get a day and steal it, and indeed for some people [their cup] was stolen because they explained it all.' [49]	
Effect on Schooling Attendance<br><br><br><br><br>Performance / concentration	'My periods used to come so much that it would even prevent me from coming to school. When I used that cup I found it was good because once it is full I would know and go and change. Even now I stay freely among my friends.' [49]<br><br>'It can improve their performance because they will now concentrate more on their studies, not on how she will manage her periods because sometimes the teacher is busy teaching and she is just thinking about periods.' [51]<br><br>'I won't have to keep on checking and being worried that it might leak, unlike the pad which I have to be checking all the time. I will be able to concentrate in class. This will really help.' [25]<br><br>'Before I got the cup I found it really difficult when I was in my period to go to school. I really feared shame if I was seated and the blood can smell. Really I had that fear. But since I received the cup I can put in the cup and it can just hold the blood.'[27]	24,25,49,51
Economic Benefits	"I had a lot of fear in the beginning, but I kept on using it because I could see that it was a smart and cheap solution.' [41]<br><br>'I used to spend 5,000 Ugandan shilling for pads, now I can spend it on something else, I am so happy for that.' [41]<br><br>'That cup I liked it because they said it will last for 10 years, so I felt that for the10 years, it will help us not to waste money.' [49]<br><br>'You will save costs of water and soap.' [25]<br><br>'When I [attended] the education of the cup I was thinking that the pads are expensive for me and that the cup will not be expensive for me, so I thought it's better to learn to use it and save money.' [50]<br><br>Since the project started I now feel comfortable because when I use the cup, instead of thinking where I'm going to get money to buy pads, I am using the cup and the cup is really helping me'[27]	24,25,27,41,49,50

Section 2.2 Acceptability

Figure S1. Proportion of participants with menstrual cup leakage in twelve studies, by economic status of the country

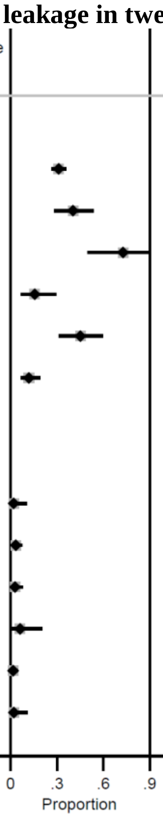

Study	Country	Population	Type of cup	Menstrual cup	Sample size	Proportion (95% CI)	Definition Leakage	Follow up
High Income Country								
North 2011	USA	Adult women	Cervical	Softcup	383	0.31 (0.26, 0.36)	Occasional leakage in 3 cycles	3 cycles
Cattanach 1991	Australia	Adult women	Cervical	Gynaeseal	62	0.40 (0.28, 0.54)	Leakage on at least one occasion	18 months
Gleeson 1993	Ireland	Adult women	Cervical	Gynaeseal	22	0.73 (0.50, 0.89)	Leakage in first cycle	1 cycle
Parker 1966	USA	Adult women	Vaginal	Tassette	45	0.16 (0.06, 0.29)	Moderate or excessive leakage	2-6 months
Cheng 1995	Canada	Adult women	Vaginal	Valve cup	51	0.45 (0.31, 0.60)	Leakage in first cycle	1-13 cycles
Shihata 2014	USA, Sweden	Adult women	Vaginal	FemmyCycle	119	0.12 (0.07, 0.19)	Leakage cycle 1 or 2	3 cycles
Low & Middle Income Country								
Madziyire 2018	Zimbabwe	Adult women	Vaginal	Butterfly cup	52	0.02 (0.00, 0.10)	Leakage in cycle 3	12 months
Kakani 2017	India	Adult women	Vaginal	NR	150	0.03 (0.01, 0.08)	Leakage in cycle 3	3 cycles
Beksinska 2016	South Africa	Adult women	Vaginal	Mpower cup	106	0.03 (0.01, 0.08)	Occasional leakage in 3 cycles	3 cycles
APHRC 2010	Kenya	Adult women	Vaginal	Mooncup	33	0.06 (0.01, 0.20)	Leakage as reason to stop cup use	3 cycles
Phillips-Howard 2016	Kenya	Girls	Vaginal	Mooncup	192	0.02 (0.00, 0.04)	Replacement larger size for leakage	Median 7.4 months
APHRC 2010	Kenya	Girls	Vaginal	Mooncup	49	0.02 (0.00, 0.11)	Leakage as reason to stop cup use	3 cycles

Note: Past and current menstrual cups. In the study by Cheng et al. (1995), a vaginal cup with a valve in the stem was used.[30] Leakage during menstrual cup use was reported in various ways; eleven studies with twelve data points provided information (1264 participants)

Table S10. Use of menstrual cup, product related discontinuation and other loss to follow up

Study	Cup brand (type)	Follow up time with cup use	Could not insert cup n/N (%)	Used cup at least once (verbal report documented) n/N (%)	Cup-related discontinuation n/N (%)§	Loss to follow up for other (or unclear) reasons n/N (%)
APHCR 2010[24] (girls)	Mooncup (vaginal)	3 cycles	6/60 (10.0)	49/60 (81.7)	14/60 (23.3)	5/60 (8.3)
APHCR 2010[24] (women)	Mooncup (vaginal)	3 cycles	2/36 (5.6)	33/36 (91.7)	3/36 (8.3)	1/36 (2.8)
Beksinska 2016[13]	Mpower (vaginal)	3 cycles	NR	99/110 (90.0)	NR	5/110 (4.5)
Care International 2018[43]	Ruby cup (vaginal)	3 cycles	NR	84/100 (84.0)	NR	NR
Cattanach 1991[29]	Gynaeseal (cervical)	18 months	7/80 (8.8) ‖	73/80 (91.3) ‖	NR	NR
Cheng 1995[30]	Menses Cup, Shanghai (vaginal)	1-13 months (22/51 [43%] stopped after c1)	0/51 (0.0)	51/51 (100.0)	43/51 (84.3)	NR
Chintan 2017[31]	Flowcare (vaginal)	8 weeks	NR	NR	NR	NR
Femme International[2]† (girls site 1)	Ruby cup (vaginal)	6 months	12/113 (10.6)	73/113 (64.6)	NR	NR
Femme International[2]† (girls site 2)	Ruby cup (vaginal)	12 months	NR	24/31 (77.4)	NR	NR
Femme International[2]† (girls site 3)	Ruby cup (vaginal)	6 months	NR	17/40 (43.6)	NR	NR
Femme International[2]† (women site 1)	Ruby cup (vaginal)	12 months	NR	2/60 (3.3)	NR	NR
Femme International[2]† (women site 2)	Ruby cup (vaginal)	6 months	NR	23/41 (56.1)	NR	NR
Gleeson 1993[34]	Gynaeseal (cervical)	1 cycle	1/22 (4.5)	21/22 (95.5)	NR	NR
Hoffmann 2014[19]*	NR (vaginal)	8 months	NR	2 months:<br>random 57/174 (32.8)<br>choice 22/46 (47.8)	NR	2 months:<br>random 21/174 (12.1)<br>choice 0/46 (0.0)
Howard 2011[12]	Divacup (vaginal)	3 cycles	NR	54/56 (96.4)	1/56 (1.8)	8/56 (14.3)
Kakani 2017[35]	NR (vaginal)	3 cycles	2/158 (1.3)	156/158 (98.7)	6/158 (3.8)	4/158 (2.5)
Madziyire 2018[3]	Butterfly cup (vaginal)	3 cycles	0/54 (0.0)	54/54 (100.0)	1/54 (1.9) Husband refused	1/54 (1.9)
North 2011[14]	Softcup (cervical)	3 cycles	4/406 (1.0)	368/406 (90.6)	24/406 (5.9)	74/406 (18.2)
Oster 2009 (report 14853)[20]	Mooncup (vaginal)	15 months	NR	59/98 (60.2)	1/98 (1.0)	0/98 (0.0)
Parker 1966[37]	Tassette (vaginal)	2-6 months	NR	46/46 (100.0)	NR	NR
Pena 1962[38]	Tassette (vaginal)	3 cycles	0/125 (0.0)	125/125 (100.0)	0/125 (0.0)	0/125
van Eijk 2018[52] (part of Phillips-Howard 2016[1])	Mooncup (vaginal)	median 7.4 months, range 1-14	NR	187/229 (81.7)	NR	41/229 (17.9)
Shihata 2014[39]	FemmyCycle (vaginal)	3 cycles	6/146 (4.1)	125/146 (85.6)	36/146 (24.7)	5/146 (3.4)
Stewart 2010[40]	Mooncup (vaginal)	3 cycles	NR	26/54 (48.1)	NR	33/54 (61.1)
Tellier 2012[41]	Ruby cup (vaginal)	3-5 cycles	NR	15/31 (48.4)	NR	16/31 (51.6)
**Pooled prevalence‡, 95% CI<br>I^2, number of studies<br>Total participants**			**2.8%, 0.8-5.6%, I^2 79.3%, n=11, 1251 participants**	**79.3%, 68.5-88.4%, I^2 97.1%, n=25, 2367 participants**	**10.2%, 2.7-22.6%, I^2 96.4%, n=10, 1190 participants**	**9.0%, 3.8-15.9%, I^2 94.9%, n=15, 1783 participants**

NR=not reported.

* Cross-over of menstrual cup and pad users possible after 2 months in the study. Random: these participants were randomized to receive the menstrual cups. Choice: these women obtained the menstrual cup by choice.

† Monitoring and evaluation of distribution program in Tanzania in Kilimanjaro region;[2] 2 girls were not allowed a menstrual cup and 5 girls reported to have lost the menstrual cup

‡ For forest plots see Figures S7-S10

§ Reasons for cup-related discontinuation: Cheng 1995: (cup with drainage tube that can be unplugged) too long (29%), difficult to insert or position (25%), uncomfortable (25%), messy to unplug (17%), difficult to use during light flow (4%). Femme international: main reasons fear, difficulty inserting and pain.[30] Kakani 2017: feeling messy (2 women), could not insert cup (2), difficult removal (2).[35] North 2011: poor fit (11 women), messy (4), cramping (4), difficult removing (4), didn't like it (3).[14] Oster 2009: one girl did not like the MC.[20] Shihata 2014: (no numbers available): device too big, variable degrees of prolapse of cervix, not able or wanting to insert.[39]

‖ 80 responders among 259 women approached by (slow) mail after 18 months.[29]

Table S11. Acceptability of the menstrual cup among women who tried the MC

Study	Cup brand (type)	Follow up time (cup use)	Difficult to insert (%)	Uncomfortable to wear (%)	Difficult to remove (%)	Overall evaluation
APHCR 2010[24] (girls)	Mooncup (vaginal)	3 cycles	14/49 (28.6)	3/49 (6.1)	NR	NR
APHCR 2010[24] (women)	Mooncup (vaginal)	3 cycles	2/33 (6.1)	2/33 (6.1)	NR	NR
Beksinska 2016[13]	Mpower (vaginal)	3 cycles	4/106 (3.8) c3	21/106 (19.8) c3	4/106 (3.8) c3	97% cup better than other methods, 3% cup worse
Care International 2018[27]	Ruby cup (vaginal)	3 cycles	NR	NR	NR	15/16 (93.8%) very satisfied
Cattanach 1991[29]	Gynaeseal (cervical)	18 months	13/73 (17.8)	NR	12/73 (16.4)	Compared to tampons: 31/62 (50.0%) thought it better, 9/62 (14.5%) thought it worse
Cheng 1995[30]	Menses Cup, Shanghai (vaginal)	1-13 months (22/51 or 43% stopped after c1)	23/51 (45.1) c1	32/51 (62.7) c1	0/51 (0.0) c3	18/51 (35%) thought cup acceptable
Chintan 2017[31]	Flowcare (vaginal)	8 weeks	65/100 (65.0) c1	NR	NR	43% cup better, 21% previous item better
Femme International[2]† (girls site 1)	Ruby cup (vaginal)	6 months	13/113 (11.5)	NR	NR	NR
Femme International[2]† (girls site 2)	Ruby cup (vaginal)	12 months	2/31 (6.5)	NR	NR	NR
Femme International[2]† (girls site 3)	Ruby cup (vaginal)	6 months	8/40 (20.0)	NR	NR	NR
Femme International[2]† (women site 1)	Ruby cup (vaginal)	12 months	5/7 (71.4)	NR	NR	NR
Femme International[2]† (women site 2)	Ruby cup (vaginal)	6 months	2/29 (6.9)	NR	NR	NR
Gleeson 1993[34]	Gynaeseal (cervical)	1 cycle	1/22 (4.5) c1	NR	1/21 (4.8)	NR
Hoffmann 2014[19]	NR (vaginal)	8 months	NR	randomized: 11/151 (7.3) 8 months<br>choice: 1/46 (2.2) 2 months	NR	Preference of cup to baseline method:<br>2 months 43/153 (28.1),<br>6 months 59/144 (41.0)
Howard 2011[12]	Divacup (vaginal)	3 cycles	Insertion: Likert scale 1-7*: cup 4.9 vs. tampon 5.2	Wear: Likert scale 1-7*: cup 5.5 vs. tampon 5.3<br><br>vaginal discomfort ≥1 day in c1-c3: cup 23/45 (51.1%) vs. tampon 12/44 (27.3%), p=0.02 cycle 3: 7/45 for cup	Removal: Likert scale 1-7*: cup 5.0 sd 1.4, vs. tampon 5.5 sd 1.1, p=0.06	Mean satisfaction score: Likert scale 1-7*: cup 5.4 sd 1.5 vs. tampon 5.0, sd 1.0, p=0.04
Kakani 2017[35]	NR (vaginal)	3 cycles	15/150 (10.0) c3	10/150 (6.7) c3<br>Dryness: 5/150 (3.3)	8/150 (5.3)	NR
Madziyire 2018[3]	NR (vaginal)	3 cycles	10/54 (0.0) c1, 1/53 in c2, 0/52 in c3	10/54 c1, 2/53 c2, 0/52 (0.0) c3	NR	52/54 willing to use after c3
North 2011[14]	Softcup (cervical)		average of 8†	average of 8†	average of 8†	~37% cup better than previous methods, 29% cup worse after c3‡
North 2011[14] Manufacturer database 2003-2008	Softcup (cervical)	Unknown. Estimate manufacturer: 1 complaint/47,000 cups sold	104 (no denominator)	67 (moves when worn)<br>(no denominator)	108 (no denominator)	NA
Oster 2011[22]	Mooncup (vaginal)	15 months	NR	NR	NR	NR
Parker 1966[37]	Tassette (vaginal)	2-6 months	22/45 (48.9)	19/45 (42.2)	9/45 (20.0) "messy"	23/45 (51.1) preferred cup to previous method
Pena 1962[38]	Tassette (vagina)	3 cycles	NR	6/125 (4.8) slight discomfort in beginning	NR	NR
Van Eijk 2018[52]	Mooncup (vaginal)	median 7.4 months, range 1-14	<3 months: 27/143 (18.9), 3-5 months: 13/143 (9.1)**,<br>6-8 months: 7/104 (6.7), 9+ months: 2/74 (2.7)	NR	NR	NR
Shihata 2014[39]§	FemmyCycle (vaginal)	3 cycles	NR	17/105 (16.2)	NR	88/105 (83.8) satisfied with menstrual cup
Stewart 2010[40]	Mooncup (vaginal)	3 cycles	NR	NR	NR	NR

Tellier 2012[41]	Ruby cup (vaginal)	3-5 cycles	5/15 (33.3)	NR	7/15 (46.7)	NR
**Pooled prevalence, 95% CI, ††** **I^2, number of studies,** **total of participants**			**20.3%, 11.7-30.4%,** **I^2 92.3%, n=17,** **1061 participants**	**12.6%, 5.9-21.3%,** **I^2 91.9%, n=12,** **958 participants**	**9.3%, 2.9-18.3%** **I^2 84.7%, n=7,** **461 participants**	

Abbreviations: c1=cycle 1 (first menstrual period with cup use). c2=menstrual cycle 2. c3=menstrual cycle 3. NA=not applicable. NR=not reported. Sd=standard deviation.
* Scale of -7 with 1 as terrible and 7 as terrific
† Scale of 1-10 with 1 as poor and 10 as great
‡ Estimated from Figure 2 in publication, no numbers available
§14 women were removed from initial sample after cycle 2 because of leaking problems due to variable degrees of prolapse or low cervix
** This information was used in meta-analysis
†† For forest plots see S11-S13

Table S12. Information on mobility and odour when using a menstrual cup

Study	Menstrual Cup type	Follow up time (cup use)	Improved Mobility	Less Odour
North 2011[14]	Softcup (cervical)	3 cycles	~51% cup better than previous method, 3% cup worse (c3)§	~41% cup better than previous method, 12% cup worse (c3) §
Oster 2012[23]	Mooncup (vaginal)	15 months	convenient for mobility 14/97 (14.0)	NR
Parker 1966[37]	Tassette (vaginal)	2-6 months	35/45 (77.8)	Absent odour in 42/46 (91.3)
Pena 1962[38]	Tassette (vaginal)	3 cycles	NR	"often reported" (No numbers)
Shihata 2014[39]*	FemmyCycle (vaginal)	3 cycles	101/105 (96.2) excellent mobility	97/105 (92.4) excellent odour prevention

*First author of Shihata 2014 is patent holder on FemmyCycle[53]
NR – not reported

Figure S2. Proportion of women who reported they could not insert the menstrual cup, studies between 1960 and 2017

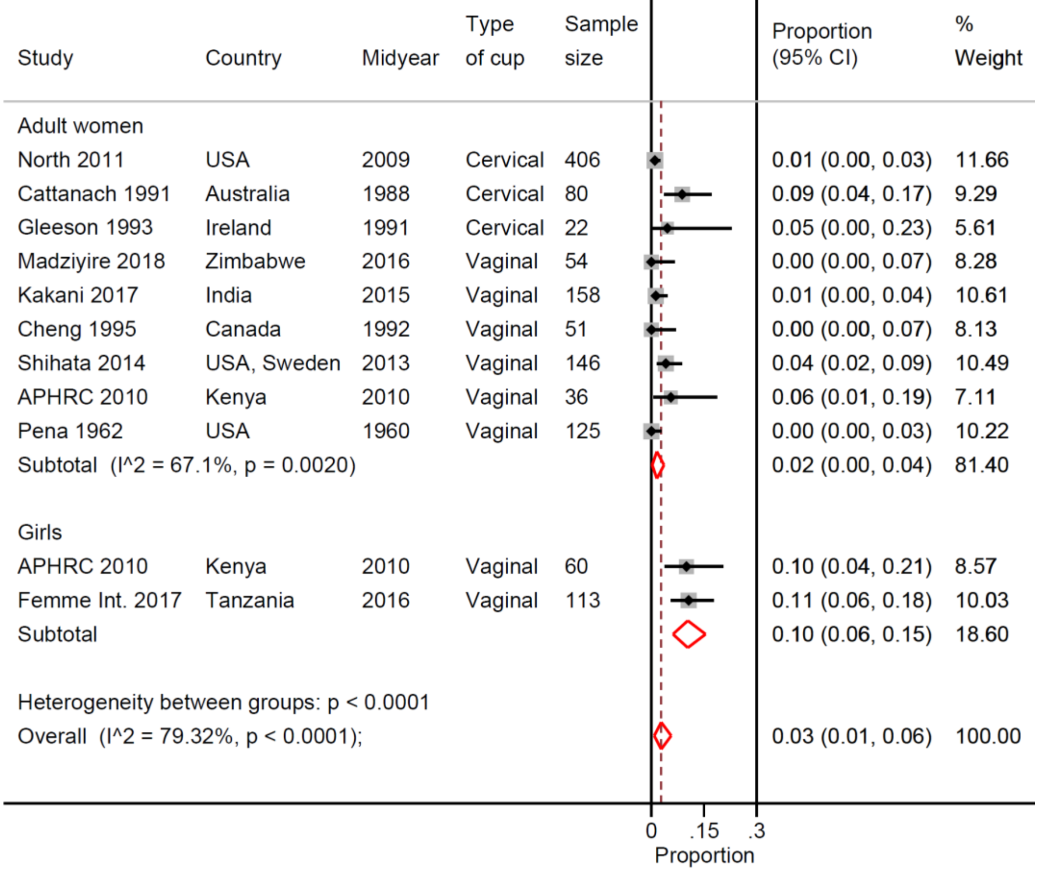

Figure S3. Proportion of women who reported they used the menstrual cup at least once, studies between 1960 and 2017

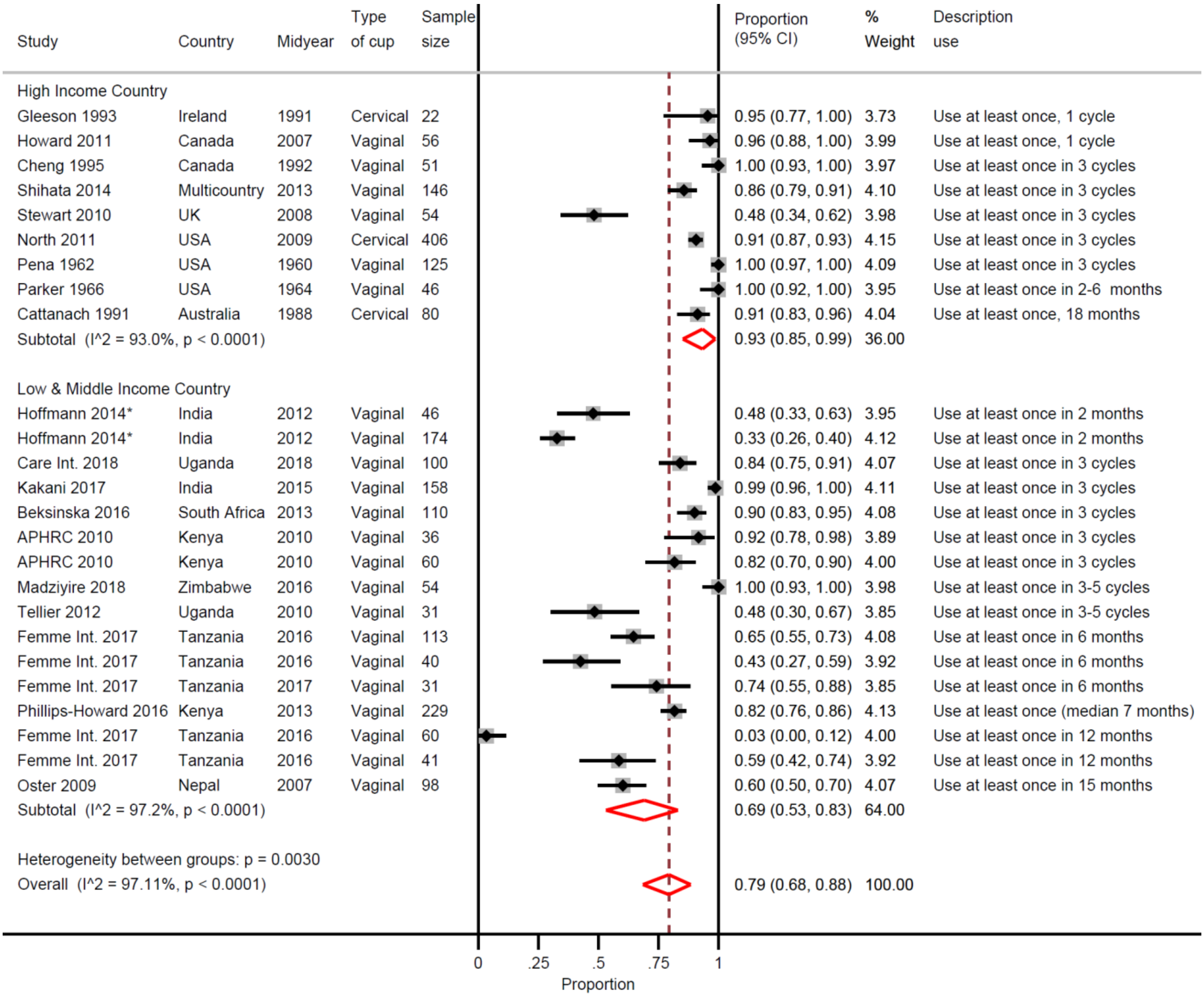

Cattanach 1991: 80 women who responded among 259 approached women by slow mail after 18 months[29]
*Hoffmann 2014: n=46: women who got MC by choice, n=174: women who received MC by randomization[19]

Figure S4. Menstrual cup related discontinuation, studies between 1960 and 2017

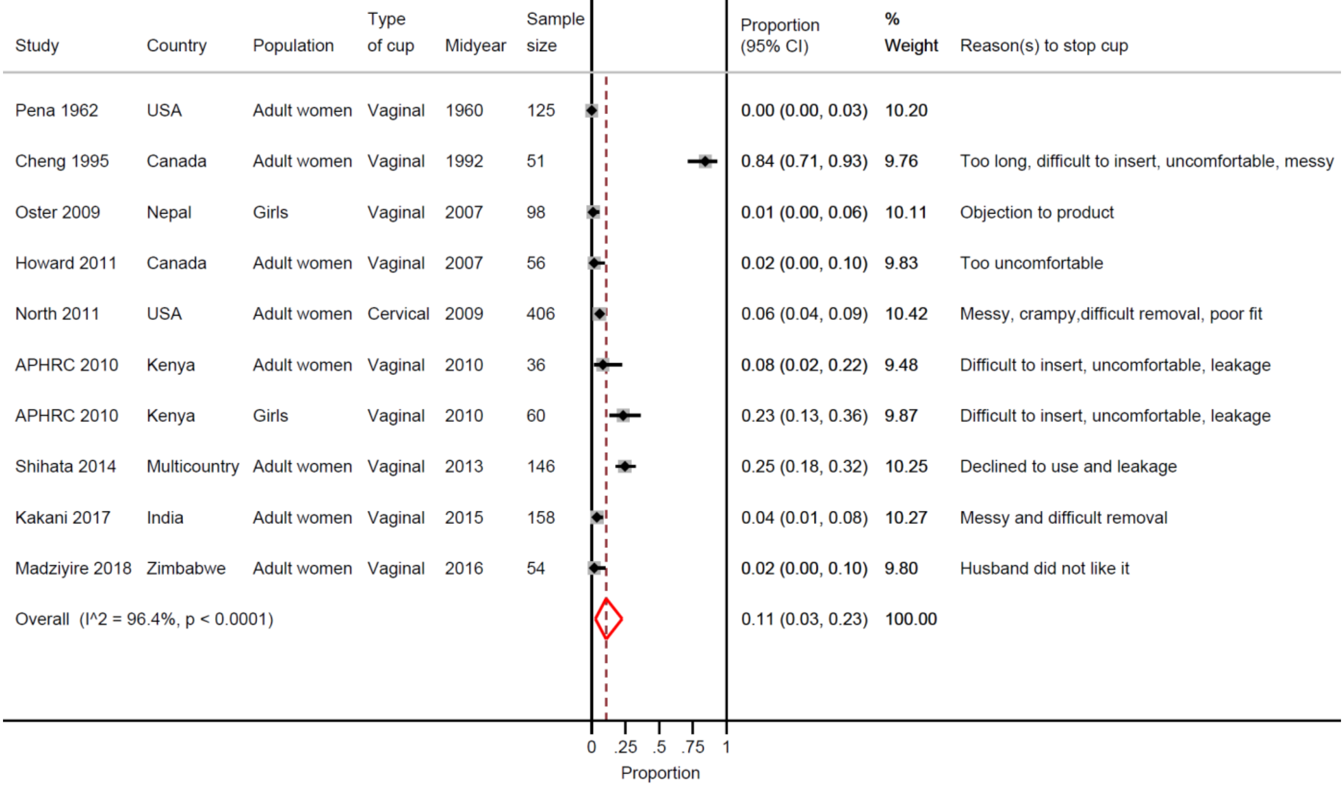

Figure S5. Other reasons for discontinuing the menstrual cup, studies between 1960 and 2017

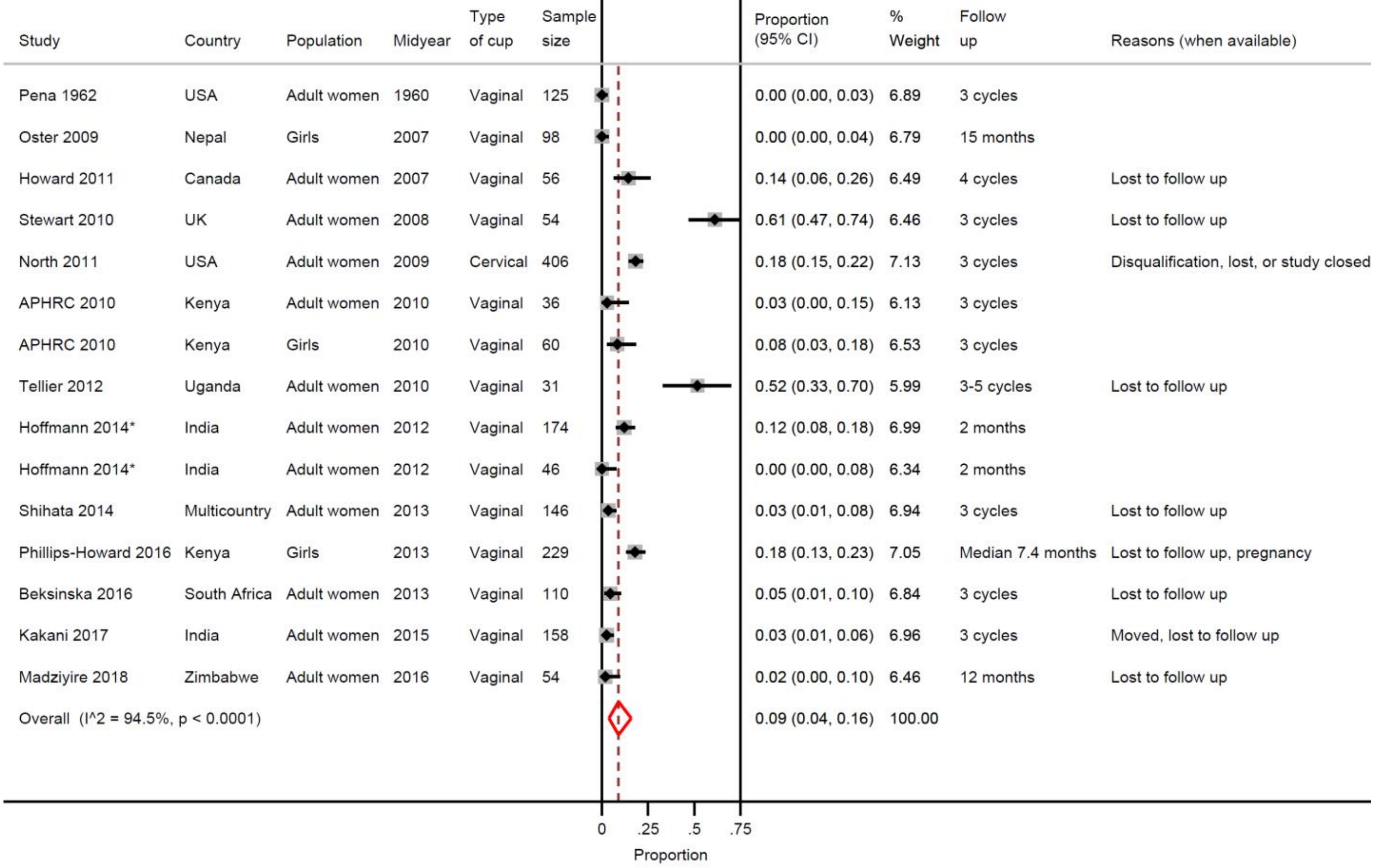

*Hofmann 2014: 174 women received the cup by randomization, and 46 by choice.[19] North 2011: 23 women who were enrolled but did not qualify (e.g. abnormal laboratory results) were excluded from the analysis. Fifty-one women were not included for other reasons (forms lost, study closure, lost to follow up etc).[14]

Figure S6. Reported difficulty with insertion of the menstrual cup by time point of evaluation, 1960-2017

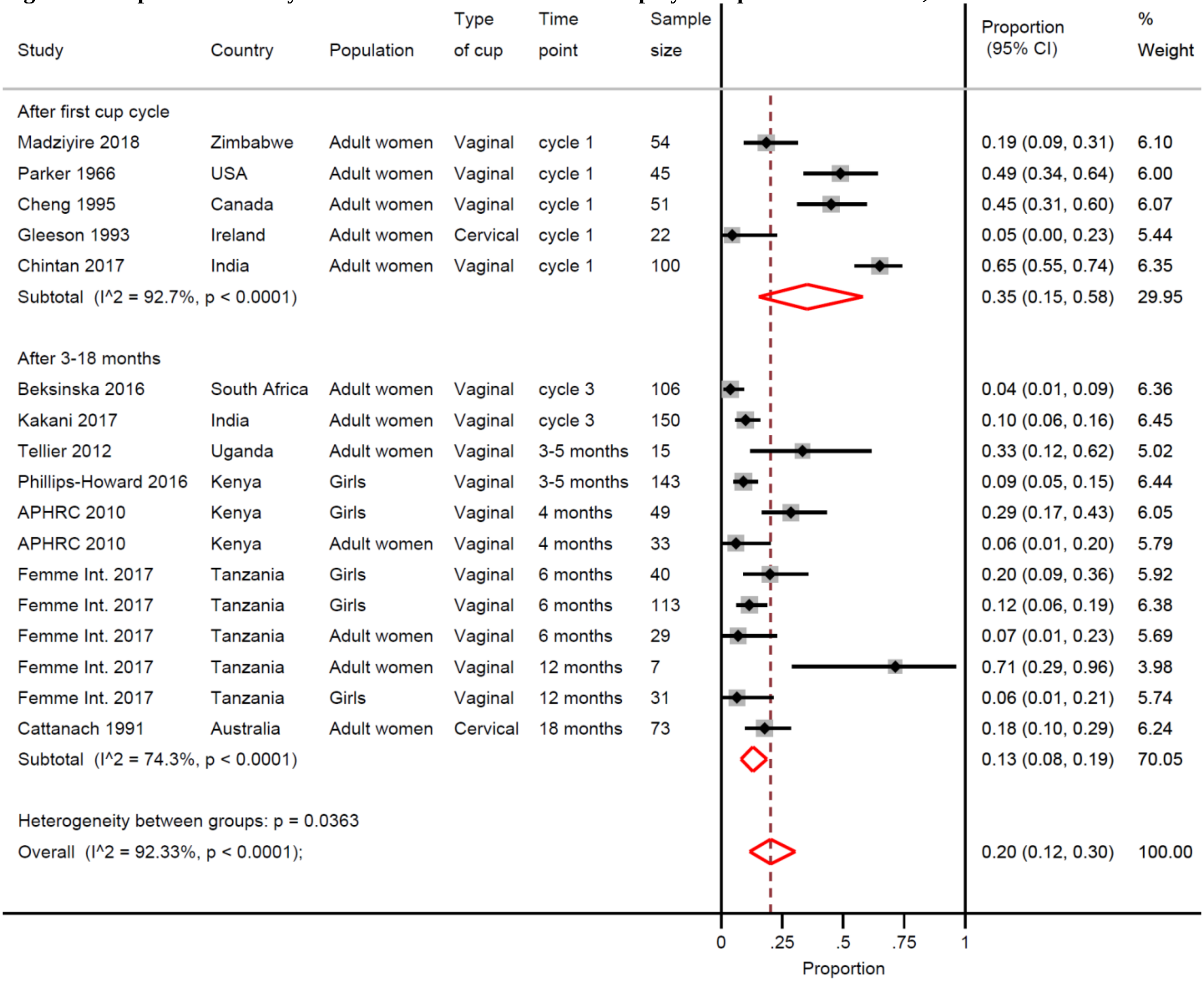

The p-value for “time point of evaluation” (graph above) was 0.15.

Figure S7. Discomfort when wearing the menstrual cup, studies between 1960 and 2017 by time point of evaluation

Study	Country	Population	Time point	Sample size	Proportion (95% CI)	% Weight
After first cup cycle						
Parker 1966	USA	Adult women	cycle 1	45	0.42 (0.28, 0.58)	8.03
Cheng 1995	Canada	Adult women	cycle 1	51	0.63 (0.48, 0.76)	8.15
Pena 1962	USA	Adult women	cycle 1	125	0.05 (0.02, 0.10)	8.76
Subtotal (I^2 = 97.5%, p < 0.0001)					0.33 (0.02, 0.76)	24.94
After 2-18 months						
Hoffmann 2014*	India	Adult women	2 months	46	0.02 (0.00, 0.12)	8.05
Shihata 2014	USA, Sweden	Adult women	3 months	105	0.16 (0.10, 0.25)	8.68
APHRC 2010	Kenya	Adult women	4 months	33	0.06 (0.01, 0.20)	7.66
APHRC 2010	Kenya	Girls	4 months	49	0.06 (0.01, 0.17)	8.11
Hoffmann 2014*	India	Adult women	8 months	151	0.07 (0.04, 0.13)	8.84
Madziyire 2018	Zimbabwe	Adult women	cycle 3	52	0.00 (0.00, 0.07)	8.17
Howard 2011	Canada	Adult women	cycle 3	45	0.16 (0.06, 0.29)	8.03
Kakani 2017	India	Adult women	cycle 3	150	0.07 (0.03, 0.12)	8.84
Beksinska 2016	South Africa	Adult women	cycle 3	106	0.20 (0.13, 0.29)	8.68
Subtotal (I^2 = 77.1%, p < 0.0001)					0.08 (0.04, 0.13)	75.06
Heterogeneity between groups: p = 0.59						
Overall (I^2 = 91.90%, p < 0.0001)					0.13 (0.06, 0.21)	100.00

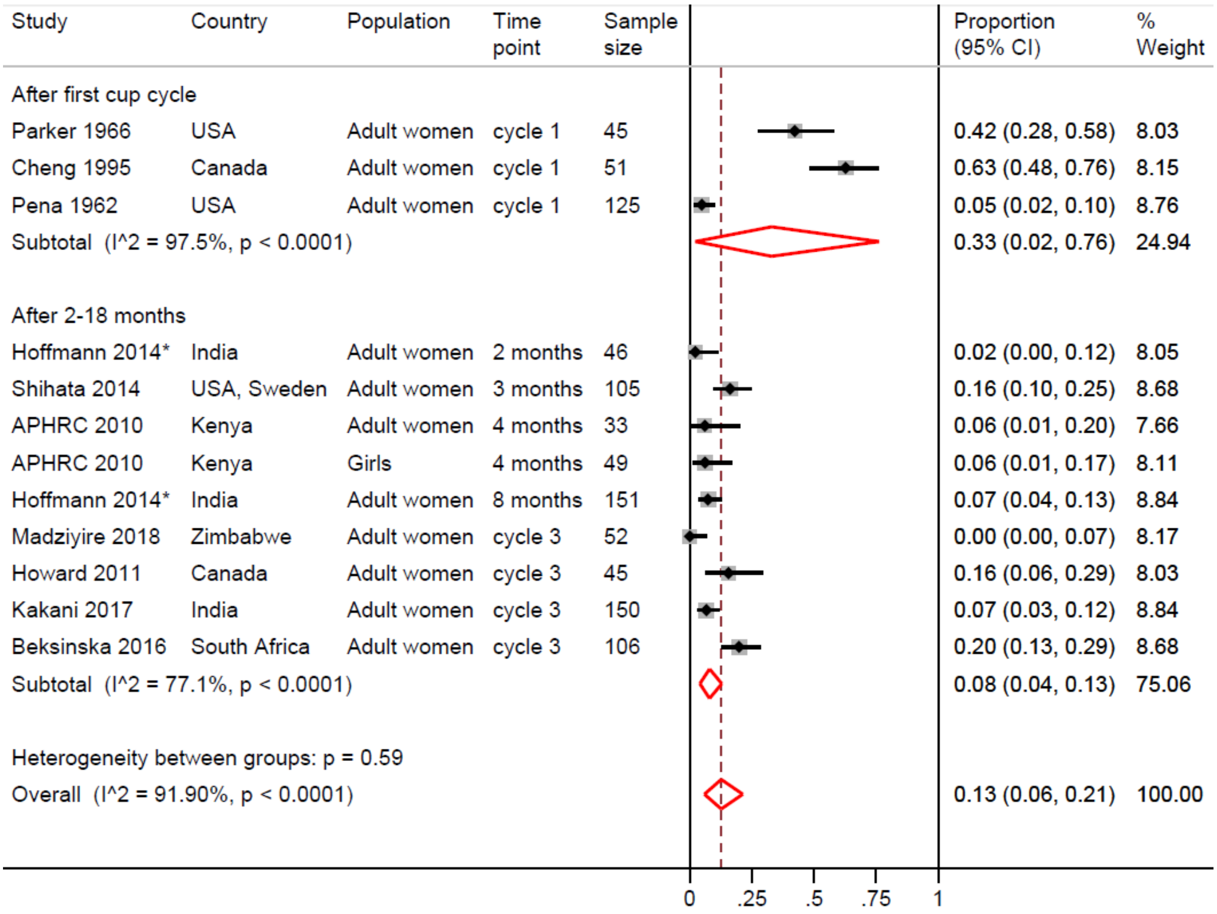

Note: All vaginal cups
*151 women received the cup by randomization, and 46 by choice

Figure S8. Difficulty removing the menstrual cup, studies between 1960 and 2017

Study	Country	Population	Type of cup	Time point	Sample size	Proportion (95% CI)	% Weight
Gleeson 1993	Ireland	Adult women	Cervical	cycle 1	21	0.05 (0.00, 0.24)	11.83
Cheng 1995	Canada	Adult women	Vaginal	cycle 3	51	0.00 (0.00, 0.07)	14.76
Beksinska 2016	South Africa	Adult women	Vaginal	cycle 3	106	0.04 (0.01, 0.09)	16.25
Kakani 2017	India	Adult women	Vaginal	cycle 3	150	0.05 (0.02, 0.10)	16.71
Tellier 2012	Uganda	Adult women	Vaginal	3-5 months	15	0.47 (0.21, 0.73)	10.45
Parker 1966	USA	Adult women	Vaginal	3-6 months	45	0.20 (0.10, 0.35)	14.42
Cattanach 1991	Australia	Adult women	Cervical	18 months	73	0.16 (0.09, 0.27)	15.59
Overall (I^2 = 84.7%, p < 0.0001)						0.09 (0.03, 0.18)	100.00

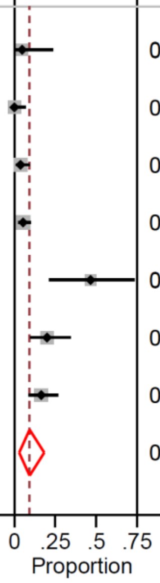

Figure S9. Time trends for menstrual cup use in four studies with information from developing countries

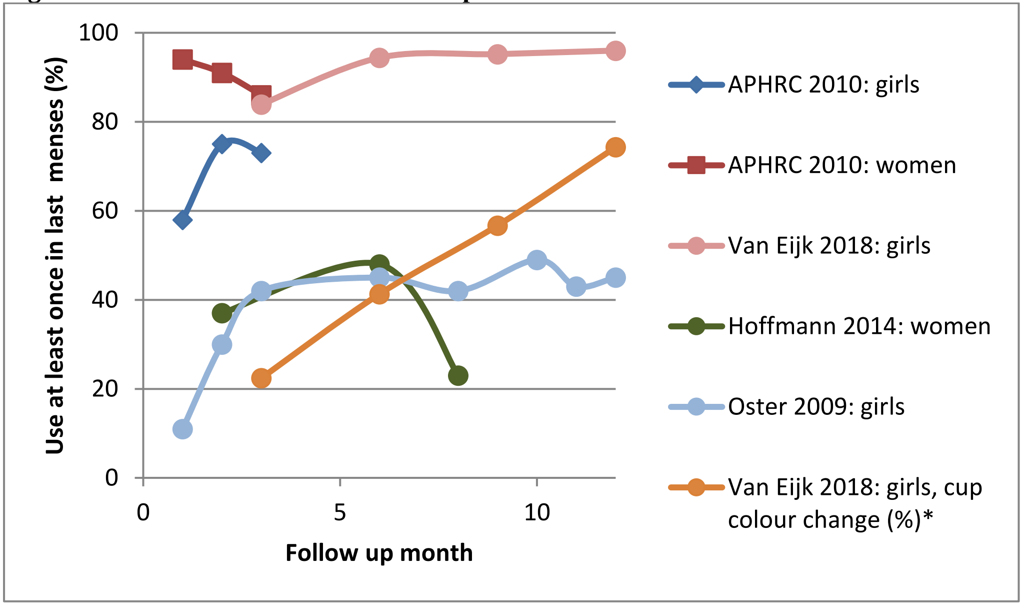

Note: In the study by Hoffman et al. (2014)[19], there was an option to obtain pads among women randomized to menstrual cups from 2 months onward. In both studies by van Eijk et al. (2018)[52] and Oster et al. (2009)[20], there was regular contact between study staff and participants. In the study by van Eijk et al. (2018)[52] peer support was also provided. Although peer education was absent in the study by Oster et al. (2009),[21] they do show that friends who use the cup are important for successful uptake.

*For comparison, the cup colour change over time is included, as reported by van Eijk et al. (2018).[52] In this study, mooncups were used, which show a discolouration after use. This may be considered as a more objective measure of use compared to verbal report and may better illustrate the learning curve for cups. Note that this is aggregated data per quartile. For information by month, see the article.

Figure S10. Sensitivity analyses, funnel plots and small-study effect

Sentivity analyses results

Outcome	Number of high-quality studies or subgroups	Number of low-to-moderate quality studies or subgroups	p-value for subgroup difference
Could not insert cup	0	11	
Use MC at least once (verbal report)	0	25	
MC-related discontinuation	0	10	
Other discontinuations	2	13	1.00
Difficult to insert	2	15	0.11
Uncomfortable to wear	1	11	0.55
Difficult to remove	1	6	0.30
Wants to continue using the cup	1	14	0.53

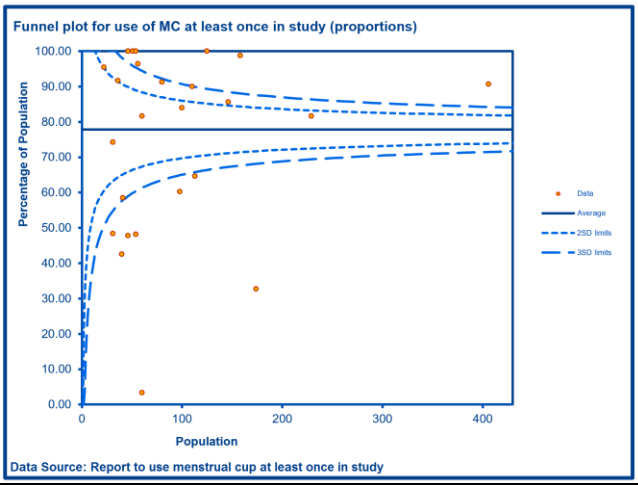

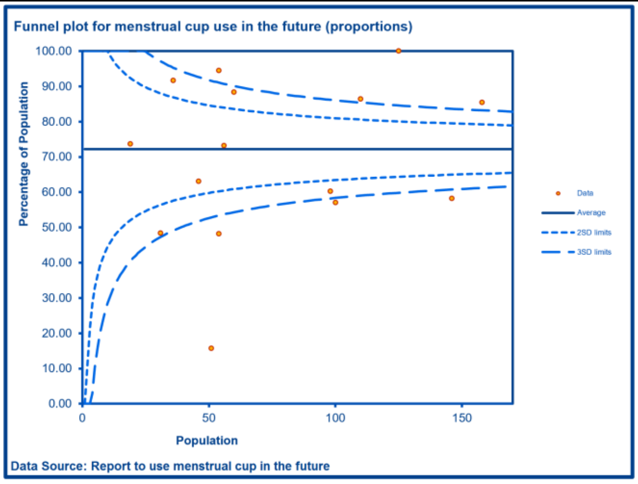

SD=standard deviation. MC=menstrual cup.

Notes: The control limits or 'funnels' represent the bounds of statistical confidence around the average value.[6] Specifically, this means that any observation plotted within the funnel limits will have a confidence interval which includes the average value. Conversely, the confidence interval of any observation plotted outside the funnel limits would not include the average value, and may therefore indicate unexpected deviation from the norm.[6] It is clear in these plots that most of our data points fall outside of the 2SD limits. Asymmetry in funnel plots suggests a potential for small-study effect with smaller studies (larger standard errors) showing greater treatment effects; most of our studies have small (<100) sample sizes; given the lack of studies with larger sample sizes and the wide variety in study populations and outcome measures used, it may not be possible to assess this effect properly. The two-sided p-values for asymmetry of the funnel plots by Egger's test were 0.353 for use of MC at least once and 0.366 for future use, providing no evidence for the presence of small-study effects.

For the other outcomes examined the Egger's test is presented below:

Outcome	Number of studies	p-value Egger's test
Could not insert cup	8	0.041
MC-related discontinuation	9	0.86
Other discontinuations	12	0.54
Difficult to insert	18	0.46
Uncomfortable to wear	11	0.48
Difficult to remove	6	0.22

Table S13. Other outcomes examined in association with menstrual cup use

Study	Study type	Comparison	Outcome	Menstrual Cup	Alternative	P-value
Phillips-Howard 2016[1] *	Cluster randomized trial Kenya	Vaginal cup vs. pad vs. usual item	Drop out of school – all users***	11.2%	pads 10.2%; usual item 8.0%	>0.05
Phillips-Howard 2016[1] *	Cluster randomized trial Kenya	Vaginal cup vs. pad vs. usual item	Sexually transmitted infections<br>- chlamydia<br>- gonorrhoea<br>- trichomoniasis	4.2%<br>2.1%<br>0.7%<br>1.4%	Pads: 4.5%; Control: 7.7%<br>Pads: 1.5%; Control: 4.5%<br>Pads: 0.5%; Control: 0.6%<br>Pads: 2.5%; Control: 4.5%	0.039†
Phillips-Howard 2016[1] *	Cluster randomized trial Kenya	Vaginal cup vs. pad vs. usual item	Reproductive tract infections<br>- bacterial vaginosis<br>- *Candida albicans*	21.5%<br>14.6%<br>7.7%	Pads: 28.7%; Control: 26.9%<br>Pads: 19.8%; Control: 20.5%<br>Pads: 9.5%; Control: 8.3%	>0.05
APHRC 2010[25]	Before-after study Kenya	Vaginal cup vs. usual item	1) School attendance<br>2) School concentration	1) 20% (11/55)<br>2) 29% (16/55)	1) 27% (16/60)<br>2) 47% (28/60)	NR
Oster 2009[20,22]	Individually randomized trial Nepal	Vaginal cup vs. usual item	School attendance			>0.05
Oster 2009[20,22]	Individually randomized trial Nepal	Vaginal cup vs. usual item	Daily time doing laundry during menstrual period			<0.05
Hoffman 2014[19] ‡	Cluster randomized trial India	Vaginal cup vs. pads	Uptake of low and high barrier menstrual item*	37% 2 months<br>50% 6 months<br>30% 8 months	87% 2 months<br><br>97% 8 months	<0.05
Stewart 2010[40]	Before-after study, UK	Vaginal cup vs. usual product (tampon or pad)	Average number of times changed per cycle	14.8	17.6	NR
Femme International 2017[2]	Observational, menstrual item by choice Tanzania	Vaginal cup vs. reusable pad	Uptake of use of product	65.0%<br>6 months	95.7%<br>6 months	<0.05

NR: Not reported.
* When data were stratified by duration of use, participants provided with cups for 9 months or 12 months and longer had significantly lower prevalence of school dropout, STI, and BV
† p value for cups v control for sexually transmitted infections combined, adjusted for covariates
‡ Hoffman 2014[19]: Assumed 11.9% loss to follow up at 2 months as reported by authors. In this trial with an elaborate design there was ability to cross-over in some clusters at 2 and 6 months: “Usage of menstrual cups fell off sharply when these women were later given pads, suggesting that many women prefer pads, but will use the cup if pads are not available or affordable”.

Section 2.3 Visibility, availability, costs and waste

Visibility of MCs

Three studies in HIC with information on potential use of a MC suggested MCs were not well known, with only 11-33% of the persons interviewed aware,[44-46] although interest in a fourth study in Zimbabwe was considerable (Table S14).[43] We identified 69 websites from 27 countries and one international organization with educational or teaching materials to guide adolescent girls through menarche (Table S15); disposable pads, tampons, MCs, and reusable pads were reported by 76·8%, 65·2%, 30.4 and 21.7%, respectively, with some regional differences (Figure S16).

Table S14. Evaluation of potential use of menstrual cup

Study	Location	Time	Design	Sample size and population	Menstrual cup	Outcome examined	Results	Comments
Averbach 2009[43]	Epworth, Zimbabwe	2007-2008	Survey & FGD	43 adult women, 18-45 years, convenience sample from public clinics, markets and shopping centers	Duet (cervical cup, re-usable)	Consider use (Duet demonstrated but not tried by participants)	100% would try Duet for menstrual protection<br>76.7% would not be concerned if used for both menstruation and contraception<br>86.1% thinks it would make a difference to their lives<br>100% think the low cost and easiness to clean are important for trying<br>30% concerns about Duet getting lost inside	"Women felt that Duet potentially addressed the washing, drying and other hygiene issues they had with home products, as it is easy to wash with a small amount of water and soap, it can be immediately dried and reinserted, and thus is discreet."
Stewart 2009[46]	Nottingham, UK	NR	Survey	69 patients attending a menstrual disorder clinic	Mooncup	Acceptability after reading information leaflet	20% had heard of cup. 38% would consider cup in future. One woman already used it. 69% mentioned perceived messiness as reason against considering mooncup, and 51% the need for washing between uses.	Higher potential use by age:<br><30 years 3/18 (16.7%)<br>30-40 years 8/21 (38.1%)<br>>40 years 15/30 (50.0%)<br>p = 0.04 (Fisher), <30 vs. >40<br>"Women who used tampons and described their menstrual flow as average to heavy were more likely to consider…" (Data not presented)
Grose 2014[45]	California, USA	NR	Survey: evaluation of water bottle (dummy) and menstrual cup by participants	151 female undergraduates	No particular brand	The relationship between self-objectification and attitudes toward an alternative menstrual product	33% knew menstrual cup. On scale 1-5 (1 = yes, 5 is no), mean 2.5 if asked if they would use the menstrual cup if it was given to them. 6% would use and 15 (9.9%) probably would use.	"..higher levels of self-objectification were related to more negative attitudes toward one's menstruation, which was, in turn, negatively related to women's own probable purchase or use of the product, as well as overall negative reactions to it."
Borowski 2011[44]	USA	2011	Survey and key person interviews	155 adult women, selected group related to author (80% white)	No particular brand	Awareness of environmental and health issues of menstrual products	69% not aware of eco-friendly feminine hygiene products<br>Among 47/150 (31.3%) who were aware of eco-friendly products, 18 mentioned menstrual cups (38.3% or 11.6% overall).<br>Advertisement and main stream marketing was considered important by participants for scaling up eco-friendly products	"The thing that keeps me from considering reusable products is the need to have to either emerge from a public toilet stall with blood on one's hands/a used cup or carry a soiled pad around in one's purse after use. I'm not sure how to solve these problems."

Abbreviations: FGD=Focus groups discussions.

Table S15. Menstrual items reported in education material for girls

Country	Website	Date accessed	Language	Disposable pads	Tampons	Menstrual Cup	Reusable pads
Argentina	http://www1.paho.org/hq/dmdocuments/2010/Recomendaciones-atencion-integral-salud-adolescentes-salud-sexual-reproductiva.pdf	21 May 2018	Spanish	Yes	Yes	No	No
Australia	https://www.betterhealth.vic.gov.au/health/conditionsandtreatments/menstrual-cycle	25 May 2019	English	Yes	Yes	No	No
Australia	http://www.cyh.com/HealthTopics/HealthTopicDetailsKids.aspx?p=335&np=289&id=1817#12	25 May 2019	English	Yes	Yes	No	No
Australia	https://www.healthdirect.gov.au/menstruation	25 May 2019	English	No	No	No	No
Australia	https://jeanhailes.org.au/health-a-z/periods/about-the-menstrual-cycle	25 May 2019	English	Yes	Yes	Yes	Yes
Australia	http://raisingchildren.net.au/articles/periods.html#sanitary	25 May 2019	English	Yes	Yes	No	No
Brazil	http://www.adolescencia.org.br/site-pt-br/corpo-da-menina	23 May 2018	Portuguese	No	No	No	No
Brazil	https://brasilescola.uol.com.br/saude/coletor-menstrual.htm	26 May 2018	Portuguese	No	No	Yes	No
Brazil	http://bvsms.saude.gov.br/bvs/folder/10006002035.pdf	21 May 2018	Portuguese	Yes	No	No	No
Brazil	http://bvsms.saude.gov.br/bvs/publicacoes/caderneta_saude_adolescente_menina.pdf	23 May 2018	Portuguese	No	No	No	No
Brazil	https://www.mdsaude.com/ginecologia/menstruacao/primeira-menstruacao-menarca (medical blog)	26 May 2018	Portuguese Spanish	Yes	Yes	No	No
Canada	http://www.canadianliving.com/health/nutrition/article/stages-of-puberty	25 May 2019	English	Yes	Yes	No	No
Canada	https://www.caringforkids.cps.ca/handouts/information_for_girls_about_puberty	25 May 2019	English/ French	No	No	No	No
Canada	http://www.cwhn.ca/en/node/44808	25 May 2019	English/ French	Yes	Yes	Yes	Yes
Canada	https://www.mamanpourlavie.com/sante/enfant/developpement/sexualite/4025-les-premieres-menstruations.thtml	25 May 2019	French	Yes	Yes	No	No
Canada	https://teachingsexualhealth.ca/teachers/wp-content/uploads/sites/4/2016/12/5-DA-My-Period-FRENCH-2014.pdf	25 May 2019	English/ French	Yes	Yes	Yes	No
Canada	https://www.yourperiod.ca/fr/normal-periods/your-first-period/	25 May 2019	English/ French	Yes	Yes	Yes	Yes
Chile	https://www.clinicalascondes.cl/BLOG/Listado/Pediatria/La-primera-menstruacion	20 May 2018	Spanish	Yes	No	No	No
Chile	http://www.codajic.org/sites/www.codajic.org/files/100-Preguntas-Sobre-Sexualidad-Adolescente.pdf	20 May 2018	Spanish	No	Yes	Yes	No
Chile	https://www.saludactual.cl/ginecologia/como-hablar-con-tu-hija-del-ciclo-menstrual.php	20 May 2018	Spanish	No	No	No	No
Colombia	https://www.minsalud.gov.co/Documentos%20y%20Publicaciones/Modelo%20de%20servicios%20de%20salud%20amigables%20para%20adolescentes%20y%20j%C3%B3venes.pdf	21 May 2018	Spanish	No	No	No	No
France	http://www.doctissimo.fr/html/sexualite/hygiene-feminine/articles/se_6684_regles_faq.htm	25 May 2019	French	Yes	Yes	Yes	No
France	http://www.doctissimo.fr/html/sexualite/hygiene-feminine/articles/se_7067_premieres_regles_panique.htm	25 May 2019	French	Yes	Yes	Yes	No
France	http://unesdoc.unesco.org/images/0022/002267/226792e.pdf	25 May 2019	French / English	Yes	Yes	Yes	Yes
France	https://www.youtube.com/watch?v=lFdneet53mg	25 May 2019	French	Yes	Yes	Yes	No
Germany	http://www.maedchen.de/love/periode-menstruation-infos#TamponOderBinde	25 May 2019	German	Yes	Yes	Yes	No
Germany	http://www.netdoktor.de/Gesund-Leben/Womens-Health/Menstruation/Die-erste-Regel-Menstruation-527.html	25 May 2019	German	Yes	Yes	No	No
Germany	https://www.profamilia.de/fileadmin/publikationen/Jugendliche/Broschuere_Menstruation_140203_ANSICHT.pdf †	25 May 2019	German	Yes	Yes	No	No
Germany	http://www.t-online.de/eltern/schulkind/id_18126782/menstruation-maedchen-auf-die-erste-blutung-vorbereiten.html	25 May 2019	German	Yes	Yes	No	No
Hong Kong	https://www.studenthealth.gov.hk/english/health/health_se/health_se_ps.html (for students)	25 May 2019	English	Yes	No	No	No
Hong Kong	https://www.studenthealth.gov.hk/english/health/health_se/health_se_pp.html (for parents)	25 May 2019	English	No	No	No	No
India	https://mdws.gov.in/menstrual-hygiene-management-national-guidelines-december-2015	25 May 2019	English	Yes	Yes	Yes	Yes
Malawi	https://www.unicef.org/wash/schools/files/UNICEF-MenstrualHygiene-PRINT-27May15.pdf	25 May 2019	English	Yes	No	Yes	Yes
Mexico	http://infogen.org.mx/menstruacion/	20 May 2018	Spanish	Yes	Yes	No	No
Mexico	http://www.saludnl.gob.mx/drupal/las-ni%C3%B1as-y-la-pubertad	20 May 2018	Spanish	No	No	No	No
Mozambique	https://www.medicusmundi.cat/wp-content/uploads/2016/09/Manual-Saude-Escolar_OK.pdf	26 May 2018	Portuguese	Yes	Yes	No	Yes
Netherlands	https://www.opvoeden.nl/puber/verzorging-en-veiligheid/lichaamsverzorging/maandverband-en-tampons/	25 May 2019	Dutch	Yes	Yes	No	No
Netherlands	https://rutgers.media/pubergids/ongesteld-worden/	25 May 2019	Dutch	Yes	Yes	No	No
Netherlands	https://www.seksualiteit.be/voorlichting/puberteit-en-lichaam/de-eerste-menstruatie	25 May 2019	Dutch	Yes	Yes	No	No
Netherlands	https://www.sense.info/ontdek-je-lichaam/meisjeslichaam/menstruatie/maandverband-en-tampons	25 May 2019	Dutch	Yes	Yes	No	No
New Zealand	https://www.healthnavigator.org.nz/health-a-z/m/menstruation/	25 May 2019	English	Yes	Yes	No	No
New Zealand	https://www.kiwifamilies.co.nz/articles/periods-information-sheet/	25 May 2019	English	Yes	Yes	No	No

Paraguay	http://www.paho.org/par/index.php?option=com_docman&view=download&category_slug=publicaciones-con-contrapartes&alias=384-manual-clinico-de-la-adolescencia&Itemid=253	21 May 2018	Spanish	Yes	No	No	No
Peru	http://www.familycareintl.org/UserFiles/File/Informate%20Flip%20Chart%20optmized.pdf	26 May 2018	Spanish	Yes	No	No	No
Portugal	https://www.dge.mec.pt/sites/default/files/Esaude/guia_adoles_ser.pdf	23 May 2018	Portuguese	No	No	No	No
Portugal	https://juventude.gov.pt/saudesexualidadejuvenil/sexualidade/nossocorpo/paginas/menstrua%C3%A7%C3%A3oeciclomenstrual.aspx	23 May 2018	Portuguese	No	No	No	No
Save the children	https://www.savethechildren.org/content/dam/global/reports/health-and-nutrition/mens-hyg-mgmt-guide.pdf	25 May 2019	English	Yes	Yes	Yes	Yes
Singapore	https://sg.theasianparent.com/your-daughters-first-period/	25 May 2019	English	Yes	Yes	No	No
Singapore	https://www.healthhub.sg/live-healthy/957/help-your-child-cope-with-puberty-and-self-esteem	25 May 2019	English	Yes	No	No	No
South Africa	https://www.health24.com/Lifestyle/Woman/Menstruation/Your-first-period-20120721	25 May 2019	English	Yes	Yes	No	No
Spain	https://www.sanitas.es/sanitas/seguros/es/particulares/biblioteca-de-salud/ginecologia/aparato-genital-femenino/sin012102wr.html	20 May 2018	Spanish	No	No	No	No
Uganda	https://www.education.go.ug/files/downloads/Menstruation_Management_Reader_2017.pdf	25 May 2019	English	Yes	No	No	Yes
UK	http://www.nhs.uk/Conditions/Periods/Pages/Introduction.aspx#Sanitary products	25 May 2019	English	Yes	Yes	Yes	No
Uruguay	https://www.gub.uy/ministerio-salud-publica/sites/ministerio-salud-publica/files/documentos/publicaciones/EligiendoNOS%20para%20adolescentes%20INMUJERES%202017.pdf	21 May 2018	Spanish	No	No	No	No
Uruguay	https://www.unicef.org/uruguay/spanish/Es_parte_de_la_vida_tagged.pdf	26 May 2018	Spanish	No	No	No	No
USA	https://www.acog.org/Patients/FAQs/Your-First-Period-Especially-for-Teens	25 May 2019	English Spanish	Yes	Yes	Yes	No
USA	https://www.everydayhealth.com/pms/a-teens-guide-to-her-first-period.aspx	25 May 2018	English	Yes	Yes	No	No
USA	https://www.girlshealth.gov/body/period/pads.html	25 May 2019	English (+ many)	Yes	Yes	Yes	Yes
USA	http://kidshealth.org/en/teens/menstruation.html	25 May 2019	English Spanish	Yes	Yes	Yes	No
USA	https://kidshealth.org/en/kids/menstruation.html	25 May 2019	English	Yes	Yes	No	No
USA	https://www.medicinenet.com/menstruation/article.htm#how_often_should_i_change_my_padtampon	25 May 2019	English	Yes	Yes	No	No
USA	https://medlineplus.gov/menstruation.html	25 May 2019	English	Yes	Yes	No	No
USA	https://www.plannedparenthood.org/learn/health-and-wellness/menstruation/how-do-i-use-tampons-pads-and-menstrual-cups	25 May 2019	English Spanish	Yes	Yes	Yes	Yes
USA	https://teens.webmd.com/girls/all-about-menstruation#4	25 May 2019	English	No	No	No	No
USA	https://www.webmd.com/parenting/features/daughters-first-period-how-to-prepare#2	25 May 2019	English	Yes	Yes	No	No
USA	https://www.womenshealth.gov/menstrual-cycle/your-menstrual-cycle	25 May 2019	English	Yes	Yes	Yes	Yes
USA	http://youngwomenshealth.org/2013/03/28/period-products/	25 May 2019	English	Yes	Yes	Yes	Yes
Zambia	www.washplus.org/sites/default/files/mhm_toolkit2015.pdf	25 May 2019	English	Yes	Yes	No	Yes
Zimbabwe	http://www.wrc.org.za/mdocs-posts/growing-girls-mhm-booklet-english/growing-girls-mhm-booklet-english-2/	25 May 2019	English	Yes	No	No	Yes

Search term: [Country Name] menstruation information adolescents; Spanish: información para adolescentes menstru* [nombre del pais]; Netherlands: Menstruatie informatie meisjes; German: Menstruation Information madchen; French: Premiere Menstruation information fille; Portuguese: informação para adolescents menstru*

† This pamphlet has information on the sponge for menstruation.

Disclaimer: This was a search to explore the mention of menstrual cup as an option to deal with menstruation, and not intended to be fully inclusive of all potential websites. We do not endorse any website.

Figure S11. Menstrual items mentioned in education material on menarche, from 69 websites, 27 countries

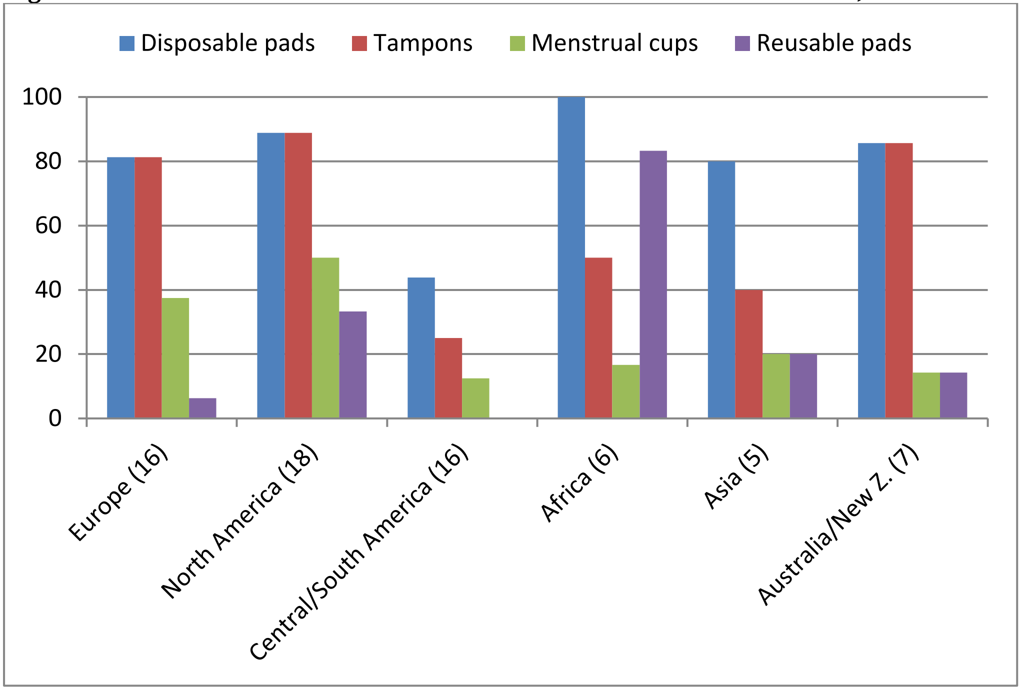

New Z.: New Zealand

For the methods see section 2; for a table of sources, see Table S15. The number of entries per region is in brackets. By region, disposable pads were mentioned in over 80% except for South America (43.8%); tampons were only in Europe, North America and Australia/New Zealand over 80%, whereas MCs were mentioned between 12.5% and 50.0%.

Costs and availability

Using the Menstrual Cup Master List[11] and other sources, 199 brands were identified (in 35 countries, for 20 MCs country of origin unknown); purchasing prices and availability were retrieved for 145 brands (from 32 countries, for 4 MCs the country of origin unknown, Fig S12). These brands were available across 99 countries for purchase; availability of MCs was confirmed by web links or reports on shop sources (Fig S13). The purchasing price of one MC ranged from US$ 0·72 (Chinese MC) to 46·72 (Danish MC); the median and average were 23·35 and 22·90 (prices by global region and country income level: Fig S14 & S15). Most MCs were made from silicone (98%), 1·4% from thermoplastic elastomer, and 0·6% from latex. Availability of MCs by web links or reports on shop sources was confirmed for 99 countries (Fig S18).

Figure S12. Countries with brands of menstrual cups (source) and price available

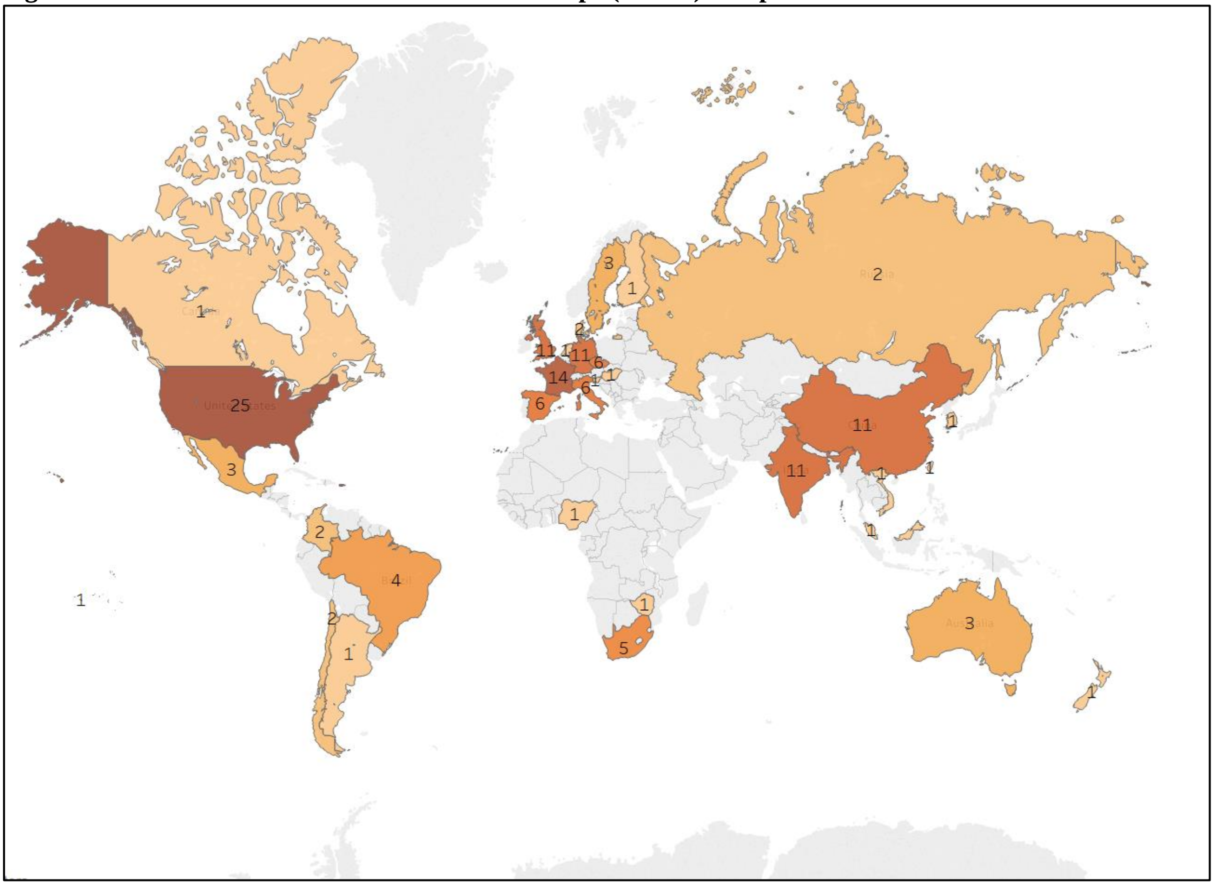

Source: Menstrual Cup Master List, data from 17 September 2018, supplemented with information obtained from experts in the field and web searches. The number in the country indicates the number of brands reporting to be based in that country. Only vaginal cups were included in this figure. We identified 145 menstrual cups with a price available: 141 originated from 32 countries (this map). For 4 menstrual cups the country of origin could not be retrieved.

Figure S13. Menstrual cup availability in 2018

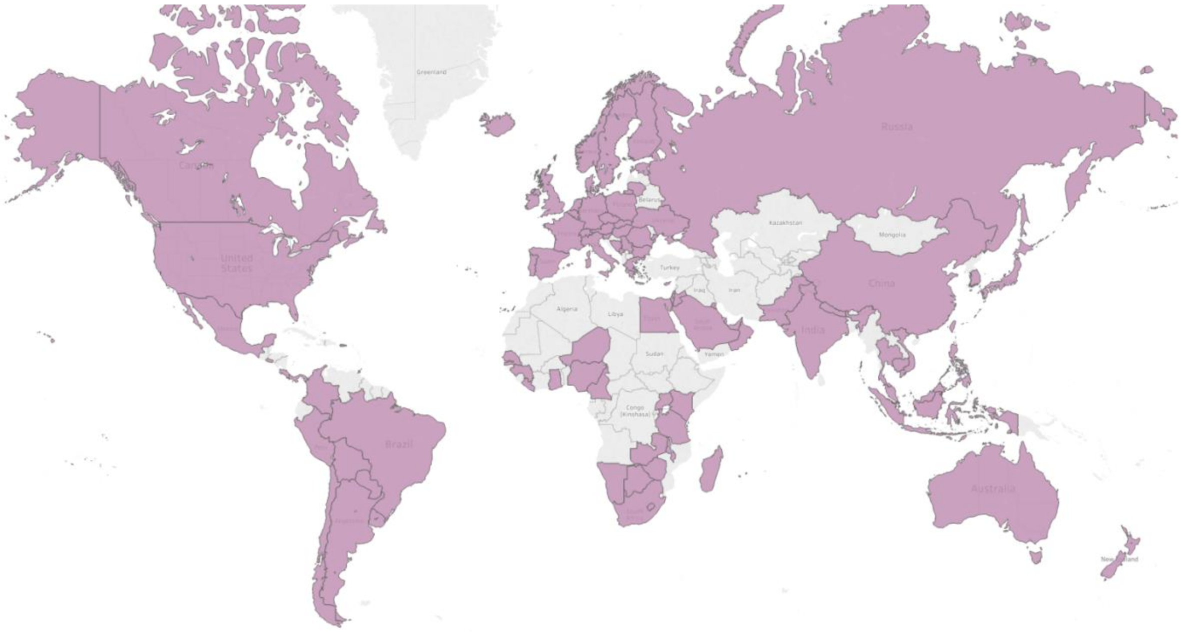

Note: In pink the 99 countries for which we could verify that one or more brands of menstrual cups could be obtained either through the web or in a shop. Only vaginal cups were included in the map. For methods see appendix page 4. Disclaimer: Our internet search may not reflect the local situation in each country.

Figure S14. Retail price of menstrual cup, by brand

S14A. Retail price of menstrual cups based in Europe, by brand (n=65)

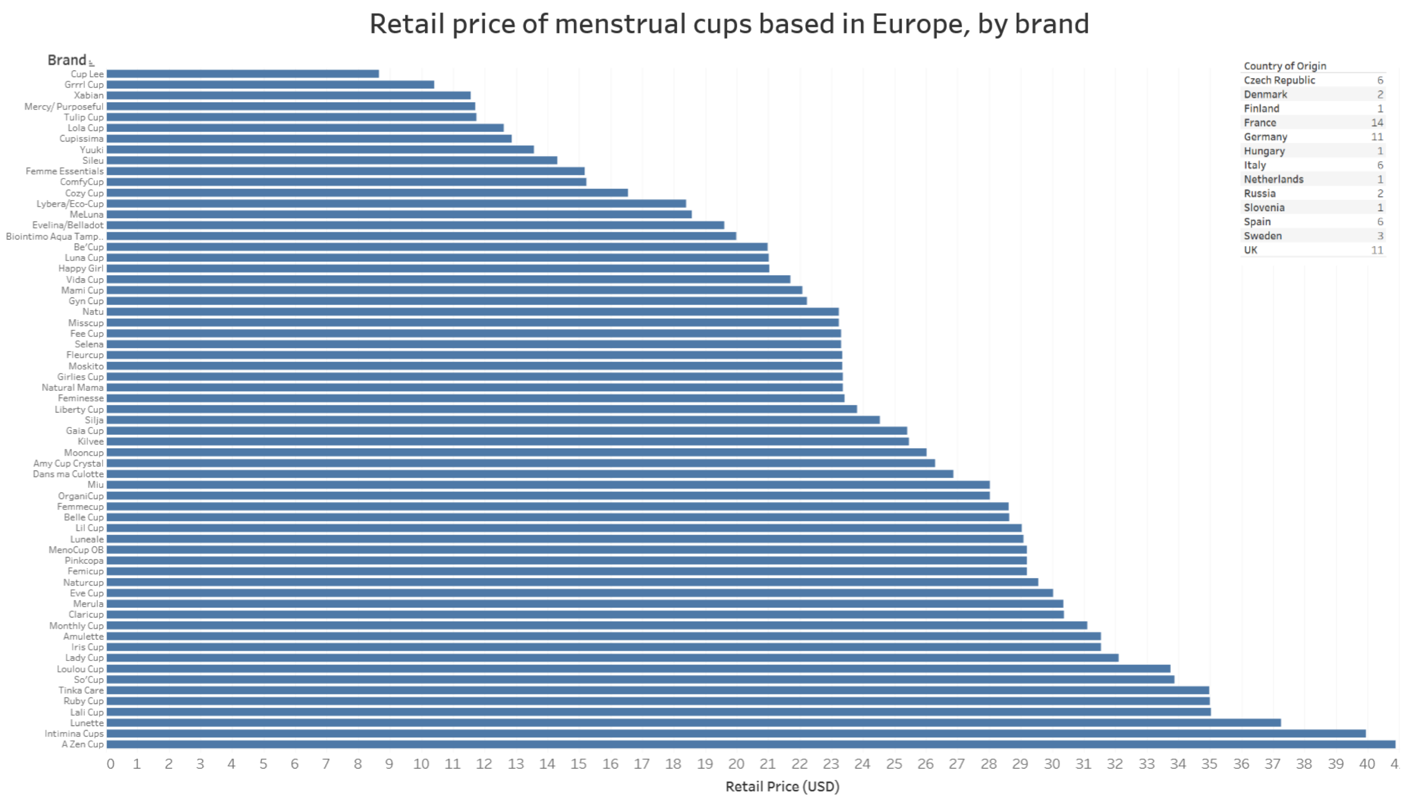

S14B. Retail price of menstrual cups based in Asia, by brand (n=26)

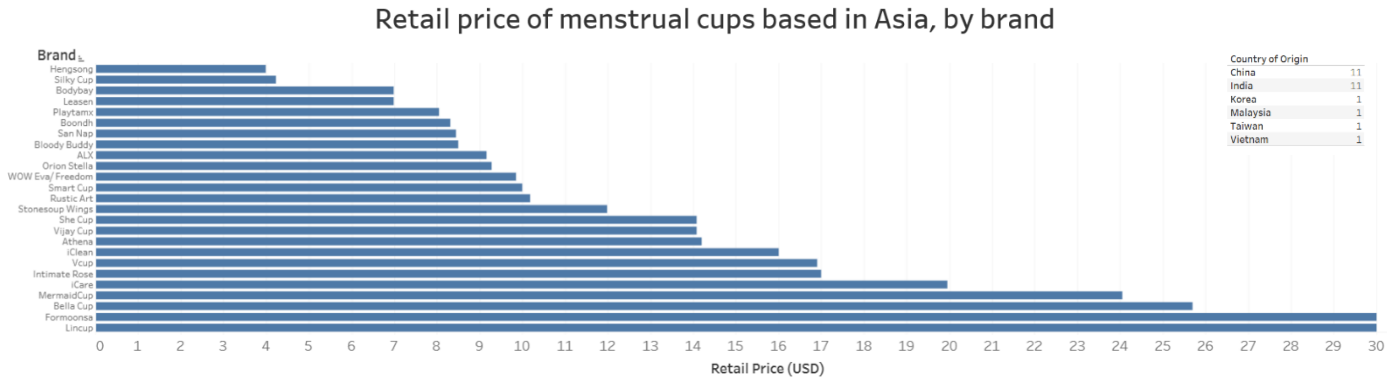

S14C. Retail price of menstrual cups based in North America, by brand (n=29)

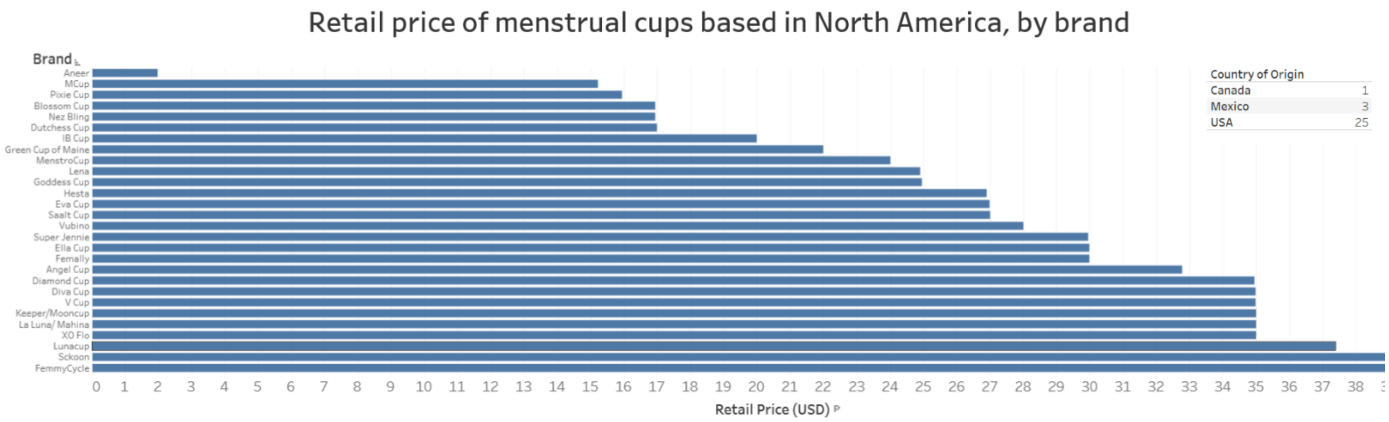

S14D. Retail price of menstrual cups based in South and Central America, by brand (n=9)

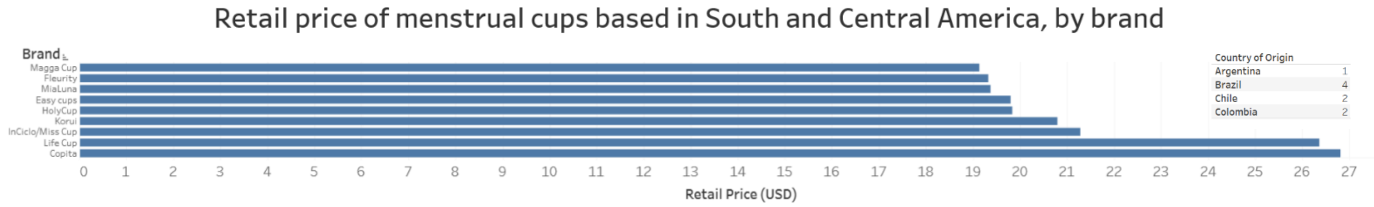

S14E. Retail price of menstrual cups based in Africa, by brand (n=7)

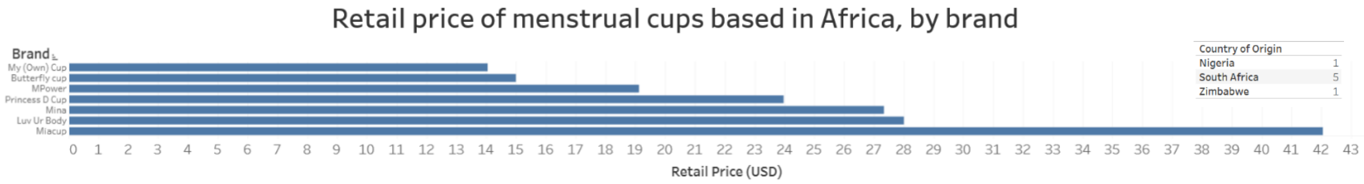

S14F. Retail price of menstrual cups based in Oceania, by brand (n=5)

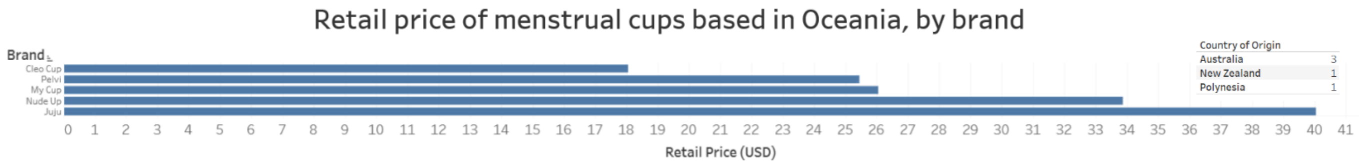

Figure S15. Retail price of menstrual cup, by Gross National Income per capita of the country

S15A. High income countries (n=100)

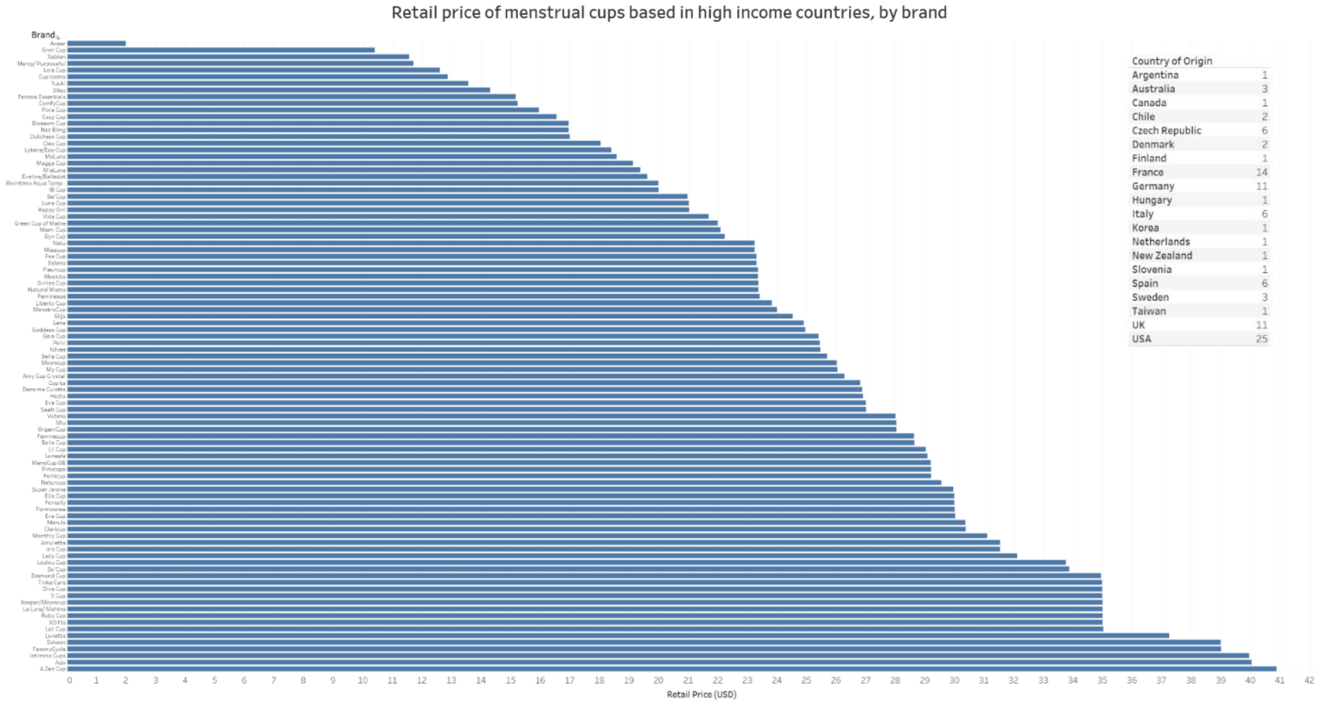

S15B. Upper-middle income countries (n=28)

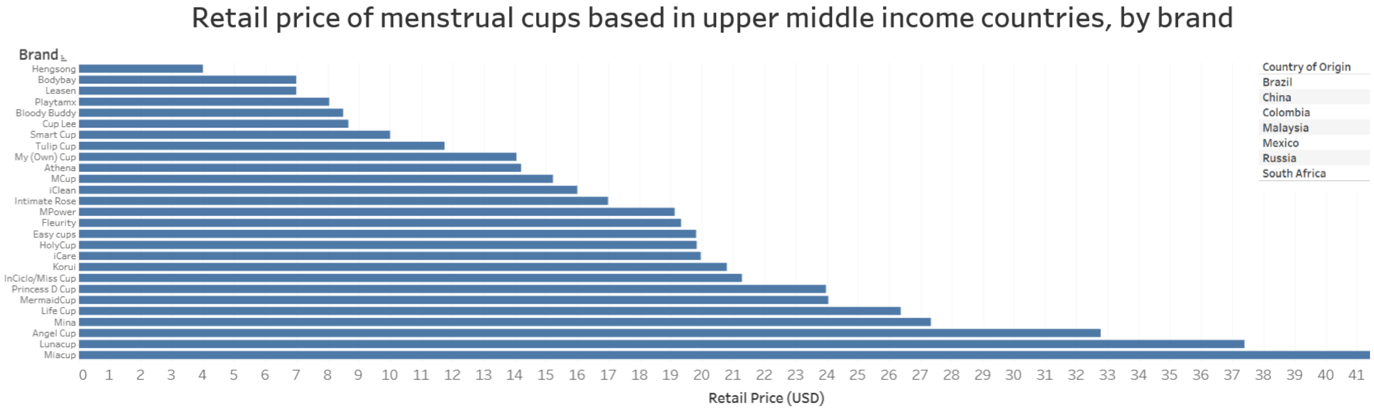

S15C. Lower-middle income countries (n=14)

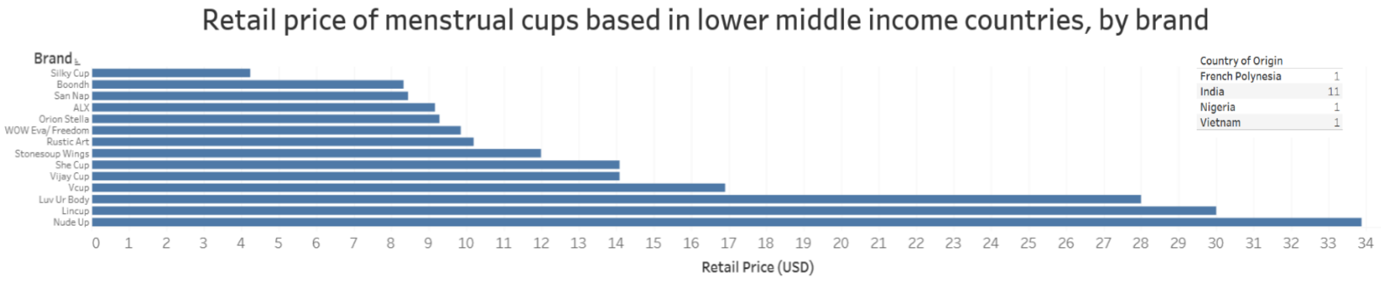

S15D. Low income countries (n=1)

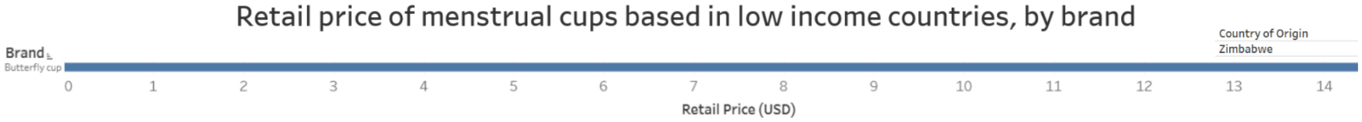

The classification of countries is according to the Atlas method developed by the World Bank which sorts countries by Gross National Income per capita (https://data.worldbank.org/indicator/NY.GNP.PCAP.CD). The rankings applied are from 2017. Taiwan was aggregated with high-income countries, and French Polynesia with lower-middle-income countries. The distribution of brands across country wealth quintiles illustrates that menstrual cups are a luxury item most accessible to higher income populations.

Table S16. Listing of websites used to assess costs and availability of menstrual cups

General			Website
Menstrual Cup Master List			https://menstrualcups.wordpress.com/menstrual-cup-master-list/
**Brand**	**Price in $US**	**Available for purchase**	**Website**
A Zen Cup	40.88	Yes	http://www.azencup.com/shop
ALX	9.17	Yes	http://alexanderlifecare.com/products-detail.php?product_id=1
Amulette	31.54	Yes	https://www.amulette.lt/
Amy Cup Crystal	26.28	Yes	http://www.amycup.com
Aneer	1.98	Yes	https://www.aliexpress.com/item/Feminine-Hygiene-Menstrual-Cup-Diva-Cup-Silicone-Coppetta-Mestruale-Coupe-Menstruelle/32494171702.html?spm=2114.search0104.3.2.6bbc479f9jfbo6&ws_ab_test=searchweb0_0,searchweb201602_1_10152_10151_10065_10344_10068_10130_10324_10342_10547_10325_10343_10546_5722611_10340_10548_10341_10545_10696_5722911_5722811_5722711_10084_10083_10618_10307_10059_306_100031_10103_10624_10623_10622_10621_10620_5722511,searchweb201603_1,ppcSwitch_3&algo_expid=467e602a-53fb-48e1-8362-95de4fc56368-0&algo_pvid=467e602a-53fb-48e1-8362-95de4fc56368&priceBeautifyAB=0
Angel Cup	32.79	Yes	http://www.angelcupmexico.com
Athena	14.20	Yes	https://www.amazon.com/Athena-Menstrual-Cup-Recommended-Guaranteed/dp/B010EB67GK?th=1
Bassrose	0.72	Yes	https://es.aliexpress.com/store/group/Bassrose-Menstrual-cup/316165_504458172.html
Be'Cup	20.97	Yes	http://becup.fr/home/les-produits-becup/
Bella Cup	25.69	Yes	http://www.bellacup.co.kr
Belle Cup	28.63	Yes	http://www.bellecup.co.uk
Biointimo Aqua Tampon	19.98	Yes	https://www.bijobolt.hu/webaruhaz/bijo-termekek?mitkeres=biointimo
Bloody Buddy	8.50	Yes	https://www.amazon.com/Bloody-Buddy-Menstrual-Cup-Menstruation/dp/B01MSAWOZX?th=1
Blossom Cup	16.95	Yes	https://www.blossomcup.com/
Bodybay	6.99	Yes	https://www.amazon.com/Bodybay-Menstrual-Registered-Alternative-Protection/dp/B01LET8F2U
Boondh	8.32	Yes	http://www.boondh.co/home.html
Butterfly cup	15.00	Yes	http://www.thebutterflycup.co.zw
Casco Cup	29.97	Yes	https://cascocup.com/buy/
Claricup	30.37	Yes	http://www.claripharm.com/fr/products
Cleo Cup	18.04	Yes	https://cleo-cup.myshopify.com/
ComfyCup	15.23	Yes	https://www.amazon.co.uk/Comfycup-Menstrual-Cup-Small-Size/dp/B00CQQJGDS/ref=sr_1_3?ie=UTF8&qid=1421289897&sr=8-3&keywords=comfycup
Copita	26.81	Yes	http://www.copitamenstrual.cl
Cozy Cup	16.54	Yes	https://www.amazon.co.uk/Menstrual-CozyCup-CLASSIC-Medical-Silicone/dp/6040125934/ref=sr_1_1_s_it?s=drugstore&ie=UTF8&qid=1507900762&sr=1-1&keywords=CozyCup
Cup Lee	8.66	Yes	http://cuplee.ru
Cupissima	12.87	Yes	http://www.cupissima.com/index_fr.html
Dans ma Culotte	26.87	Yes	http://dansmaculotte.com/en/
Diamond Cup	34.95	Yes	http://www.mydiamondcup.com/store/p4/p.html
Diva Cup	34.99	Yes	http://www.divacup.com
Dutchess Cup	16.99	Yes	https://pelvichealthshop.com/collections/all
Easy cups	19.80	Yes	http://www.easycups.ca/easycup/shop/
Ella Cup	29.99	Yes	https://www.ellacup.com/
Eva Cup	26.99	Yes	https://www.anigan.com/products/anigan-evacup-single-pack
Eve Cup	32.71	Yes	http://www.nalayachakana.com/eve-cup/
Eve Cup	27.33	Yes	http://www.evecups.uk/why-the-eve-cup/
Evelina/Belladot	19.61	Yes	http://www.belladot.com/products/evelina-menstrual-cup
Fee Cup	23.30	Yes	https://feecup.de/onlineshop/menstruationstasse-classic/
Female Cup	46.72	Yes	http://femalecup.com/products/
Femally	29.99	Yes	https://www.femallay.com/collections/healthy-menstrual-care/products/femallay-easy-empty-menstrual-cup
Femicup	29.20	Yes	http://www.femicup.com/content/18-comprar-femicup

Feminesse	23.42	Yes	https://feminesse.co.uk/product/feminesse-menstrual-cup/
Femme Essentials	15.17	Yes	https://www.amazon.de/Femme-Essentials-Menstruationstasse-Menstruationskappe-medizinischem/dp/B012BNU3CS
Femmecup	28.62	Yes	http://www.femmecup.com
FemmyCycle	39.00	Yes	https://femmycycle.com/
Fleurcup	23.35	Yes	http://fleurcup.com
Fleurity	19.32	Yes	http://fleurity.com.br
Formoonsa	30.00	Yes	https://www.formoonsacup.com/english
Gaia Cup	25.40	Yes	http://www.gaiacup.com
Girlies Cup	23.36	Yes	http://www.girlies-cup.com/
Goddess Cup	24.95	Yes	https://www.goddesshealthco.com/collections/the-goddess-cups
Green Cup of Maine	21.99	Yes	http://www.greencupofmaine.com/
GRRRL Cup	10.40	Yes	https://www.amazon.co.uk/dp/B0728NVJ7H?psc=1
Gyn Cup	22.22	Yes	http://www.mujkalisek.cz/
Happy Girl	21.03	Yes	https://www.remede-naturel.net/en/our-natural-products/158-happy-girl-cup-coupe-menstruelle-.html
Hengsong	3.99	Yes	https://www.amazon.com/Hengsong-Reusable-Feminine-Protection-Menstrual/dp/B00PDFYGCA/ref=pd_sim_121_3?ie=UTF8&dpID=31I5dgRCTbL&dpSrc=sims&preST=_AC_UL160_SR160%2C160_&psc=1&refRID=2WDW586WZQF7PDZG1SG8
Hesta	26.90	Yes	https://www.hestaorganic.com/product-category/menstrual-cup/
HolyCup	19.83	Yes	http://holycup.com.br/
IB Cup	19.99	Yes	http://improvingbirth.org/product/ib-menstrual-cup/
iCare	19.95	Yes	https://www.icare-menstrual-cup.com/#order-now
iClean	16.00	Yes	https://www.ebay.com/p/iClean-Medical-Grade-Silicone-Reusable-Menstrual-Period-Cup-2x-large/935300630
InCiclo/Miss Cup	21.28	Yes	www.inciclo.com.br
Intimate Rose	16.99	Yes	https://www.intimaterose.com/products/rose-cup-menstrual-cup-by-intimate-rose-fda-registered-reusable-body-safe-medical-silicone-alternative-to-tampons-pads-heavy-light-flow-period
Intimina Cups	39.95	Yes	http://www.intimina.com/en/lily_cup_pd.php
Iris Cup	31.54	Yes	www.iriscup.com
Juju	40.04	Yes	http://www.juju.com.au
Keeper/Mooncup	35.00	Yes	http://www.keeper.com
Kilvee	25.46	Yes	https://www.klivee.com/
Korui	20.80	Yes	http://www.korui.com.br/
La Luna/ Mahina	35.00	Yes	http://www.mysacredcycle.com/home/
Lady Cup	32.11	Yes	http://www.ladycup.eu
Lali Cup	35.03	Yes	http://lalicup.si
Leasen	6.99	Yes	https://www.amazon.com/LEASEN-Health-Silicone-Menstrual-Period/dp/B016RHOWUI
Lena	24.90	Yes	http://www.lenacup.com
Liberty Cup	23.82	Yes	https://www.cocooncenter.co.uk/liberty-cup-menstrual-cup-size-2/22793.html
Life Cup	26.37	Yes	http://lifecup.co/en/comprar/
Lil Cup	29.02	Yes	http://www.menstruacni-kalisek.cz/15-menstruacni-kalisky-lilcup-od-199-kc
Lincup	30.00	Yes	http://us.lintimate.net/lincup
Lola Cup	12.60	Yes	http://lolaperiodcup.com/#chisiamo
Loulou Cup	33.76	Yes	https://www.en.louloucup.com/
Luna Cup	21.01	Yes	http://www.menstruacnykalisokluna.sk/eshop/
Lunacup	37.38	Yes	http://lunacup.mx
Luneale	29.08	Yes	https://www.lacupluneale.com/
Lunette	37.25	Yes	http://www.lunette.com
Luv Ur Body	28.00	Yes	http://luvur-body.com
Lybera/Eco-Cup	18.40	Yes	http://www.lybera.it
Magga Cup	19.13	Yes	http://maggacup.com.ar
Mami Cup	22.08	Yes	http://www.coppetta-mestruale.com

MCup	15.21	Yes	https://mcup.com.mx/web/tienda/
MeLuna	18.57	Yes	https://www.me-luna.eu/en_GB
MenoCup OB	29.19	Yes	http://www.menocup-ob.de/english/
MenstroCup	24.00	Yes	http://www.menstrocup.com/product/menstrocup-from-femogene/
Mercy/ Purposeful	11.70	Yes	https://www.amazon.co.uk/Mercy-Menstrual-Cup-environment-childbirth/dp/B00YEY8OLK
MermaidCup	24.05	Yes	http://cottonmermaid.com/mermaidcup-menstrual-cup/
Merula	30.36	Yes	http://www.merula-cup.de
Miacup	42.05	Yes	www.rethinkthetampon.co.za
MiaLuna	19.37	Yes	http://mialuna.cl/
Mina	27.34	Yes	https://minacup.org/
Misscup	23.24	Yes	https://www.misscup.fr/produit/misscup-rose/
Miu	28.03	Yes	https://miu-cup.com
Monthly Cup	31.11	Yes	https://www.menstrualcup.com/gb
Mooncup	26.02	Yes	http://www.mooncup.co.uk
Moskito	23.35	Yes	http://www.lumunu.de/search/moskito?row=0
MPower	19.11	Yes	http://mpowercup.co.za/
My (Own) Cup	14.04	Yes	http://www.myowncup.co.za
My Cup	26.04	Yes	http://www.mycup.co.nz/menstrual-cups/
Natu	23.23	Yes	http://www.natulovesyou.com/
Natural Mama	23.36	Yes	http://www.pannolinilavabili.org/
Naturcup	29.55	Yes	http://www.naturcup.com
Nez Bling	16.95	Yes	https://nezbling.com/products/menstrual-cup
Nude Up	33.87	Yes	http://nudeup.co/en/product/nudeup-lagoon-blue/
OrganiCup	28.03	Yes	http://www.organicup.eu
Orion Stella	9.28	Yes	https://www.snapdeal.com/brand/orion-stella/feminine-hygiene-menstrual-cups
Pelvi	25.44	Yes	https://pelvi.com/collections/all/products/menstrual-cup
Pinkcopa	29.20	Yes	http://www.pinkcopa.com
Pixie Cup	15.95	Yes	https://www.pixiecup.com/collections/all
Playtamx	8.04	Yes	https://www.tmall.com/mlist/regular_U3rJhD30cV5lmhkcs4w6UACbjK5xmHBvUiaALdBkA2o.html
Princess D Cup	23.98	Yes	http://www.princessdmenstrualcup.com/PrincessD%20Flyer_Caster%20Semenya_2017.pdf
Ruby Cup	35.00	Yes	http://www.ruby-cup.com
Rustic Art	10.19	Yes	http://www.rusticart.in/choose-a-cup/
Saalt Cup	27.00	Yes	https://www.saaltco.com/shop/saalt-cup-small/
San Nap	8.45	Yes	http://www.nykaa.com/sannap-menstrual-cup.html
Sckoon	38.99	Yes	http://sckooncup.com
Selena	23.30	Yes	http://www.selenacup.at/en/
She Cup	14.09	Yes	http://www.shecup.com/
Sileu	14.30	Yes	http://sileu.com/en/cup/sileu-violet-menstrual-cup-made-from-medical-grade-silicone-reusable-model-soft/
Silja	24.53	Yes	http://www.silja-cup.com/epages/79109918.sf/de_DE/?ObjectPath=%2FShops%2F79109918%2FCategories%2FSilja_Shop
Silky Cup	4.23	Yes	http://www.silkycup.com
Smart Cup	9.99	Yes	https://www.amazon.com/Smart-Cup-Menstrual-Period-Childbirth/dp/B00WCDUL8E?th=1
So'Cup	33.87	Yes	https://socup.fr/boutique-2
Stonesoup Wings	11.99	Yes	https://stonesoup.in/pages/stonesoup-wings
Super Jennie	29.95	Yes	http://www.superjennie.com
Tinka Care	34.98	Yes	http://www.tinkacare.de/
Tulip Cup	11.74	Yes	http://vagiton.ru/kapa
V Cup	34.99	Yes	https://v-cups.com/collections/all
Vcup	16.91	Yes	http://vcup.co.in/index.php

Vida Cup	21.69	Yes	http://www.medihealth1.com/
Vijay Cup	14.09	Yes	https://www.amazon.in/Menstrual-Vijay-Cup-Foldable-Reusable/dp/B01H90EV08
Vubino	28.00	Yes	http://vubino.com/
WOW Eva/ Freedom	9.86	Yes	http://www.nykaa.com/wow-eva-reusable-menstrual-cup-wash-size-m-under-30-year.html?ptype=product&id=35022
Xabian	11.56	Yes	https://www.amazon.de/Menstruationstasse-Xabian-medizinisches-Silikon-Gr%C3%B6%C3%9Fenauswahl/dp/B0177P2Q48
XO Flo	35.00	Yes	https://gladrags.com/product/451/XO-Flo.html
Yuuki	13.56	Yes	http://www.yuuki.cz
Charlene	19.00	Yes	https://www.amazon.com/dp/B00TACX6LY?psc=1
Diggold	19.77	Yes	https://www.amazon.com/Menstrual-Cup-FDA-Registered-Alternative/dp/B0718X4DML?th=1
Invisicup	17.47	Yes	https://www.amazon.com/InvisiCup-Perfect-Menstrual-Starter-Silicone/dp/B01MSLCV0H?th=1
Shelcup	11.99	Yes	https://www.amazon.com/Shelcup-Mesrual-Healthy-Comfortable-Period/dp/B01N4NQ5G1?th=1
Afreecup	--	No	No active weblink
Alicia/Hygina	--	No	No active weblink
Anytime	--	No	https://www.aliexpress.com/item/2015-Anytime-Brand-Wholesale-Reusable-Medical-Grade-Silicone-Menstrual-Cup-Feminine-Hygiene-Product-Lady-Menstruation-Copo/32288941057.html?s=p
Aphrodite	--	No	https://es.aliexpress.com/item/2pcs-lot-Menstruation-Cup-Reusable-Softcup-Coletor-Menstrual-Diva-Cup-Medical-Silicone-Menstrual-Cup/32489932239.html
Ava Loona	--	No	No active weblink
Beianyi	--	No	https://es.aliexpress.com/item/Free-Shipping-Silicone-Copa-Menstrual-Menstruation-Cup-Coppetta-Mestruale-Softcup-Menstrual-Cup-Lady-Cup-Menstrual-Care/32488786464.html
Carrefour	--	No	No active weblink
Cerene	--	No	No active weblink
Continuon	--	No	No active weblink
Crystal Cup	--	No	No active weblink
Cup'ax	--	No	https://www.cupax.co/en/
Cuplette	--	No	No active weblink
Daisy Cup	--	No	No active weblink
Easy Cup	--	No	No active weblink
Easy Life	--	No	http://www.skynector.com/en/products-look.aspx?id=116
EcoClean	--	No	https://www.amazon.com/Menstrual-Organic-Antibacterial-Reusable-Eco-friendly/dp/B01J4G19YG
Farma Cup	--	No	http://www.farmacare.it/en/catalog/disposables/farmacup-menstrual
Femcup	--	No	https://www.facebook.com/femcup.gr/
Femma's Cup	--	No	No active weblink
Freeysia/Smartcup2 8	--	No	https://sites.google.com/site/wwwsmartcup28/
Gea Cup	--	No	No active weblink
Genti-She	--	No	http://genti-she.weebly.com
Green Donna	--	No	www.greendonna.com
Honeymoon	--	No	https://es.aliexpress.com/item/5pcs-lot-Menstrual-Care-Coupe-Menstruelle-Menstrual-Menstruation-Cup-Medical-Grade-SiliconeTo-Discharge-Menstrual-Cup-Diva/32510865333.html
Inticup	--	No	http://intilife.com/Buy
Ivita	--	No	https://www.aliexpress.com/item/IVITA-Feminine-Hygiene-Silicone-Products-Vagina-Use-Lady-Menstrual-Cups-Alternative-Tampons-5pcs-lot-Transparent-Color/32722288012.html
Jasmine Cup	--	No	No active weblink
La Maleta Roja	--	No	http://lamaletaroja-palma.com/
Lila Cup	--	No	http://www.lilacup.ru
Lis Cup	--	No	https://www.liscup.com/
Loon	--	No	https://www.kickstarter.com/projects/700989404/looncup-the-worlds-first-smart-menstrual-cup
Luno	--	No	http://www.kaliskyluno.cz/
LuxuryBody	--	No	No active weblink
Melody Susie	--	No	https://www.amazon.com/MelodysusieTM-Quality-Crystal-Menstrual-Childbirth/dp/B00EPE4NG4
MiCup	--	No	No active weblink
Monicup	--	No	https://www.amazon.de/dp/B01EMN7PT4?psc=1

Monzcare/Rainbow Cup	--	No	No active weblink
My Cup/ Tati	--	No	http://www.mycup.co.nz/product/tati-menstrual-cup-02/
MyBelle	--	No	No active weblink
Nabelle	--	No	https://www.aliexpress.com/item/2015-NABELLE-Brand-New-Feminine-Hygiene-Women-Silicone-Menstrual-Cup-Diva-Cup-Copa-Menstrual-Coupe-Menstruelle/32489355919.html
Pretty Women	--	No	https://www.amazon.com/OVERSTOCK-SALE-Pretty-Woman-Menstrual/dp/B00NLD5W2Y
Puramour/ Dream Lover	--	No	No active weblink
Si-bell	--	No	https://sibellmenstrualcup.com/
Skinco	--	No	https://www.amazon.com/Menstrual-Alternative-Sanitary-Feminine-Childbirth/dp/B01EKW6WJQ/ref=as_li_ss_tl?ie=UTF8&qid=1522175682&sr=8-3&keywords=skinco+menstrual+cup&linkCode=sl1&tag=menstrualcup0d-20&linkId=119b5b5d47c597486452b739fdd8c661&linkCode=w61&imprToken=raHxBsZZuVOIuac4AyVE4A&slotNum=0
Smilucky	--	No	https://www.amazon.co.uk/d/72h/Smilucky-Piece-Reusable-Medical-Silicone-Menstrual-Small/B01HT5BEKI/
Sunsmiler	--	No	No active weblink
Sure Cycle	--	No	No active weblink
The Flower Pot	--	No	No active weblink
Uni Cup	--	No	https://www.amazon.com/MENSTRUAL-hygienic-Set-Yoga-swimming-menstrual/dp/B01JBKS93Y
Victoria Love	--	No	https://www.facebook.com/victoriaslove/
Well Done	--	No	No active weblink
Zengina	--	No	No active weblink
Aiwo	--	No	https://www.facebook.com/NOOD-668883369920060

Table S17. Price estimates for commonly used menstrual pads and tampons to obtain an average price per unit

	Country	Price local currency	Exchange rate (31 August 2018)	USD Price	# per pack	Number of packs	Units	Price per unit	Average price
**TAMPAX**									0.246204
	US	17.04	1	17.04	54	2	108	0.157778	
	UK	17.99	1.30158	23.41542	48	2	96	0.243911	
	India	539	0.0141	7.5999	20	1	20	0.379995	
	Spain	5.18	1.16806	6.050551	30	1	30	0.201685	
	China	185.81	0.14623	27.171	20	4	80	0.339637	
	Canada	7.99	0.77206	6.168759	40	1	40	0.154219	
**ALWAYS**									0.288575
	US	41.82	1	41.82	30	6	180	0.232333	
	UK	4.39	1.30158	5.713936	10	2	20	0.285697	
	India	215	0.0141	3.0315	9	1	9	0.336833	
	Spain	14.29	1.16806	16.69158	12	4	48	0.347741	
	China	183.25	0.14623	26.79665	30	3	90	0.297741	
	Canada	8.98	0.77206	6.933099	30	1	30	0.231103	
**O.B.**									0.179317
	US	6.23	1	6.23	40	1	40	0.15575	
	UK	9	1.30158	11.71422	32	3	96	0.122023	
	India	378	0.0141	5.3298	20	2	40	0.133245	
	Spain	8.99	1.16806	10.50086	56	1	56	0.187515	
	China	91.14	0.14623	13.3274	40	1	40	0.333185	
	Canada	7.47	0.77206	5.767288	40	1	40	0.144182	
**Kotex (Pads)**									0.331882
	US	27.59	1	27.59	44	3	132	0.209015	
	UK	11.95	1.30158	15.55388	14	4	56	0.277748	
	India	1963	0.0141	27.6783	50	1	50	0.553566	
	Spain	22.06	1.16806	25.7674	12	4	48	0.536821	
	China	299.43	0.14623	43.78565	28	6	168	0.260629	
	Canada	11.93	0.77206	9.210676	60	1	60	0.153511	

Note: The search through Amazon prime was conducted on the 5th of October 2018.
The average price for pads used in the calculations was $US 0.31 for pads (the average of the price for Always and Kotex), and $US 0.21 for tampons (the average of the price for Ob and Tampax).

Table S18. Calculations of costs and waste for sanitary pads and tampons over 10 years

No of pads per month	9	12	15	20	25		No of tampons per month	9	12	15	20	25
**Cost of pad (US$)**	**0.31**	**0.31**	**0.31**	**0.31**	**0.31**		**Cost of tampon (US$)**	**0.21**	**0.21**	**0.21**	**0.21**	**0.21**
No of pads per yr	117	156	195	260	325		No of tampons per yr	117	156	195	260	325
No of pads per 10 yrs	1170	1560	1950	2600	3250		No of tampons per 10 yrs	1170	1560	1950	2600	3250
Costs per yr (US$)	36.27	48.36	60.45	80.6	100.75		Costs per yr (US$)	24.57	32.76	40.95	54.6	68.25
Costs per 10 yrs (US$)	362.7	483.6	604.5	806	1007.5		Costs per 10 yrs (US$)	245.7	327.6	409.5	546	682.5
Blood loss/period (grams)	35	35	35	35	35		Blood loss/period (grams)	35	35	35	35	35
Blood loss/year (grams)	455	455	455	455	455		Blood loss/yr (grams)	455	455	455	455	455
Blood loss/10 yr (grams)	4550	4550	4550	4550	4550		Blood loss/10 yr (grams)	4550	4550	4550	4550	4550
Blood loss/10 yr (kg)	4.55	4.55	4.55	4.55	4.55		Blood loss/10 yr (kg)	4.55	4.55	4.55	4.55	4.55
Weight/pad (grams)	6.8	6.8	6.8	6.8	6.8		Weight per tampon (grams)	3.6	3.6	3.6	3.6	3.6
Weight pads per yr (grams)	795.6	1060.8	1326	1768	2210		Weights tampons per yr (grams)	421.2	561.6	702	936	1170
Weight pads per 10 yrs (grams)	7956	10608	13260	17680	22100		Weights tampons per 10 yrs (grams)	4212	5616	7020	9360	11700
Weight pads per 10 yrs (kg)	7.956	10.608	13.26	17.68	22.1		Weight tampons per 10 yrs (kg)	4.212	5.616	7.02	9.36	11.7
Percentage plastic in pad (%)	34.78992	34.78992	34.78992	34.78992	34.78992		Percentage plastic in tampon (%)	4.485249	4.485249	4.485249	4.485249	4.485249
Plastic waste in pad in 10 yrs (kg)	2.767886	3.690514	4.613143	6.150857	7.688571		Plastic waste in tampon in 10 yrs (kg)	0.191379	0.255171	0.318964	0.425286	0.531607
Non-plastic waste in pad in 10 yrs (kg)	5.188114	6.917486	8.646857	11.52914	14.41143		Non-plastic waste in tampon in 10 yrs (kg)	4.075465	5.433954	6.792442	9.056589	11.32074
Non-plastic waste + blood, pads, 10 yrs (kg)	9.738114	11.46749	13.19686	16.07914	18.96143		Non-plastic waste + blood, tampons, 10 yrs (kg)	8.625465	9.983954	11.34244	13.60659	15.87074
Total waste pads 10 yrs (kg)	12.51	15.16	17.81	22.23	26.65		Total waste tampons 10 yrs (kg)	8.82	10.24	11.66	14.03	16.40

yr: year.

Figure S16. Estimation of costs and waste of different products used for menstruation over 10 years

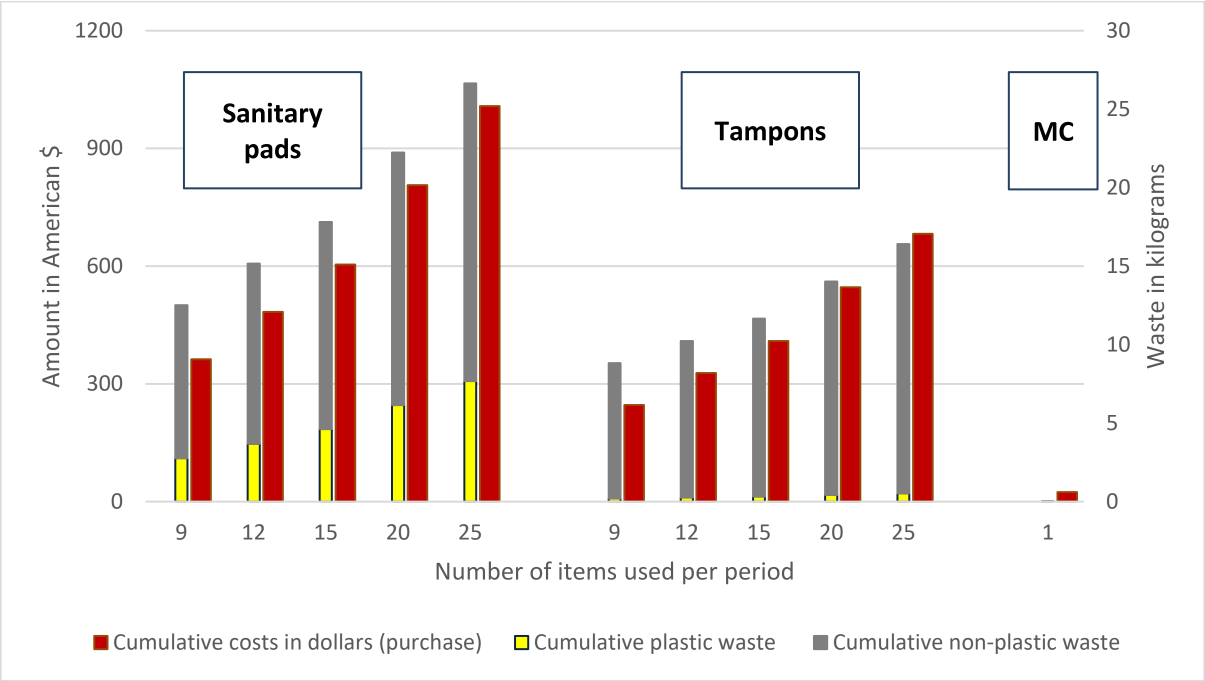

MC: menstrual cup. Note: Cost estimates of sanitary pads and tampons conducted in October 2018.
Using accumulated estimates over 10 years, purchase costs of and waste from consistent MC-use (vaginal cup) would be a small fraction of the waste and purchase costs of pads or tampons: e.g. 5% and 7% of the purchase costs and 0·4% and 6% of the plastic waste when using 12 pads or tampons per period, respectively. Combined use of menstrual products during a period (e.g. panty liners for light days), the use of more than one cup over 10 years, inflation and production costs, and waste estimates were not taken into account in these calculations.

3. Web resources

Blogs, forums and discussion platforms

We searched for supplemental information pertaining to the use, acceptability, and effectiveness of menstrual cups among the non-scientific community. Online blog, forum, and discussion platforms were assessed for relevance and for information not previously discussed in the primary scientific literature. These platforms were accessed using google searches and screened until saturation (3 pages without new references). Searches were conducted for adverse events relating to menstrual cups, including topics on menstrual cup retention, infection, and endometriosis.

Most entries surrounded the benefits of using menstrual cups, examining prices, environmental impact, and the changing narrative around menstruation. The discussion forums and blogs highlighted the nuances of insertion, wearing, and removal, as these processes were the areas where people experienced the most difficulty. Many women share their experiences online, with others offering advice and encouragement.

On a forum for menstrual cup support (menstrual_cups.livejournal.com), one user describes intensified pain while using the DivaCup, only on specific days of her cycle. A responder explained that this pain may be due to suction of the cup on her cervix (due to the cup length). She is instructed to try flipping the cup inside-out to decrease the length and placement of the cup within the vagina. Another responder agrees and explains that the cervix changes in length throughout the cycle, which may affect the needed length of the menstrual cup. The firmness of the DivaCup is listed as a potential area for consideration, and alternative, less-firm menstrual cup brands are recommended.

Another forum entry, using the same platform as above, requests advice for inserting the cup. The initial poster explained she has experienced a lot of pain while attempting to insert the cup. Responders recommend different techniques for insertion, including relaxation, positioning of cup, different cup folds, and the use of lubrication. Additional responses recommend that if pain persists after trying the recommendations, the user should see a doctor for possible vaginal pain conditions, including vulvodynia.

Serious adverse events are also discussed in blogs and forums. In the comment sections of a blog by Dr Jen Gunter (Toxic Shock Syndrome is also Related to Menstrual Cups), many cup users asked about symptoms and experiences. "Airstreamtatoo" commented that she experienced TSS as a result of using the Mooncup, even with regular washing and boiling. She later commented on the resulting discussion that the source of the bacteria was in uncleaned ventilation holes. The user stated that she did continue using a separate menstrual cup after the TSS case. This case has not been reported in a scientific publication.

The discussion on TSS was developed further in a blog titled “Menstrual Cups are More Likely to Cause Toxic Shock Syndrome than Tampons.” This blog cites research from the University Hospital of Lyon which claims that menstrual cups are more likely to cause TSS than tampons due to the fact that they allow more air into the vagina. Additionally, since they are not removed as frequently as tampons, the researchers claim this allows for a greater opportunity for bacteria growth. This may be referring to the paper by Nonfoux et al. [54], which used an in-vitro model to examine the development of *S. aureus* and toxins with cups and tampons. However, it is not clear how applicable this in-vitro model is to the “real-life” situation, presented in other papers showing no difference between groups using a variety of products.

Though many of the broad benefits and problems associated with menstrual cups are discussed in scientific publications, the specific conversations and nuances of cup use on websites can provide critical insight into user experiences and can highlight issues missed by researchers. These platforms are able to reach individuals around the world, enabling a wide diversity of individuals to ask questions or provide responses.

Table S19. Informal information on menstrual cups and serious adverse events on the internet

Key Word Search	URL	Title	Author (If Available)	Date Published	Type of Source	Content
Menstrual cup + infection	http://metro.co.uk/2017/07/06/menstrual-cups-are-more-likely-to-cause-toxic-shock-syndrome-than-tampons-claims-study-6758784/	Menstrual Cups are More Likely to Cause Toxic Shock Syndrome than Tampons, Claims Study	Miranda Larbi	July 6, 2017	Blog, website	Cites research from the University hospital of Lyon which claims that menstrual cups are more likely to cause toxic shock syndrome than tampons due to the fact that they allow more air into the vagina. Additionally, since they are not removed as frequently as tampons, the research claim this allows for more opportunity for bacteria growth. Note authors: this may have referred to the in-vitro study of Nonfoux et al., 2018[54]
Menstrual cup + issues	https://drjengunter.wordpress.com/2015/10/28/toxic-shock-syndrome-is-also-related-to-menstrual-cups/	Toxic Shock Syndrome is also Related to Menstrual Cups	N/A	Variety	Forum, blog, website	In the comment sections of the blog by Dr Jen Gunter (see above), many cup users asked about symptoms and experiences. "Airstreamtatoo" commented on November 1, 2015 stating that she experienced TSS as a result of using the Mooncup, even with regular washing/boiling. She later commented on January 1, 2016 that the source of the bacteria was in uncleaned ventilation holes. The user did continue using a menstrual cup after the toxic shock syndrome (a different cup)
Menstrual cup + insertion	https://menstrual-cups.livejournal.com/	Menstrual Cups - DivaCup, Mooncup, Instead, Lunette, MiaCup	N/A	Variety	Forum, website	Forum for menstrual cup users to ask questions and reply to concerns from current of potential cup users. The forum is open to anyone wishing to participate.
Menstrual Cup + Horror Stories	https://www.thehairpin.com/2014/01/the-best-time-a-diva-cup-suctioned-itself-to-my-cervix/	The Best Time a Diva Cup Suctioned Itself to My Cervix	Amy Starfish	January 10, 2014	Blog, website	Recounts her experience with the diva cup- explaining that after a day of use, she could not remove it. She left it in for 24 hours then went to a local Urgent care center. The nurse had never heard of the diva cup and could not remove it. She was sent to the ER, where a nurse had to remove the cup from the opening of her cervix using large scissor forceps. She claims it moved because she did not properly turn the cup 360 degrees to create a proper seal.
Menstrual cup + issues	https://drjengunter.wordpress.com/2015/10/28/toxic-shock-syndrome-is-also-related-to-menstrual-cups/	Toxic Shock Syndrome is also Related to Menstrual Cups	Jen Gunter	October 28, 2015	Blog, website	The author explains that there are risks of TSS while using the menstrual cup. She explains the case of TSS (Mitchell 2015) has only occurred once and that this should not stop anyone from using menstrual cups.
Menstrual cup + toxic shock	https://www.consumerreports.org/women-s-health/menstrual-cups-linked-to-toxic-shock-syndrome/	Menstrual Cup linked to toxic shock syndrome, new study finds	Hallie Levine	4/20/2018	Article, website	An article discusses a new study (linked at end) which found that menstrual cups were just as likely as tampons to carry TSS-causing bacteria. "Impact of currently marketed tampons and menstrual cups on Staphylococcus aureus growth and TSST-1 production in vitro." http://aem.asm.org/content/early/2018/04/02/AEM.00351-18.abstract Note authors: this referred to the in-vitro study of Nonfoux et al., 2018[54]
Menstrual cup + endometriosis	http://www.prweb.com/releases/2005/07/prweb258853.htm	Menstrual Cups Fail to Warn of Endometriosis Risk	PRWEB	July 12, 2005	Article, website	An article stating that menstrual cup manufacturers have failed to put warnings on labels regarding risk of endometriosis, as FDA has stated it is "plausible"

References

1. Phillips-Howard PA, Nyothach E, Ter Kuile FO, et al. Menstrual cups and sanitary pads to reduce school attrition, and sexually transmitted and reproductive tract infections: a cluster randomised controlled feasibility study in rural Western Kenya. *BMJ Open* 2016; **6**: e013229.
2. Femme International. Monitoring & Evaluation Report Kilimanjaro Region 2017: Successes and lessons learned from the Thaweza program. 2017. https://www.femmeinternational.org/wp-content/uploads/2018/09/Femme-International-ME-Report-2017.pdf (accessed May 28, 2019).
3. Madziyire MG, Magure TM, Madziwa CF. Menstrual cups as a menstrual management method for low socioeconomic status women and girls in Zimbabwe: a pilot study. *Women's Reprod Health* 2018; **5**: 59-65.
4. Higgins JP, Thompson SG, Deeks JJ, Altman DG. Measuring inconsistency in meta-analyses. *BMJ* 2003; **327**: 557-60.
5. Deeks JJ, Higgins JP, Altman DG. Analysing data and undertaking meta-analyses. In: Higgins JP, Green S, editors. Cochrane handbook for systematic reviews of interventions (version 5.1.0): The Cochrane Collaboration; 2011.
6. Public Health England. PHE Technical Guide - Funnel Plots. 2018. https://fingertips.phe.org.uk/profile/guidance (accessed May 28, 2019).
7. Sterne JA, Gavaghan D, Egger M. Publication and related bias in meta-analysis: power of statistical tests and prevalence in the literature. *J Clin Epidemiol* 2000; **53**: 1119-29.
8. Critical Appraisal Skills Programme (CASP). CASP Checklists. 2017. http://www.casp-uk.net/casp-tools-checklists (accessed May 28, 2019).
9. Dixon-Woods M, Agarwal S, Jones D, Young B, Sutton A. Synthesising qualitative and quantitative evidence: a review of possible methods. *J Health Serv Res Policy* 2005; **10**: 45-53.
10. Thomas J, Harden A. Methods for the thematic synthesis of qualitative research in systematic reviews. *BMC Med Res Methodol* 2008; **8**: 45.
11. Menstrual Cup Master List. 2017. https://menstrualcups.wordpress.com/menstrual-cup-master-list/ (accessed May 28, 2019).
12. Howard C, Rose CL, Trouton K, et al. FLOW (finding lasting options for women): multicentre randomized controlled trial comparing tampons with menstrual cups. *Can Fam Physician* 2011; **57**: e208-15.
13. Beksinska ME, Smit J, Greener R, et al. Acceptability and performance of the menstrual cup in South Africa: a randomized crossover trial comparing the menstrual cup to tampons or sanitary pads. *J Womens Health* 2015; **24**: 151-8.
14. North B, Oldham M. Preclinical, Clinical, and Over-the-Counter Postmarketing Experience with a New Vaginal Cup: Menstrual Collection. *J Womens Health* 2011; **20**: 303-11.
15. Mazgaj M, Yaramenka K, Malovana O. Comparative life cycle assessment of sanitary pads and tampons. 2006. http://docplayer.net/39797321-Comparative-life-cycle-assessment-of-sanitary-pads-and-tampons.html (accessed May 28, 2019).
16. Munro MG, Critchley HO, Fraser IS. The FIGO systems for nomenclature and classification of causes of abnormal uterine bleeding in the reproductive years: who needs them? *Am J Obstet Gynecol* 2012; **207**: 259-65.
17. Beksinska M, Smit J, Greener R, et al. `It gets easier with practice'. A randomised cross-over trial comparing the menstrual cup to tampons or sanitary pads in a low resource setting. *Eur J Contracept Reprod Health Care* 2016; **21**: 138.
18. Beksinska M, Smit J, Greener R, Maphumulo V, Mabude Z. Better menstrual management options for adolescents needed in South Africa: What about the menstrual cup? *S Afr Med J* 2015; **105**: 331.
19. Hoffmann V, Adelman S, Sebastian A. Learning by doing something else: Experience with alternatives and adoption of a high-barrier menstrual hygiene technology. 2014. https://docplayer.net/41533102-Learning-by-doing-something-else-experience-with-alternatives-and-adoption-of-a-high-barrier-menstrual-hygiene-technology.html (accessed May 28, 2019).
20. Oster E, Thornton R. Menstruation and Education in Nepal. 2009. http://www.nber.org/papers/w14853 (accessed May 28, 2019).
21. Oster E, Thornton R. Determinants of Technology Adoption: Private Value and Peer Effects in Menstrual Cup Take-Up 2009. http://www.nber.org/papers/w14828 (accessed May 28, 2019).
22. Oster E, Thornton R. Menstruation, sanitary products, and school attendance: evidence from a randomized evaluation. *Am Econ J Appl Econ* 2011; **3**: 91-100.
23. Oster E, Thornton R. Determinants of technology adoption: Peer effects in menstrual cup up-take. *J Eur Econ Assoc* 2012; **10**: 1263-93.
24. African Population and Health Research Center. Attitudes towards, and acceptability of, menstrual cups as a method for managing menstruation: experiences of women and school girls in Nairobi, Kenya. 2010. http://www.susana.org/_resources/documents/default/2-984-policy-brief-no-21-2010-attitudes-and-acceptability.pdf (accessed May 28, 2019).
25. African Population and Health Research Center. Use of menstrual cup by adolescent girls and women: potential benefits and key challenges. 2010. https://www.susana.org/en/knowledge-hub/resources-and-publications/library/details/985 (accessed May 28, 2019).
26. African Population and Health Research Center. Experiences and problems with menstruation among poor women and schoolgirls in Nairobi, Kenya. 2010. http://www.communityledtotalsanitation.org/sites/communityledtotalsanitation.org/files/PolicyBrief_Mooncups_Kenya.pdf (accessed May 28, 2019).

27. Care International Uganda. Ruby Cups: Girls in Imvepi Refugee Settlement Taking Control. 2018. http://womena.dk/ruby-cups-girls-in-imvepi-refugee-settlement-taking-control/ (accessed May 28, 2019).
28. Cattanach JF. The Gynaeseal diaphragm tampon. *Med J Aust* 1990; **152**: 52-3.
29. Cattanach JF. A diaphragm tampon applied to an ovulation method in a birth control system. *Contracept* 1991; **44**: 607-21.
30. Cheng M, Kung R, Hannah M, Wilansky D, Shime J. Menses cup evaluation study. *Fertil Steril* 1995; **64**: 661-3.
31. Chintan S, Dipesh P, Maitri P. Use of Flow care Menstrual Cups over conventional menstrual products in India. *Int J Adv Res Dev* 2017; **2**: 78-82.
32. Ganyaglo GYK, Ryan N, Park J, Lassey AT. Feasibility and acceptability of the menstrual cup for non-surgical management of vesicovaginal fistula among women at a health facility in Ghana. *Plos One* 2018; **13**: e0207925.
33. Ryan N. Feasibility, acceptability, and appropriateness of the vaginal menstrual cup for short term non-surgical management of vesicovaginal fistula (VVF) among potential users and stakeholders. *Implement Sci* 2018; **13**: S80.
34. Gleeson N, Devitt M, Buggy F, Bonnar J. Menstrual blood loss measurement with gynaeseal. *Aust N Z J Obstet Gynaecol* 1993; **33**: 79-80.
35. Kakani CR, Bhatt JK. Study of adaptability and efficacy of menstrual cup in managing menstrual health and hygiene. *Int J Reprod Contracept Obstet Gynecol* 2017; **6**: 3045-53.
36. Madziyire MG, Magure TM, Madziwa CF. The safety of menstrual cups in women of low socio-economic status in Zimbabwe: pilot study. *Cent Afr J Med* 2018; **64**: 59-65.
37. Parker J, Bushell RW, Behrman SJ. Hygienic control of menorrhagia: use of rubber menstrual cup. *Int J Fertil* 1966; **9**: 619-21.
38. Pena EF. Menstrual protection. Advantages of the menstrual cup. *Obstet Gynecol* 1962; **19**: 684-7.
39. Shihata A, Brody S. An innovative, reusable menstrual cup that enhances the quality of women's lives during menstruation. *Br J Med Med Res* 2014; **4**: 3581-90.
40. Stewart K, Greer R, Powell M. Women's experience of using the Mooncup. *J Obstet Gynaecol* 2010; **30**: 285-7.
41. Tellier M, Hyttel M, Gad M. Assessing acceptability and hygienic safety of menstrual cups as a menstrual management method for vulnerable young women in Uganda Red Cross Society's Life Planning Skills Project 2012. http://womena.dk/wp-content/uploads/2015/11/Menstrual-Cups-_-WoMena-_-Uganda-Pilot-Study-Report-Dec-2012-new-version.pdf (accessed May 28, 2019).
42. Wiebe ER, Trouton KJ. Does using tampons or menstrual cups increase early IUD expulsion rates? *Contracept* 2012; **86**: 119-21.
43. Averbach S, Sahin-Hodoglugil N, Musara P, Chipato T, van der Straten A. Duet for menstrual protection: a feasibility study in Zimbabwe. *Contracept* 2009; **79**: 463-8.
44. Borowski A. Are American women turning to reusable and greener menstrual products due to health and environmental pollution concerns? 2011. http://scholarworks.rit.edu/theses/544/ (accessed May 28, 2019).
45. Grose RG, Grabe S. Sociocultural attitudes surrounding menstruation and alternative menstrual products: the explanatory role of self-objectification. *Health Care Women Int* 2014; **35**: 677-94.
46. Stewart K, Powell M, Greer R. An alternative to conventional sanitary protection: would women use a menstrual cup? *J Obstet Gynaecol* 2009; **29**: 49-52.
47. Goldberg L, Elsamra S, Hutchinson-Colas J, Segal S. Delayed Diagnosis of Vesicouterine Fistula After Treatment for Mixed Urinary Incontinence: Menstrual Cup Management and Diagnosis. *Female Pelvic Med Reconstr Surg* 2016; **22**: e29-31.
48. Russell KW, Robinson RE, Mone MC, Scaife CL. Enterovaginal or Vesicovaginal Fistula Control Using a Silicone Cup. *Obstet Gynecol* 2016; **128**: 1365-8.
49. Hyttel M, Thomsen CF, Luff B, Storrusten H, Nyakato VN, Tellier M. Drivers and challenges to use of menstrual cups among schoolgirls in rural Uganda: a qualitative study. *Waterlines* 2017; **36**: 109-24.
50. Sundqvist J. A cup of freedom? A study of menstrual cup's impact on girls' capabilities. 2015. http://www.diva-portal.org/smash/record.jsf?pid=diva2%3A783661&dswid=-5356 (accessed May 28, 2019).
51. Mason L, Laserson K, Oruko K, et al. Adolescent schoolgirls' experiences of menstrual cups and pads in rural western Kenya: a qualitative study. *Waterlines* 2015; **34**: 15-30.
52. van Eijk AM, Laserson KF, Nyothach E, et al. Use of menstrual cups among school girls: longitudinal observations nested in a randomised controlled feasibility study in rural Western Kenya. *Reprod Health* 2018; **15**: 139.
53. Shihata AA. Device and method for menstrual blood collection. 2014. https://patents.google.com/patent/US20130110060 (accessed May 28, 2019).
54. Nonfoux L, Chiaruzzi M, Badiou C, et al. Impact of currently marketed tampons and menstrual cups on Staphylococcus aureus growth and TSST-1 production in vitro. *Appl Environ Microbiol* 2018; **84**: e0035-18.
